# Insights into the Degradation Mechanism of Aqueous LiMn_2_O_4_–Cathode Interfaces by Computational Vibrational Spectroscopy

**DOI:** 10.1002/anie.3712910

**Published:** 2026-09-23

**Authors:** K. Nikolas Lausch, Redouan El Haouari, Philipp Schienbein, Jörg Behler

**Affiliations:** ^1^ Lehrstuhl für Theoretische Chemie II Ruhr‐Universität Bochum Bochum Germany; ^2^ Research Center Chemical Sciences and Sustainability Research Alliance Ruhr Bochum Germany

**Keywords:** computational spectroscopy, Li‐ion batteries, lithium manganese oxide, machine learning potential, solid–liquid interfaces

## Abstract

${\mathrm{LiMn}}_{2}O_{4}$ (LMO) is a versatile and earth‐abundant Li intercalation compound of high interest for sustainable energy storage and conversion. Yet, its broader adoption as a cathode in Li‐ion batteries or as a water‐splitting electrocatalyst is hindered by severe degradation reactions at the solid–electrolyte interface. This instability is rooted in its mixed‐valence ${\mathrm{Mn}}^{3+}$/${\mathrm{Mn}}^{4+}$ electronic structure, which is governed by the Li content. Still, the atomistic link between the lithiation state and interfacial degradation remains elusive. Here, we investigate aqueous LMO interfaces across varying lithiation states by atomistic simulations using an ab initio‐quality machine learning potential. We demonstrate that the local Li content determines surface Mn oxidation states and reveal their decisive role in interfacial acid–base chemistry. By analyzing the vibrational features of interfacial species, we identify the O–H stretching band as a sensitive spectroscopic probe of the surface electronic structure. Distinct, oxidation‐state‐dependent shifts reveal a fundamental vulnerability of the mixed‐valence spinel surface: the coexistence of Lewis‐acidic ${\mathrm{Mn}}^{4+}$ centers and oxygen sites neighboring ${\mathrm{Mn}}^{3+}$, which are susceptible to electrophilic attack. These findings provide a compelling atomistic rationale extending the conventional ${\mathrm{Mn}}^{3+}$‐centric view of acid‐promoted Mn dissolution toward a dual‐site mechanism driven by LMO's mixed‐valent nature.

## Graphical abstract

${\mathrm{LiMn}}_{2}O_{4}$ is of high interest for sustainable energy storage and conversion. Its mixed‐valence ${\mathrm{Mn}}^{3+}$/${\text{Mn}}^{4+}$ structure drives site‐specific behavior at aqueous interfaces, featuring proton‐generating ${\text{Mn}}^{4+}$ centers and electron‐rich ${\mathrm{Mn}}^{3+}$ sites that render the surrounding oxygen lattice highly nucleophilic. This duality opens a pathway for acid‐promoted Mn dissolution by locally generating the requisite acidic conditions.

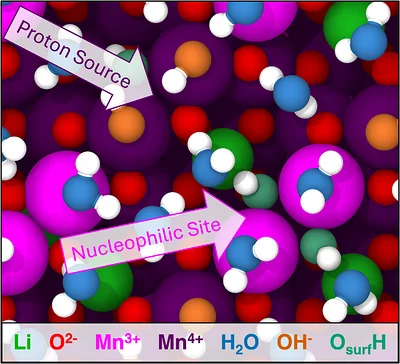

## 1 Introduction

Li‐ion batteries based on spinel ${\text{LiMn}}_{2}O_{4}$ (LMO) cathodes represent a sustainable and cost‐effective solution for large‐scale energy storage [1, 2, 3]. However, their widespread adoption is hindered by a limited cycle‐life caused by severe degradation reactions [2, 3, 4, 5]. Among these, the dissolution of Mn‐ions into the electrolyte is considered the primary failure mechanism [4, 5, 6]. This dissolution is conventionally attributed to the Hunter disproportionation reaction $\left[2{\text{Mn}}_{\text{(s)}}^{3+}\to {\text{Mn}}_{\text{(s)}}^{4+}+{\text{Mn}}_{\text{(aq)}}^{2+}\right]$, a process initiated by acidic species ($H^{+}$) in the electrolyte [5, 6, 7]. The required protons can, for example, originate from the hydrolysis of the ${\text{LiPF}}_{6}$ conductive salt by traces of water, forming HF [4, 5, 6].

The susceptibility of LMO to this acid attack is fundamentally linked to its electronic structure. High‐spin (hs)‐${\text{Mn}}^{3+}$ ions are subject to strong Jahn–Teller (J–T) distortion due to a single electron in the antibonding $e_{g}$ orbital ($e_{g}^{1}$) of the octahedral ligand field [4]. This electronic configuration leads to a simultaneous elongation of the Mn–O bonds and an increase in the Lewis basicity of the axial oxygen atoms, rendering them highly susceptible to electrophilic attack by protons [4].

Therefore, strategies to prevent Mn dissolution have focused on optimizing the electrolyte to suppress HF formation [5] and stabilizing the lattice against these J–T distortions. Common approaches include doping with low‐valent metal ions (e.g., ${\text{Ni}}^{2+}$, ${\text{Al}}^{3+}$) to keep the average Mn oxidation state above +3.5 [3, 8, 9]. This effectively minimizes the concentration of reactive ${\text{Mn}}^{3+}$ species and suppresses the cooperative J–T effect.

However, several experimental observations have challenged the ${\text{Mn}}^{3+}$‐centric view of the Hunter mechanism. Notably, Mn dissolution is found to accelerate during charging of Li‐ion batteries, that is, delithiation of the cathodes [5, 10, 11, 12, 13, 14]. Since, upon delithiation, the manganese atoms in the lattice are oxidized to ${\text{Mn}}^{4+}$ [13, 15], the concentration of susceptible ${\text{Mn}}^{3+}$ species is progressively depleted, which in principle should decrease Mn dissolution at high battery charge states. These seemingly contradictory observations indicate that the dissolution process is considerably more complex than initially expected and fundamentally depends on the lithiation state. While the evolution of the LMO surface structure across various lithiation states has been investigated using ex situ characterization and static vacuum‐slab models [13], recent efforts have shifted toward in situ and dynamic investigations. For example, the Mn dissolution process at varying states of charge has been probed using in situ ultraviolet–visible spectroscopy and ab initio molecular dynamics [14, 16]. Despite these advances, the atomistic link between the lithiation state and interfacial dynamics remains poorly understood [6]. Specifically, changes in the complex acid–base chemistry at the LMO–electrolyte interface across different states of charge have remained largely unexplored.

Ultimately, to unravel the degradation processes at LMO–electrolyte interfaces, a detailed atomistic description of the system is required, which takes the complex interplay between alternating surface charge states, solvent dynamics, and acid–base chemistry into account. However, experimentally isolating these complex interfacial processes poses a significant challenge, and established ab initio methods are computationally too demanding to sample the necessary timescales of large solid–liquid interface models.

In this work, we overcome these limitations by using a high‐dimensional neural network potential (HDNNP) [17, 18, 19, 20] to study aqueous LMO interfaces with ab initio accuracy in extended Molecular Dynamics (MD) simulations. By systematically varying the Li content, we model the LMO–water interface across different states of charge. We note that Li intercalation is a coupled ion–electron transfer process [21]; therefore, the LMO–cathode remains charge neutral across all states of charge. As the open‐circuit voltage (OCV) depends on the lithium chemical potential $\mu_{\mathrm{Li}}$ in the cathode—where Li deintercalation lowers $\mu_{\mathrm{Li}}$ and increases the cell voltage—our models represent the LMO–cathode at distinct mean OCVs along the battery charging curve [22, 23].

To investigate the changes in the interfacial acid–base chemistry across different states of charge, we utilize interfacial water as a sensitive vibrational probe. By analyzing the shifts of O–H stretching vibrations of distinct interfacial species via vibrational density of states (VDOS) decomposition, we shed light on the characteristics of surface acid–base chemistry, allowing us to identify the spectral signatures of degradation‐prone regimes. Finally, we connect these vibrational frequency shifts to the underlying thermodynamic driving forces by calculating the potentials of mean force (PMFs) for the elementary steps of water dissociation across all investigated interfaces.

## 2 Results and Discussion

### 2.1 High‐Dimensional Neural Networks

To accurately capture the complex electronic structure of the aqueous LMO interface across different lithiation states, we have first constructed an HDNNP trained on spin‐polarized Density Functional Theory (DFT) data, which was generated using the local hybrid functional PBE0r [24, 25] including the Tkatchenko–Scheffler (TS) dispersion correction [26]. This model generalizes our previously reported HDNNPs for bulk LMO [25, 27] and stoichiometric LMO–water interfaces [28] to a wide range of lithiation states.

To minimize computational cost while maximizing configuration space coverage, we employed an active learning workflow based on Query‐by‐Committee to expand the existing reference dataset, incorporating LMO–water interfaces with varying lithium content (${\text{Li}}_{x_{\text{Li}}}{\text{Mn}}_{2}O_{4}$, with $x_{\text{Li}}$ from 0 to 1) [29, 30, 31]. To monitor the reliability of the simulations and to enable uncertainty quantification, the final potential was deployed as a committee of eight HDNNPs (c‐HDNNP) [32] with varying initial weight parameters and train/test splits. The individual committee members exhibit energy and force root‐mean‐squared errors (RMSEs) of 2.1–2.2 meV/atom and 183–184 meV/Å for the training sets, and 2.4–2.5 meV/atom and 184–185 meV/Å for the test sets; see Section S1.4 for details. These values are similar to neural network potentials recently reported for other aqueous transition‐metal oxide interfaces [31, 33, 34].

Because HDNNPs do not explicitly provide information about the electronic degrees of freedom, we integrated an additional high‐dimensional neural network for spin prediction (HDNNS) [15]. Trained on DFT‐derived atomic spins, this model can predict the local spin state of individual Mn‐ions based on their local geometric environment, enabling the assignment of oxidation states via trajectory post‐processing. The HDNNS exhibits training and test RMSEs of 0.02 ℏ for the atomic spins. Given that ${\text{Mn}}^{4+}$ and hs‐${\text{Mn}}^{3+}$ possess absolute spins of 1.5 and 2.0 ℏ, respectively, this precision enables a clear differentiation between the two states. As demonstrated in earlier work [15, 28], this approach conserves the total charge for both bulk and interfacial systems, providing the link between the atomistic structure and local electronic state as required for the present simulations.

### 2.2 Interfacial Structure and Chemistry

The aqueous ${\text{LiMn}}_{2}O_{4}$(001) interfaces have been represented by 4×4 supercells. These supercells comprise 40 atomic layers in total, featuring an alternating Li and ${\mathrm{MnO}}_{2}$ layer sequence that yields a slab thickness of ≈40 Å. The resulting slabs were placed in contact with water films of 40 Å thickness containing 1500 water molecules (see Section S2.1 for detailed system setups and equilibrium configurations). To simulate varying lithiation states ($x_{\text{Li}}=1.0$, 0.5, and 0.0), Li ions were systematically removed from the spinel framework.

As the Li diffusion barrier of ≈0.5 eV prevents spontaneous equilibration on MD timescales [27, 35], the $x_{\mathrm{Li}}=0.5$ state was explicitly sampled using two independent configurations. To capture known Li–Li repulsion‐driven ordering [27], the Li ions were arranged to exclusively populate either one of the two interpenetrating face‐centered cubic (*fcc*) sub‐lattices of the diamond‐like *8a* tetrahedral sites [9]. These two configurations, denoted ${\mathrm{Li}}_{0.5}^{A}{\mathrm{Mn}}_{2}O_{4}$ and ${\mathrm{Li}}_{0.5}^{B}{\mathrm{Mn}}_{2}O_{4}$, represent the two possible structural arrangements that maximize Li–Li nearest‐neighbor distances in the half‐lithiated state. Removing all Li atoms results in the $\lambda \text{-Mn}O_{2}$ spinel‐type structure [7, 36].

To counteract the well‐known over‐structuring of liquid water inherent to PBE‐derived hybrid functionals [37, 38, 39], all simulations were performed at an elevated temperature of 350 K [33, 39, 40]. As detailed in the Section S1.8, this accurately recovers the structural and dynamical properties of water at ambient conditions.

All initial slab configurations were equilibrated in the isobaric–isothermal (NpT) ensemble for 1ns, followed by 3ns of production sampling with configurations saved at 0.1ps intervals. Statistical uncertainties for all reported properties were estimated via block averaging: the 3ns trajectory was partitioned into three non‐overlapping 1ns blocks, and the standard error of the mean (including Bessel's correction) was calculated from the variance across these blocks. The stability of the simulations was verified by monitoring the energy and maximum force component standard deviation of the c‐HDNNP committee members, which stayed consistently within the expected range defined by the model's RMSE (see Section S2.2.1 for details).

Density profiles (see Section S2.6) confirm that in general the slab interior maintains bulk‐like structural properties, while the liquid phase equilibrates to a density of $1.01\;g\;{\mathrm{cm}}^{-3}$ ($56.1\;\mathrm{mol}\;L^{-1}$) in the center of the water region, which aligns with the density of bulk water and confirms that the water slab is sufficiently thick to decouple the two interfaces.

To characterize the electronic structure, all trajectories were analyzed using the HDNNS. Applying the classification criteria defined in our previous work [15, 28], we assigned an integer oxidation state label (${\text{Mn}}^{3+}$ or ${\text{Mn}}^{4+}$) to each Mn site in every frame based on the predicted atomic spin. The resulting mean Mn oxidation states were found to be in excellent agreement with the values expected for the respective Li concentrations, thereby confirming charge conservation and the robustness of the c‐HDNNP and HDNNS (see Section S2.2.2 for detailed analysis).

While the ratio of the mixed‐valence ${\text{Mn}}^{3+}/{\text{Mn}}^{4+}$ ions in the slab center corresponds to the bulk value for all investigated systems, surface layers exhibit distinct electronic structure characteristics governed by the respective surface termination and interface structure (see Section S2.3). The average surface layer Mn oxidation states ${\bar{q}}_{\mathrm{Mn}}$ are summarized in Table 1.

**TABLE 1:** Average surface layer Mn oxidation states ${\bar{q}}_{\mathrm{Mn}}$, predicted via HDNNS, corresponding adsorbed hydroxide coverage $\Theta_{\mathrm{OH}}$ per coordinatively unsaturated Mn site, and water dissociation degrees α at the top and bottom surfaces of slabs with different Li contents.

Surface	${\bar{q}}_{\mathrm{Mn}}$ / e	$\Theta_{\mathrm{OH}}$	α / %
${\mathrm{LiMn}}_{2}O_{4}\left(001\right)$	3.06(1)	0.07(2)	4.0(9)
${\mathrm{LiMn}}_{2}O_{4}\left(00\bar{1}\right)$	3.362(1)	0.352(3)	19.3(2)
${\mathrm{Li}}_{0.5}^{A}{\mathrm{Mn}}_{2}O_{4}\left(001\right)$	3.74(4)	0.46(3)	24(2)
${\mathrm{Li}}_{0.5}^{A}{\mathrm{Mn}}_{2}O_{4}\left(00\bar{1}\right)$	3.71(2)	0.47(2)	25(1)
${\mathrm{Li}}_{0.5}^{B}{\mathrm{Mn}}_{2}O_{4}\left(001\right)$	3.64(2)	0.395(9)	20.8(5)
${\mathrm{Li}}_{0.5}^{B}{\mathrm{Mn}}_{2}O_{4}\left(00\bar{1}\right)$	3.99(1)	0.70(2)	36.0(6)
$\lambda -{\mathrm{MnO}}_{2}\left(001\right)$	3.9977(3)	0.641(6)	33.6(4)
$\lambda -{\mathrm{MnO}}_{2}\left(00\bar{1}\right)$	3.998(2)	0.615(5)	32.2(3)

*Note*: Data were obtained from 3 ns MD trajectories in the *NpT* ensemble. Uncertainties in parentheses represent the standard error of the mean (including Bessel's correction), calculated by block averaging the simulation in three 1 ns windows.

Figure 1 presents a selection of time‐averaged surface configurations for facets with varying Li content (remaining facets are shown in Figure S23). A clear trend is evident: the concentration of J–T active ${\text{Mn}}^{3+}$ species at the surface is strongly correlated to the local Li content. In the fully lithiated state ($x_{\text{Li}}=1.0$) with Li termination, the surface is rich in ${\text{Mn}}^{3+}$. Delithiation ($x_{\text{Li}}\leq 0.5$) systematically increases the average Mn oxidation state, depleting the surface of J–T active centers. This local electronic compensation, which decays into the bulk over several atomic layers (see Section S2.3), highlights the material's ability to compensate surface charge imbalances locally through oxidation state changes rather than via ionic reconstruction.

**FIGURE 1:**
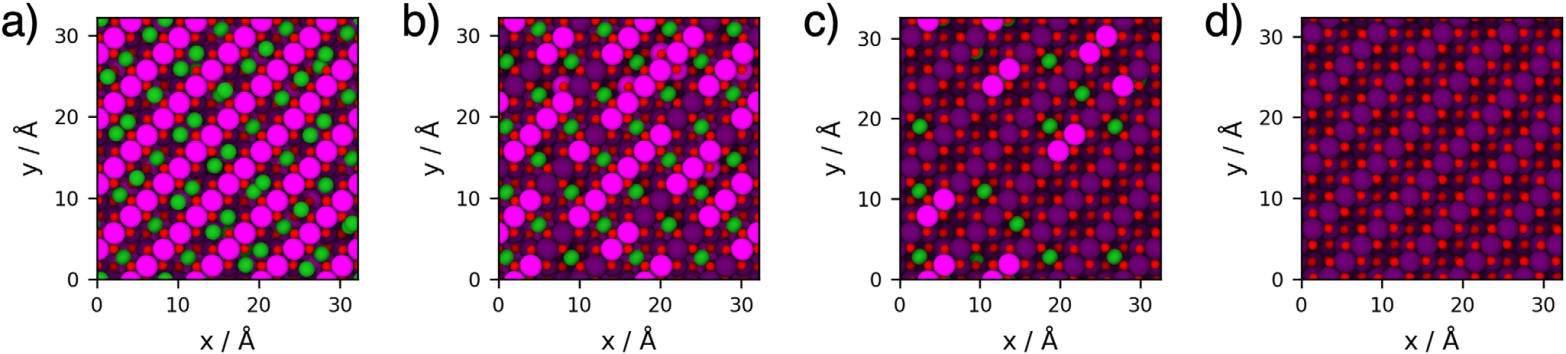
Time‐averaged surface configurations of LMO (001) slab facets with different Li content. Each panel shows the mean atomic positions sampled over 3 ns of *NpT* MD simulation, with the aqueous phase omitted for clarity. To account for thermal fluctuations in the *NpT* ensemble, lattice parameters were averaged over the trajectory and atomic positions rescaled accordingly. Surface environments are labeled as: (a) ${\mathrm{LiMn}}_{2}O_{4}$(001), (b) ${\mathrm{LiMn}}_{2}O_{4}(00\bar{1}$), (c) ${\mathrm{Li}}_{0.5}^{A}$ ${\mathrm{Mn}}_{2}O_{4}$(001), and (d) λ‐${\mathrm{MnO}}_{2}$(001). Atomic species are color‐coded as follows: O (red), Li (green), ${\mathrm{Mn}}^{3+}$ (magenta), and ${\mathrm{Mn}}^{4+}$ (dark purple). Oxidation states were assigned by time‐averaging the atomic spins predicted by the HDNNS for each site, followed by a subsequent classification as detailed in Section S1.5. The surfaces were rendered using OVITO Pro (version 3.14.1) [41].

Water dissociation is observed at all interfaces, evidenced by the accumulation of hydronium ($H_{3}O^{+}$) and protonated surface oxygen sites as well as hydroxide (${\text{OH}}^{-}$) species near the surface (concentration profiles shown in Section S2.7). Comparing these profiles with the surface electronic structure reveals a direct correlation between the dissociation degree α, that is, the ratio of dissociated water molecules to the total number of water molecules in the first hydration layer—defined as the region between the surface and the first minimum in the liquid‐phase oxygen density profile—and the average Mn oxidation state: as more surface Mn ions are oxidized to ${\text{Mn}}^{4+}$ (surface delithiation), water dissociation increases significantly (Table 1).

To analyze the local environment of these dissociation products, we projected the trajectory data into 2D density maps of the first hydration layer on the time‐averaged surface structure (Figure 2). In the fully lithiated, Li‐terminated limit ($x_{\text{Li}}=1.0$, Figure 2a), the probability density of the water molecules in the first hydration layer is highly diffuse, indicating weak surface interactions. Due to surface cleavage, the exposed Mn ions are coordinatively unsaturated sites (CUS) missing a single lattice oxygen. Consequently, interfacial water molecules exhibit a propensity to adsorb at these metal centers, acting as ligands to restore the preferred octahedral coordination environment, which explains the increased density at the Mn sites. Dissociated species are rare, yielding an average hydroxyl coverage ($\Theta_{\mathrm{OH}}$) of ≈0.07 per Mn CUS (Table 1); when present, ${\text{OH}}^{-}$ coordinates to a Mn CUS while the corresponding proton binds to a nearby surface oxygen ($O_{\text{surf}}H$).

**FIGURE 2:**
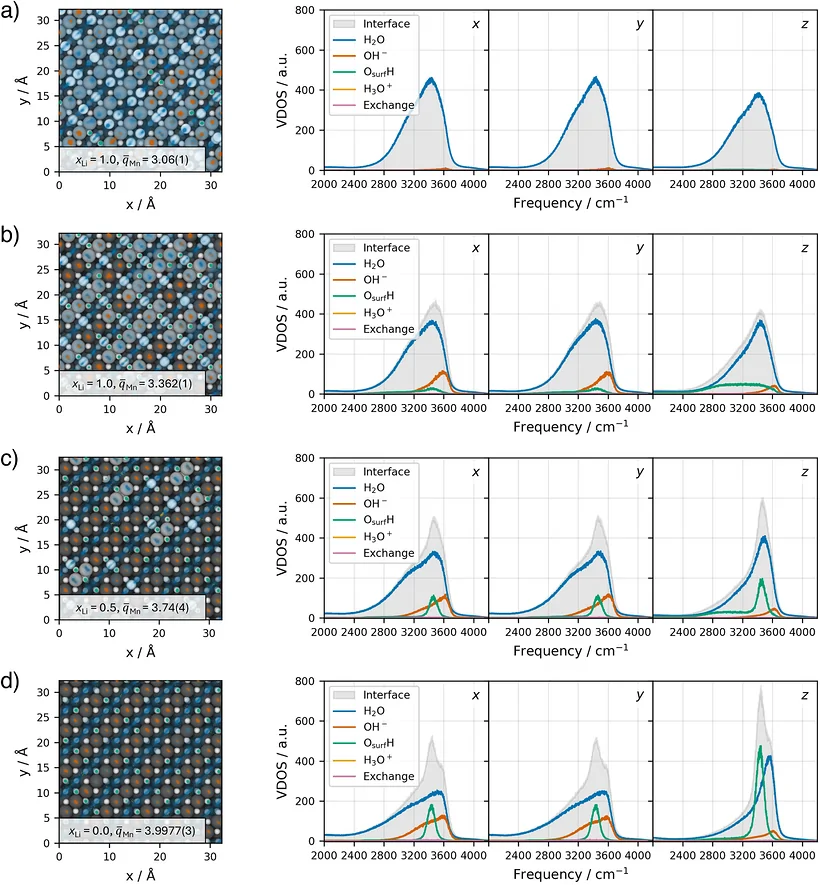
Decomposition of the VDOS into chemical species contributions. Each row represents a different surface structure with decreasing Li content from top to bottom: (a) ${\mathrm{LiMn}}_{2}O_{4}$(001), (b) ${\mathrm{LiMn}}_{2}O_{4}(00\bar{1}$), (c) ${\mathrm{Li}}_{0.5}^{A}{\mathrm{Mn}}_{2}O_{4}$ (001), and (d) λ‐${\mathrm{MnO}}_{2}$(001). Left: Real‐space probability distribution of chemical species within the first hydration layer. Species are color‐coded as follows: water $H_{2}O$ (blue), interfacial hydroxide ${\mathrm{OH}}^{-}$ (orange), protonated surface oxygen $O_{\text{surf}}H$ (bluish green), and hydronium $H_{3}O^{+}$ (yellow). The time‐averaged surface atom positions are shown in grayscale: ${\mathrm{Mn}}^{4+}$ (dark gray, large radius), ${\mathrm{Mn}}^{3+}$ (gray, large radius), O (light gray, small radius), and Li (white, intermediate radius). The surfaces were rendered using OVITO Pro (version 3.14.1) [41]. Right: Corresponding species decomposed spectral densities (VDOS / a.u.). The exchange term (purple) represents the sum of the cross‐correlation components between all species. The gray background represents the overall VDOS of the interface region.

As the surface is delithiated and the Mn ions are oxidized (Figure 2a–d), the structural ordering of interfacial water increases. Distinct “hot spots” of probability density emerge, corresponding to water and hydroxide species tightly bound to Mn sites, as well as water molecules localized within channels between rows of surface Mn ions along the [110] direction. Notably, ${\text{OH}}^{-}$ species preferentially coordinate to ${\text{Mn}}^{4+}$ ions, consistent with our previous findings for stoichiometric interfaces [28]. In the fully delithiated λ‐${\text{Mn}}_{2}O_{4}$ limit ($x_{\text{Li}}=0.0$), the surface consists exclusively of ${\text{Mn}}^{4+}$ ions, which increases the average hydroxyl coverage to $\Theta_{\mathrm{OH}}\approx 0.64$ per Mn CUS. This progression suggests that the enhanced Lewis acidity of the ${\text{Mn}}^{4+}$ ions drives the dissociation reaction by polarizing the coordinated water molecules, thereby weakening the intramolecular O–H bond and facilitating proton transfer to an adjacent surface oxygen site.

Even in the fully oxidized state (Figure 2d), molecular water persists at the interface, localizing with high probability within the [110] channels between surface Mn ions. To analyze the interfacial bonding topology, we determined the molecular orientation relative to the outward‐pointing surface normal (n=[001] for the upper interface and $n=[00\bar{1}]$ for the lower interface) by defining the angle θ between the water bisector and n. The resulting 2D orientation histograms (Figure 3) reveal a distinct bimodal distribution, with molecules facing either *toward* (cosθ<0) or *away* (cosθ>0) from the solid. Water molecules residing in the [110] channels predominantly point *toward* the surface, acting as hydrogen‐bond donors to the surface oxygen atoms. Conversely, water molecules adsorbed atop Mn sites point away from the surface, consistent with the formation of coordinative bonds between the water oxygen atoms and the Lewis‐acidic metal centers.

**FIGURE 3:**
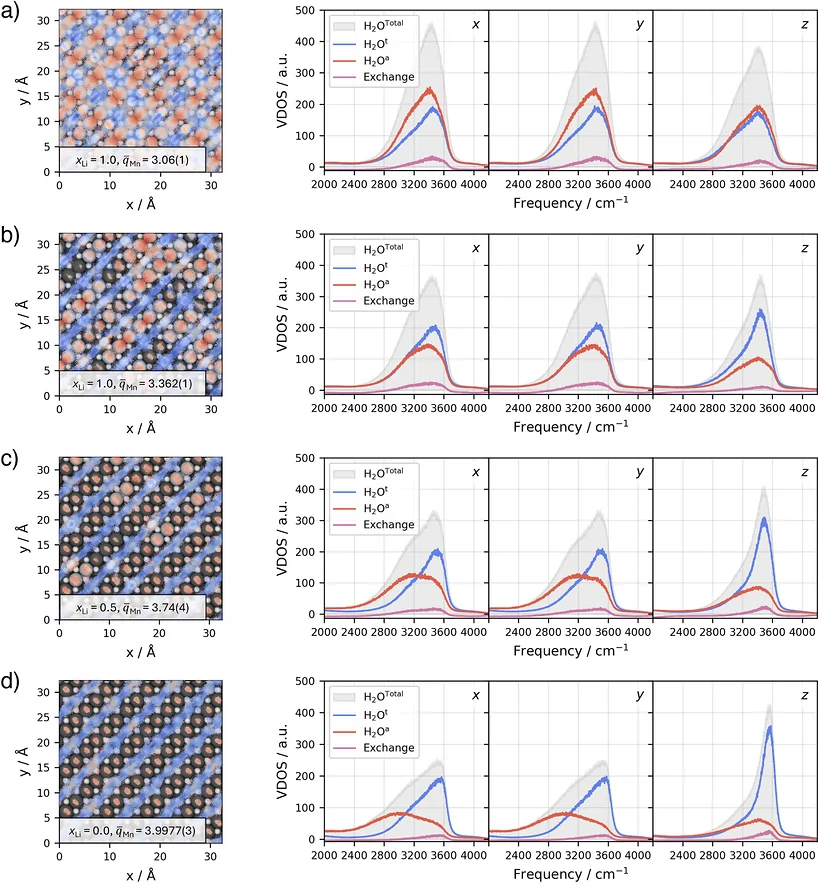
Correlation between interfacial water orientation and vibrational dynamics. Each row represents a different surface structure with decreasing Li content from top to bottom: (a) ${\mathrm{LiMn}}_{2}O_{4}$(001), (b) ${\mathrm{LiMn}}_{2}O_{4}(00\bar{1}$), (c) ${\mathrm{Li}}_{0.5}^{A}{\mathrm{Mn}}_{2}O_{4}$ (001), and (d) λ‐${\mathrm{MnO}}_{2}$(001). Left: Real‐space probability distribution of first layer water molecules, color‐coded by molecular orientation. The orientation is defined by the angle θ between the water bisector and the outward‐pointing surface normal, represented by a diverging color gradient from blue (cosθ=−1, pointing toward the surface) to red (cosθ=1, pointing away), with white indicating a surface‐parallel (flat) orientation. The time‐averaged surface atom positions are shown in grayscale: ${\mathrm{Mn}}^{4+}$ (dark gray, large radius), ${\mathrm{Mn}}^{3+}$ (gray, large radius), O (light gray, small radius), and Li (white, intermediate radius). The surfaces were rendered using OVITO Pro (version 3.14.1) [41]. Right: Corresponding water species spectral density (VDOS/a.u.) decomposed by the same orientation criteria. The exchange term (purple) represents the sum of the cross‐correlation components between the two orientations.

### 2.3 Surface Vibrational Spectroscopy

Surface‐sensitive or ‐specific vibrational spectroscopies [42, 43, 44, 45, 46], such as Sum‐Frequency Generation (SFG), Attenuated Total Reflection (ATR)‐Infrared (IR) spectroscopy, or Surface Enhanced Raman Scattering (SERS), are powerful tools to uncover adsorption motifs and to quantify bonding and interaction strengths at solid–liquid interfaces. As a first‐order approximation to these experimental vibrational signatures, the VDOS can be calculated directly from the atomic velocities obtained in MD simulations [47]. For instance, for oxide–water interfaces, it has been shown that hydrogen bonds donated to and accepted from the oxide surface can be distinguished by their distinct spectral features in the VDOS [48].

Here, we analyze the VDOS of interfacial H atoms to understand the evolution from a stable, low‐reactivity surface to a highly dissociative regime. Specifically, we aim to extract the distinct spectral signatures of various interfacial species, such as $H_{2}O$, ${\mathrm{OH}}^{-}$, $H_{3}O^{+}$, and protonated surface oxygen sites ($O_{\text{surf}}H$), to observe how these signatures change with the degree of lithiation.

To rigorously separate these spectral contributions, we adapt the Cross‐Correlation Analysis (CCA) decomposition scheme [49, 50, 51]. The CCA has been introduced previously to decompose IR spectra into individual molecular  [49] or atomic contributions [50, 51] based on topological classifiers.

In the original definition, the atomic CCA requires velocities and either atomic polar tensors or polarizability gradient tensors to obtain IR or Raman spectra, respectively [51]. However, it is possible to set these tensors to unity and neglect cross‐correlations between different atoms to allow for a rigorous decomposition of the VDOS into species contributions.

The total VDOS, I(ω), is the Fourier transform of the mean of all atomic velocity autocorrelation functions (VACFs),

(1) $$C\left(t\right)=\frac{1}{N}\sum_{i=1}^{N}\left\langle v_{i}\left(0\right)\cdot v_{i}\left(t\right)\right\rangle \;,$$

where $v_{i}$ is the velocity vector of atom i and N is the number of atoms. To decompose this correlation into specific topological groups a, a time‐dependent *group selector* $G_{a,i}\left(t\right)$ is introduced,

(2) $$G_{a,i}\left(t\right)=\left\{\begin{array}{cc} 1, & \text{if}\;i\in a(t)\\ 0, & \text{otherwise}\;. \end{array}\right.$$

We assume that the set of groups a forms a complete partition of the system, such that the completeness relation,

(3) $$\sum_{a}G_{a,i}\left(t\right)=1\;\forall i,t\;,$$

holds at all times. Inserting this identity at t=0 and t into Equation (1) allows the total VACF to be expanded as a sum of group correlations $C_{ab}\left(t\right)$,

(4) $$\begin{array}{cc} C(t) & =\sum_{a,b}C_{ab}\left(t\right)\\ & =\sum_{a,b}\frac{1}{N}\langle \sum_{i=1}^{N}G_{a,i}\left(0\right)G_{b,i}\left(t\right)\;v_{i}\left(0\right)\cdot v_{i}\left(t\right)\rangle \;. \end{array}$$

The auto‐correlation terms (a=b) represent the vibrational motion of the respective species, while the cross‐correlation terms (a≠b) capture the dynamic exchange between atoms of different groups. Note that the VDOS is a single particle quantity by definition and therefore the cross‐correlations only capture the spectral intensity caused by *a single atom* changing its chemical identity over time, which is why they have also been called “exchange” correlation functions.

The O–H stretch frequency of water is very sensitive to the local environment. A weakened intramolecular O–H bond causes a redshift (reduction in frequency), while a stronger bond leads to a blueshift (increase in frequency) [52]. Because the VDOS represents the ensemble of these frequencies, it directly reflects the distribution of local coordination environments at the interface [53]. Therefore, by resolving the spectral shifts of individual chemical species, we can map how the lithiation state modulates interfacial interactions to quantify the transition from the stable surface to active water dissociation.

To isolate these specific interfacial signatures, we restrict the analysis to H atoms residing within the first hydration layer or bound directly to the surface atoms. While all H atoms were tracked to capture the complete system dynamics, the bulk‐like liquid background—whose spectral intensity is necessarily much stronger than the interfacial signals—has been omitted and is discussed in Section S2.8.2. The interfacial H atoms were dynamically assigned to four distinct chemical species ($H_{2}O$, ${\text{OH}}^{-}$, $H_{3}O^{+}$, and $O_{\text{surf}}H$) at each time step based on a geometric distance criterion ($r_{\text{OH}}\leq 1.25$ Å).

The VDOS spectra were computed from an ensemble of 30 independent 20ps microcanonical (NVE) trajectories sampled at 1fs intervals, ensuring rigorous capture of the high‐frequency O–H stretching modes. (Signal processing details and full ensemble generation are provided in Sections S1.7 and S2.8, respectively.) Representative spectra are shown in Figure 2 (the remaining are provided in Section S2.8.3). In the fully lithiated, Li‐terminated limit ($x_{\text{Li}}=1.0$), where surface Mn ions are almost exclusively ${\text{Mn}}^{3+}$, the interfacial VDOS is characterized by a broad O–H stretching band with comparable spectral weights in the surface‐parallel (x,y) and surface‐normal (z) directions. This is indicative of a disordered, liquid‐like interfacial environment where the hydrogen bond network is not strongly modified by the surface. Consistent with the structural analysis (Figure 2a left), the species‐decomposed spectra (Figure 2a right) confirm that this signal is dominated by molecular water ($H_{2}O$), with only minor contributions from dissociation products.

Upon delithiation, as the surface oxidizes toward the ${\text{Mn}}^{4+}$ limit ($x_{\text{Li}}=0.0$) (Figure 2d), the spectral density undergoes a drastic transformation. The interfacial VDOS becomes strongly anisotropic, developing a distinct, sharp feature in the z‐direction while remaining broad in the xy‐plane. The decomposition into chemical species reveals that this evolution is driven by the emergence of dissociation species: a broad, asymmetric band associated with interfacial hydroxide (${\text{OH}}^{-}$) predominantly polarized in the surface plane, and a sharp, symmetric peak attributed to surface protons ($O_{\text{surf}}H$) oscillating perpendicular to the surface.

However, the structural evolution is not limited to the formation of new species, as the spectral signature of the remaining undissociated interfacial water molecules develops a bimodal shape with decreasing Li contents (Figure 2d). While the xy‐component remains broad, the z‐component narrows and exhibits a significant blueshift with increasing Mn oxidation state.

To resolve the atomistic origin of this shift, we further partitioned the spectral contributions of undissociated interfacial water molecules based on their orientation. Following the two coordination geometries identified above, we defined two sub‐groups: molecules pointing *toward* (cosθ<0, denoted as $H_{2}O^{t}$) or *away* (cosθ>0, denoted as $H_{2}O^{a}$) from the surface. We then applied the decomposition identity (Equation 4) to the water subset,

(5) $$C_{H_{2}O}(t)=C_{H_{2}O^{t}}(t)+C_{H_{2}O^{a}}(t)+C_{\text{exchange}}(t)\;.$$

The resulting orientation‐resolved water spectra (Figure 3) uncover a clear difference in vibrational dynamics. Water molecules coordinated to Mn sites (*away*) exhibit a systematic redshift and broadening. On the other hand, water molecules in the [110] channels, donating hydrogen bonds to the surface oxygen atoms (*toward*), show a pronounced blueshift and anisotropy with a sharp feature in the z‐direction.

To quantify the relationship between the surface electronic state and the intrinsic strength of the interfacial interactions, we analyzed the vibrational frequencies of the total VDOS, which is calculated as the sum of its x, y, and z components. While the Cartesian decomposition (Figure 3) highlights the geometric anisotropy of the interfaces, this total spectral density is required to determine the intrinsic O–H bond strength. Because these chemical bonds are not necessarily oriented along a single spatial axis, capturing the full vibrational signature requires incorporating the contributions from all three spatial dimensions. Given the significant inhomogeneous broadening and asymmetry of the interfacial spectral bands, we utilized the mean of the spectral distribution, ⟨ω⟩=∫ωI(ω)dω/∫I(ω)dω, rather than the intensity maximum [54]. Unlike the maximum peak position, which identifies only the most probable vibrational frequency, this mean accounts for the inhomogeneous broadening of the spectral weight in the tails, thereby capturing the full ensemble of local coordination environments at the interface.

To avoid convolution with the water bending mode at around 1600 ${\mathrm{cm}}^{-1}$, the mean values of the spectral distributions for all groups were evaluated consistently between 2400 and $4200\;{\mathrm{cm}}^{-1}$. Statistical uncertainties were estimated via block averaging: the 30 independent 20ps NVE trajectories were partitioned into three subsets of ten, and the standard error of the mean (including Bessel's correction) was calculated from the means of the resulting three independent spectra.

Figure 4a shows the mean frequencies of the two groups of water molecules as a function of the average surface Mn oxidation state ${\bar{q}}_{\mathrm{Mn}}$. For reference, the mean O–H stretch of bulk water ($3343\pm 2\;{\mathrm{cm}}^{-1}$; see Section S1.9) is also shown. A distinct behavior is observed for both groups of water molecules, revealing that the oxidation state has opposite effects on the two water orientations. In the reduced ${\text{Mn}}^{3+}$ state, the mean O–H stretch of $H_{2}O^{a}$ species (Mn‐coordinated) and $H_{2}O^{t}$ species (H‐bond donating) are approximately the same. With increasing Mn oxidation, the mean O–H stretch frequency of the $H_{2}O^{a}$ group decreases, confirming that the interaction is governed by the metal's Lewis acidity in that the increasingly electron‐deficient Mn centers strongly polarize the coordinated water molecules, weakening the intramolecular bond.

**FIGURE 4:**
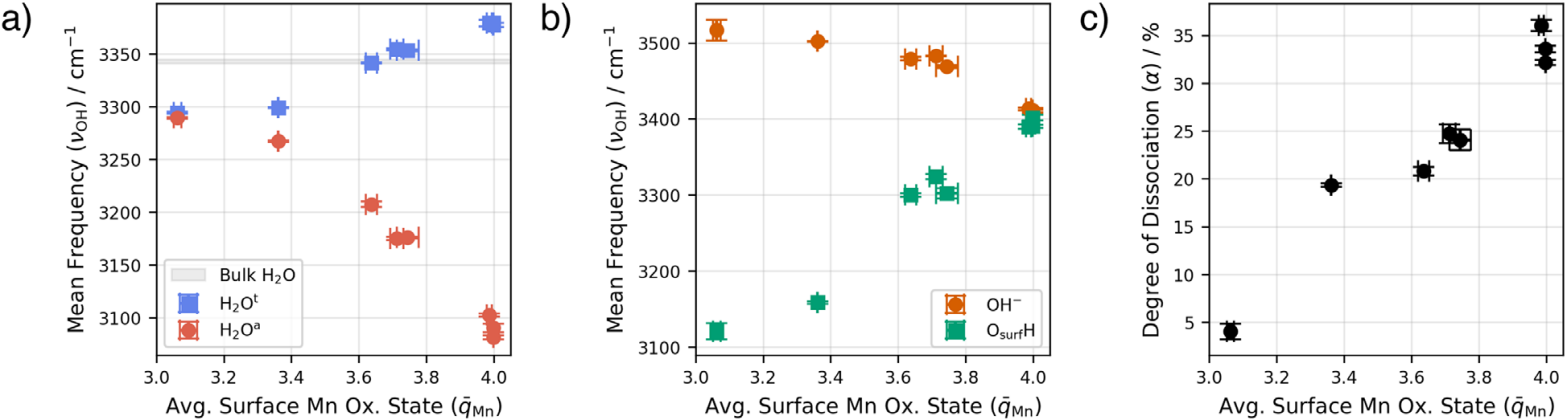
Correlation between surface acid‐base chemistry and average surface Mn oxidation state. (a) mean frequency of the OH‐stretch ($\nu_{\mathrm{OH}}$) for the first‐layer water molecules plotted against the average surface Mn charge (${\bar{q}}_{\mathrm{Mn}}$). Water molecules are classified as *toward* or *away* based on the angle between the water bisector and the surface normal vector. (b) mean OH‐stretch frequency ($\nu_{\mathrm{OH}}$) for hydroxide species as a function of ${\bar{q}}_{\mathrm{Mn}}$, distinguishing between interfacial ${\text{OH}}^{-}$ and surface‐adsorbed protons $O_{\text{surf}}H$. (c) Degree of water dissociation (α) as a function of ${\bar{q}}_{\mathrm{Mn}}$. The shaded horizontal band in (a) represents the bulk water reference ($\nu_{\mathrm{OH}}=3343\pm 2\;{\mathrm{cm}}^{-1}$; see Supporting Information).

At the same time, the $H_{2}O^{t}$ species experiences a blueshift. The structural analysis in Figure 3 places these molecules primarily in the [110] channels, donating H‐bonds to the surface oxygen atoms. At low average Mn oxidation state (${\text{Mn}}^{3+}$), these surface oxygen atoms are sufficiently basic to accept strong H‐bonds, resulting in a redshift relative to bulk water. However, as the lattice is delithiated, the depletion in electron density reduces the basicity of these surface sites, reducing their strength as H‐bond acceptors. This weakens the water–surface oxygen interaction, causing the intramolecular O–H stretch to stiffen and eventually exceed the mean bulk water frequency value by $\approx 40\;{\mathrm{cm}}^{-1}$. This reduced interaction strength explains the persistence of molecular water in the channels even in the fully delithiated state: the surface oxygen atoms in the channel are not sufficiently basic to promote dissociation.

On the other hand, the vibrational signatures of the ${\text{OH}}^{-}$ and $O_{\text{surf}}H$ species become more similar with increasing Mn oxidation state (Figure 4b), driven by the higher ligand field symmetry of the surface ${\text{MnO}}_{6}$ units in case of ${\text{Mn}}^{4+}$. Because the cooperative J–T distortion of the ${\text{Mn}}^{3+}$ sites is oriented along the surface normal (see Section S2.4), ${\text{OH}}^{-}$ and $O_{\text{surf}}H$ act as specific probes for the axial and equatorial sites, respectively. At low average Mn oxidation state, the single antibonding electron ($t_{\text{2g}}^{3}e_{g}^{1}$) of ${\text{Mn}}^{3+}$ results in a splitting of over $400\;{\mathrm{cm}}^{-1}$ between these signals. The J–T‐induced elongation of axial Mn–O bonds increases their ionic character, which reduces the polarization of the ${\text{OH}}^{-}$ oxygen by the metal center and strengthens the axial O–H bond. Simultaneously, the compression of the equatorial plane reduces local electron density at the $O_{\text{surf}}H$ sites, weakening those bonds. With increasing surface Mn oxidation, the signals start to converge, reflecting the restoration of the octahedral symmetry of the $d^{3}$ ${\text{Mn}}^{4+}$ ($t_{\text{2g}}^{3}e_{g}^{0}$) ligand field, resulting in the collapse of the spectral gap as the axial and equatorial sites become equivalent (${\bar{q}}_{\mathrm{Mn}}\approx +4.0$).

Finally, correlating these shifts with the degree of dissociation (Figure 4c) reveals that the water dissociation activity is driven by the Lewis acidity of the Mn sites. The transition to ${\text{Mn}}^{4+}$ enhances the Lewis acidity, which strongly polarizes the coordinated water molecules, lowering the barrier for proton transfer. Simultaneously, the suppression of surface oxygen basicity facilitates the mobility of the remaining molecular water in the [110] channels.

### 2.4 Thermodynamics of Water Dissociation

To connect the observed vibrational frequency shifts to the thermodynamic driving forces of water dissociation at the LMO–water interfaces, we extracted PMFs from NpT trajectories [55, 56]. By evaluating the Gibbs free energy of dissociation ($\Delta G_{\mathrm{diss}}$), we can determine how the lithiation state governs the (de‐)protonation equilibria.

We mapped the reaction thermodynamics using three coordinates: the distance along the surface normal ($r_{z}^{\mathrm{MnO}}$) between an Mn site and the nearest water oxygen; the proton displacement coordinate $\delta =r_{\mathrm{OH}}-r_{O_{\mathrm{surf}}H}$ to track O–H bond cleavage in the water molecule, where $O_{\mathrm{surf}}H$ is one of the four adjacent equatorial oxygen atoms of the active site [57]; and $r_{z}^{\mathrm{MnH}}$ between an Mn site and the active proton to monitor its transfer to the surface. The HDNNS was used to dynamically determine the active Mn oxidation state, allowing us to extract separate PMFs for the water dissociation in ${\mathrm{Mn}}^{3+}$ and ${\mathrm{Mn}}^{4+}$ environments.

PMFs were computed over extended 5 ns NpT trajectories, with configurations saved at 0.1ps intervals. Statistical uncertainties in the calculated ΔG values were estimated via block averaging: the 5ns trajectory was partitioned into three non‐overlapping ≈1.67ns blocks, and the standard error of the weighted mean (to account for fluctuations in the oxidation state distribution) was calculated from the variance across these blocks. All PMF figures were generated using the full 5 ns trajectory data.

We focus our discussion on the stoichiometric ${\mathrm{LiMn}}_{2}O_{4}(00\bar{1}$) and half‐lithiated ${\mathrm{Li}}_{0.5}^{A}{\mathrm{Mn}}_{2}O_{4}$(001) surfaces (Figure 5a–d). These interfaces exhibit intermediate reactivity and maintain substantial populations of both ${\mathrm{Mn}}^{3+}$ and ${\mathrm{Mn}}^{4+}$ centers (Table 1; ${\mathrm{Mn}}^{3+}$/${\mathrm{Mn}}^{4+}$ ratios of 1.8 and 0.3, respectively), making them representative choices to highlight lithiation‐ and oxidation‐state‐specific trends. PMFs for all other surfaces are provided in the Supporting Information (Section S2.9.2). All determined free energy differences (ΔG) are summarized in Table 2.

**TABLE 2:** Gibbs free energy differences (ΔG) for elementary steps of water dissociation at ${\mathrm{Mn}}^{4+}$ surface sites.

Surface	$\Delta G_{\mathrm{diss}}^{\mathrm{local}}$	$\Delta G_{\mathrm{rot}}^{\mathrm{local}}$	$\Delta G_{\mathrm{diss}}^{\mathrm{global}}$
${\mathrm{LiMn}}_{2}O_{4}\left(001\right)$	−88(9)	—	−130(10)
${\mathrm{LiMn}}_{2}O_{4}\left(00\bar{1}\right)$	−56(3)	—	−97(3)
${\mathrm{Li}}_{0.5}^{A}{\mathrm{Mn}}_{2}O_{4}\left(001\right)$	51(7)	−55(6)	−14(1)
${\mathrm{Li}}_{0.5}^{A}{\mathrm{Mn}}_{2}O_{4}\left(00\bar{1}\right)$	21(4)	−28(3)	−22(3)
${\mathrm{Li}}_{0.5}^{B}{\mathrm{Mn}}_{2}O_{4}\left(001\right)$	32(8)	−30(6)	−17(3)
${\mathrm{Li}}_{0.5}^{B}{\mathrm{Mn}}_{2}O_{4}\left(00\bar{1}\right)$	38(4)	−61(2)	−25(2)
$\lambda -{\mathrm{MnO}}_{2}\left(001\right)$	56(5)	−74(5)	−17(1)
$\lambda -{\mathrm{MnO}}_{2}\left(00\bar{1}\right)$	63(6)	−79(5)	−17(3)

*Note*: Values are provided for the top and bottom slab surfaces across varying Li contents. $\Delta G_{\mathrm{diss}}^{\mathrm{local}}$ and $\Delta G_{\mathrm{rot}}^{\mathrm{local}}$ were determined via PMF analysis, while $\Delta G_{\mathrm{diss}}^{\mathrm{global}}$ was derived from the ${\mathrm{OH}}^{-}$/$H_{2}O$ population ratio at ${\mathrm{Mn}}^{4+}$ sites. All values are in meV. Data were obtained from 5 ns MD trajectories in the *NpT* ensemble. Uncertainties in parentheses represent the standard error of the weighted mean, estimated via block averaging the simulation in three ≈1.67ns windows.

**FIGURE 5:**
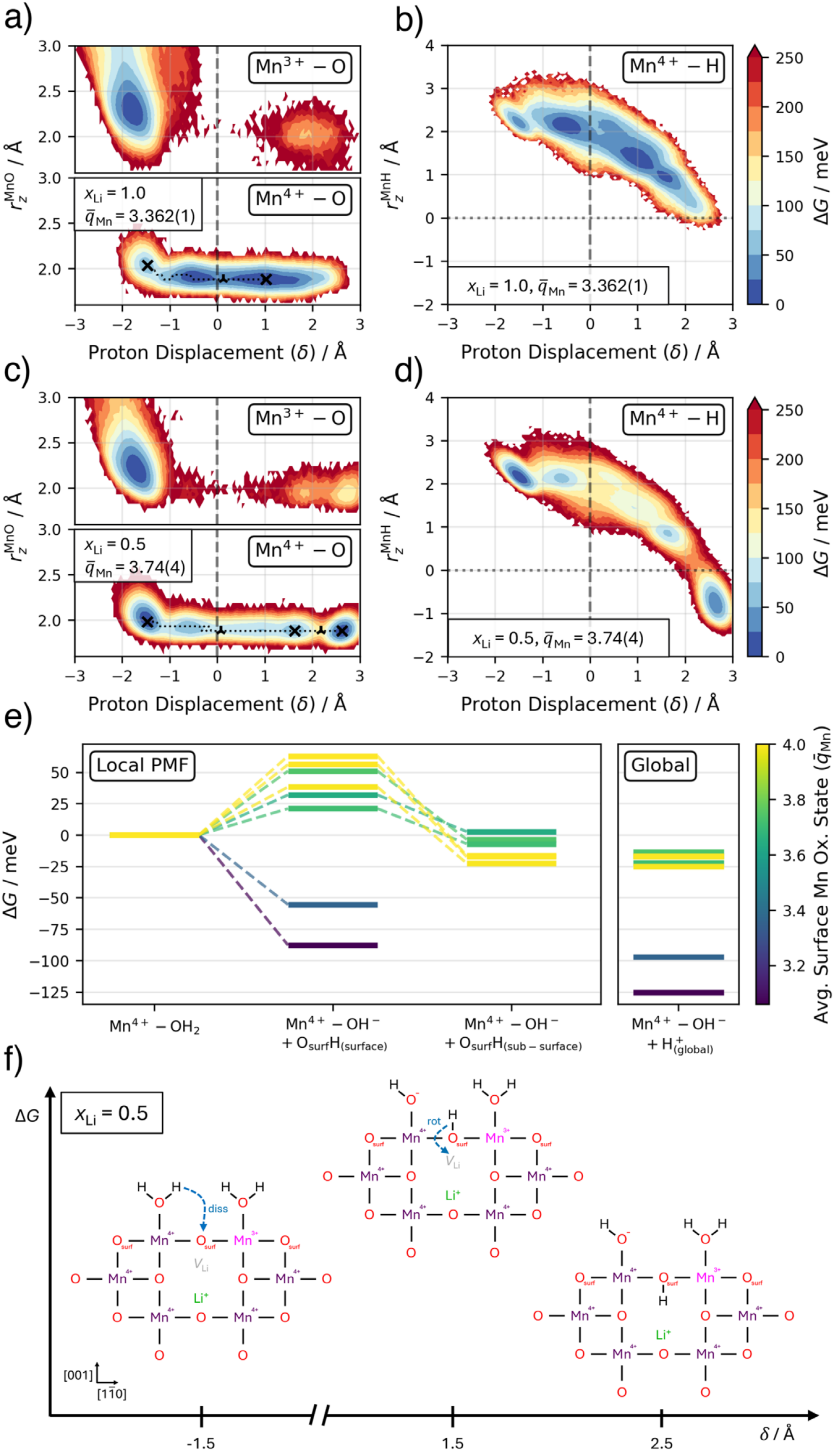
Correlation between lithiation state, average surface Mn oxidation state, and water dissociation thermodynamics. (a,b) and (c,d) present results for the stoichiometric ${\mathrm{LiMn}}_{2}O_{4}(00\bar{1}$) and half‐lithiated ${\mathrm{Li}}_{0.5}^{A}{\mathrm{Mn}}_{2}O_{4}$(001) interfaces, respectively. (a,c) Gibbs free energy surfaces (PMFs) for water dissociation at both ${\mathrm{Mn}}^{3+}$ and ${\mathrm{Mn}}^{4+}$ sites, projected onto the Mn–O distance along the surface normal ($r_{z}^{\mathrm{MnO}}$) and the proton displacement coordinate ($\delta =r_{\mathrm{OH}}-r_{O_{\mathrm{surf}}H}$) [57]. Within the ${\mathrm{Mn}}^{4+}$ sub‐panels, dotted lines trace the minimum energy path, with reactant and product minima and transition states marked by crosses and upward‐pointing triangles, respectively. (b,d) PMFs detailing the free energy landscape of the active proton at ${\mathrm{Mn}}^{4+}$ sites as a function of the Mn–H surface‐normal distance ($r_{z}^{\mathrm{MnH}}$) and δ. (e) Gibbs free energies (ΔG) for the elementary steps of water dissociation across all investigated LMO–water interfaces as a function of the average surface Mn oxidation state (${\bar{q}}_{\mathrm{Mn}}$). The hydroxyl rotation step is plotted exclusively for surfaces containing sub‐surface Li vacancies (half‐lithiated and λ‐${\mathrm{MnO}}_{2}$), which are essential for this step. To account for thermodynamic stabilization from proton delocalization—which is restricted by the local δ coordinate in the PMF calculations—a global ΔG is also presented, derived from the population ratio of adsorbed ${\mathrm{OH}}^{-}$ and $H_{2}O$ at ${\mathrm{Mn}}^{4+}$ sites. (f) Schematic structural models of the elementary dissociation steps at the half‐lithiated LMO–water interface, highlighting the thermodynamically favored rotation of the surface hydroxyl into a Li vacancy ($V_{\mathrm{Li}}$).

In the PMFs, the intact water state (δ<0) exhibits a pronounced minimum along the Mn–O $r_{z}^{\mathrm{MnO}}$ coordinate (Figure 5a,c). This minimum occurs at $r_{z}\approx 2.3$ Å for ${\mathrm{Mn}}^{3+}$ sites and $r_{z}\approx 2.0$ Å for ${\mathrm{Mn}}^{4+}$ sites, reflecting the J–T distortion of the ${\mathrm{Mn}}^{3+}$ centers perpendicular to the surface (Section S2.4) [58]. As the reaction progresses through the transition state (δ≈0) to the dissociated product (δ>0), the vertical Mn–O distance remains relatively constant, while the Mn–H $r_{z}^{\mathrm{MnH}}$ distance decreases as the proton migrates toward the surface. We emphasize that the transfer of the proton from the adsorbed water molecule to an adjacent surface oxygen site is not direct; rather, the proton migrates through a network of solvent‐mediated proton transfer reactions, which we have previously characterized for stoichiometric LMO–water interfaces [28], before ultimately settling at the surface site. Consequently, the reaction coordinate δ represents a localized projection of this network of pathways connecting the reactant and product basins.

Comparing the two Mn oxidation states in Figure 5a reveals a stark kinetic and thermodynamic disparity. At ${\mathrm{Mn}}^{4+}$ sites, proton transfer is practically barrierless and highly exergonic. Conversely, dissociation at ${\mathrm{Mn}}^{3+}$ sites is endergonic and faces an estimated barrier of >200 meV. This barrier exceeds our equilibrium sampling capabilities, effectively precluding spontaneous dissociation at these sites on the nanosecond timescale of our ambient‐condition simulations. These observations perfectly align with the vibrational analysis (Figure 4), where the $\approx 200\;{\mathrm{cm}}^{-1}$ redshift of the OH stretch of the $H_{2}O^{a}$ species on the oxidized surface indicates significant O–H bond polarization, facilitating rapid proton transfer.

Focusing on the active ${\mathrm{Mn}}^{4+}$ sites (Figure 5a,c), the dissociation thermodynamics depends heavily on the surface lithiation state. At the stoichiometric ${\mathrm{LiMn}}_{2}O_{4}(00\bar{1}$) surface, dissociation is exergonic ($\Delta G_{\mathrm{diss}}^{\mathrm{local}}=-56\pm 3$ meV). However, on the half‐lithiated ${\mathrm{Li}}_{0.5}^{A}{\mathrm{Mn}}_{2}O_{4}$(001) surface, it is endergonic ($\Delta G_{\mathrm{diss}}^{\mathrm{local}}=51\pm 7$ meV). This decreased product stability can be explained by the reduced Lewis basicity of the surface oxygen sites on the more oxidized surface. As evidenced by the blueshift of the OH stretch of the $H_{2}O^{t}$ species, these oxygen centers become weaker proton acceptors, thereby destabilizing the dissociated state.

However, at the half‐lithiated surface, the PMFs reveal a unique secondary product minimum at δ≈2.5 Å (Figure 5c,d). This state corresponds to the surface hydroxyl rotating below the surface plane, directing the proton into a vacant Li tetrahedral site (Mn–H $r_{z}<0$ Å; schematic in Figure 5f). This rotation into the electron‐rich vacancy provides substantial thermodynamic stabilization ($\Delta G_{\mathrm{rot}}^{\mathrm{local}}=-55\pm 6$ meV), which is in agreement with prior powder neutron diffraction studies identifying this as the favored orientation for lattice hydroxyls in ${\mathrm{MnO}}_{2}$ spinels [59]. Naturally, this stabilization is not possible at the stoichiometric surface due to the fully occupied Li tetrahedral sites. Crucially, this sub‐surface orientation also explains the sharp OH stretch peak of the $O_{\mathrm{surf}}H$ species observed for the delithiated interfaces (Figure 2c,d). Since these protons point inward toward the lattice oxygen, they are shielded from the dynamic hydrogen‐bond network of the interfacial water layer, resulting in a narrow, unperturbed vibrational signature.

Figure 5e summarizes these findings by plotting the reaction free energies (ΔG) of the elementary steps across all investigated LMO–water interfaces as a function of the average surface Mn oxidation state (${\bar{q}}_{\mathrm{Mn}}$). This broader comparison clearly corroborates the trends established by the stoichiometric and half‐lithiated surfaces. $\Delta G_{\mathrm{diss}}^{\mathrm{local}}$ increases with the average surface layer Mn oxidation state, reaching a maximum for the fully delithiated λ‐${\mathrm{MnO}}_{2}$ interfaces ($\Delta G_{\mathrm{diss}}^{\mathrm{local}}=63\pm 6$ meV), driven by the progressively reduced Lewis basicity of the surface oxygen sites. However, these highly oxidized surfaces also exhibit the largest thermodynamic stabilization from surface hydroxyl rotation ($\Delta G_{\mathrm{rot}}^{\mathrm{local}}=-79\pm 5$ meV). As this structural stabilization overcomes the initial endergonic dissociation step, the net reaction becomes exergonic. This thermodynamic driving force perfectly explains the high hydroxyl coverage ($\Theta_{\mathrm{OH}}\approx 0.64$) observed for these interfaces (Table 1).

Importantly, the free energy differences derived from the PMFs represent upper limits due to how the geometries are selected from the equilibrium MD simulation for the δ coordinate. First, the active proton needs to be attached to an equatorial oxygen site adjacent to the reacting Mn center, neglecting the thermodynamic stabilization provided by broader proton delocalization (configurational entropy). Second, calculating the PMFs separately for the two Mn oxidation states artificially requires the dissociated proton to stay in the immediate vicinity of the ${\mathrm{Mn}}^{4+}$ site, preventing it from migrating to a more Lewis basic ${\mathrm{Mn}}^{3+}$ environment where further stabilization is expected (Figure 4).

To quantify this missing stabilization, we calculated the global dissociation free energy,

(6) $$\Delta G_{\mathrm{diss}}^{\mathrm{global}}=-k_{B}T\ln\left(\frac{\langle N_{{\text{Mn}}^{4+}-{\text{OH}}^{-}}\rangle }{\langle N_{{\text{Mn}}^{4+}-H_{2}O}\rangle }\right)\;,$$

from the population ratio of adsorbed ${\mathrm{OH}}^{-}$ and $H_{2}O$ at ${\mathrm{Mn}}^{4+}$ sites—where $k_{B}$ is Boltzmann's constant and T=350K is the simulation temperature—effectively integrating over all protons and over the full phase space. Adsorption was defined by a geometric distance criterion ($r_{z}^{\text{MnO}}\leq 2.5\;Å$). These global values, presented in Figure 5e, are consistently more exergonic than the local PMF‐derived values ($\Delta G_{\mathrm{diss}}^{\mathrm{local}}$). Interestingly, this additional stabilization ($\Delta G_{\mathrm{diss}}^{\mathrm{global}}-\Delta G_{\mathrm{diss}}^{\mathrm{local}}$) is substantial for the stoichiometric surfaces (≈−40 meV) but decreases approximately linearly to zero as the average surface Mn oxidation state increases. We attribute this convergence to both the decreasing concentration of ${\mathrm{Mn}}^{3+}$ sites and the concurrent introduction of sub‐surface Li vacancies (detailed analysis in Section S2.10.2). For the highly oxidized, delithiated surfaces, the dominant thermodynamic stabilization mechanism is the localized rotation of the surface hydroxyl into an adjacent vacancy, which is captured by the δ coordinate. In contrast, the stoichiometric surfaces lack these vacancies, forcing the system to rely on other stabilization pathways—specifically, the long‐range proton transfer away from electron‐deficient ${\mathrm{Mn}}^{4+}$ sites toward Lewis basic ${\mathrm{Mn}}^{3+}$ environments. These proton transfer events are frequent as we have previously shown  [28] and cannot be captured by the PMF that focuses on one specific exposed Mn site and its immediate surrounding. Consequently, partially delithiated surfaces—featuring intermediate Li vacancy concentrations—leverage a mix of these different stabilization mechanisms, resulting in the observed intermediate stabilization energies.

## 3 Conclusion

To gain spectroscopic insights into interfacial acidification and instability as a function of Li content, we performed HDNNP‐driven MD simulations of LMO–water interfaces. Our results suggest that the oxidized ${\mathrm{Mn}}^{4+}$ centers act as an intrinsic source of acidity. The high Lewis acidity of ${\mathrm{Mn}}^{4+}$ promotes water dissociation, generating protons at the surface. Moreover, the vibrational analysis identifies a ${\mathrm{Mn}}^{3+}$‐rich environment as the chemically vulnerable state. The pronounced redshift of H‐bonded water molecules confirms the enhanced basicity of the lattice oxygen atoms in the reduced (${\mathrm{Mn}}^{3+}$) limit. Furthermore, the signal splitting between axial and equatorial positions of the J–T distorted ${\mathrm{MnO}}_{6}$ coordination environment—arising from the significant blueshift of the axially adsorbed hydroxide and a corresponding redshift at the protonated equatorial sites—highlights the vulnerability to electrophilic attack.

These spectroscopic insights are corroborated by our thermodynamic analysis. We demonstrate that water dissociation is kinetically favored at ${\mathrm{Mn}}^{4+}$ sites, proceeding with a significantly lower activation barrier than at ${\mathrm{Mn}}^{3+}$ centers. Thermodynamically, the generated protons are subsequently stabilized by migrating into adjacent ${\mathrm{Mn}}^{3+}$ environments or sub‐surface Li vacancies, suppressing proton recombination at the electron‐deficient ${\mathrm{Mn}}^{4+}$ active sites. Combined, these effects facilitate surface protonation as the initiating step for the acid‐promoted Hunter disproportionation and subsequent Mn dissolution [4, 7, 14, 16].

Although the bulk solvation environment in commercial organic Li‐ion battery electrolytes differs from the aqueous interface modeled here, the identified driving forces—rooted in the intrinsic electronic structure of LMO—are equally applicable to trace water impurities. Crucially, our results reveal a fundamental vulnerability of the mixed‐valence spinel surface: it simultaneously hosts the species responsible for acid generation (${\text{Mn}}^{4+}$) and those highly susceptible to acid attack (${\text{Mn}}^{3+}$). Consequently, we propose that ${\text{Mn}}^{4+}$ centers play a critical role in driving degradation, extending the conventional view beyond a purely ${\mathrm{Mn}}^{3+}$‐centric mechanism.

Hunter originally demonstrated that the disproportionation of LMO is pH‐dependent, requiring highly acidic conditions (pH<2.5) for full conversion [7]. Our observation that high Li vacancy and ${\mathrm{Mn}}^{4+}$ concentrations at high states of charge cause a pronounced increase in local interfacial acidity implies that disproportionation of the remaining surface ${\mathrm{Mn}}^{3+}$ ions and subsequent dissolution of ${\mathrm{Mn}}^{2+}$ is thermodynamically favored under these conditions, according to Le Chatelier's principle. Hunter further proposed that coupled Li‐ion diffusion and electron hopping from the bulk to the surface continuously resupplies vulnerable ${\mathrm{Mn}}^{3+}$ sites, such that dissolution can continue beyond the initial surface layer [7].

Our conclusions are based on equilibrium MD simulations at different states of charge and therefore focus on the equilibrium thermodynamics and vibrational signatures, rather than on the long‐timescale degradation processes themselves, such as ${\mathrm{Mn}}^{3+}$ disproportionation, ion migration, or structural dissolution. Directly resolving these processes would be an interesting direction for future work, for example, using enhanced sampling techniques or non‐equilibrium MD simulations. Importantly, the vibrational and thermodynamic analyses presented here consistently identify a pronounced vulnerability of the mixed‐valence surface, providing a compelling atomistic rationale for the degradation behavior observed at high states of charge [5, 10, 11, 12, 13, 14].

Finally, the vibrational fingerprints identified here—specifically the oxidation‐state‐dependent frequency splitting—provide a theoretical framework for monitoring the local state of charge and degradation mechanisms. Crucially, our computational model can be directly translated to experiment using existing techniques. By systematically tuning the OCV (and thereby the lithium content), the predicted progression of spectral features could be resolved in situ or in operando using surface‐sensitive or ‐specific spectroscopies such as SFG, ATR‐IR, or SERS [45, 60]. This opens a route to validate the proposed interfacial acidification at high states of charge. Establishing these structure–reactivity descriptors represents a significant step toward the rational design of spinel interfaces with enhanced stability and cycle life.

## Conflicts of Interest

The authors declare no conflicts of interest.

## Supporting information


**Supporting File**: anie74774‐sup‐0001‐SuppMat.pdf.

## Data Availability

The data that support the findings of this study are openly available in TUDOdata at https://doi.org/10.17877/RESOLV‐2026‐MTVHVF8D, reference number [61].
