# Supplementary material for: Insights into the Degradation Mechanism of Aqueous LiMn2O4–Cathode Interfaces by Computational Vibrational Spectroscopy
Source: Angew Chem Int Ed Engl. 2026 Sep 23;65(46):e3712910. doi: 10.1002/anie.3712910 (PMC13749282; doi:10.1002/anie.3712910)
Supplement: Supplementary file 1 [file ANIE-65-e3712910-s001.pdf]

Supporting Information

Insights into the Degradation Mechanism of Aqueous $LiMn_2O_4$-Cathode Interfaces by Computational Vibrational Spectroscopy

K. Nikolas Lausch[a, b], Redouan El Haouari[a, b], Philipp Schienbein[a, b], and Jörg Behler[a, b]

[a] *Lehrstuhl für Theoretische Chemie II, Ruhr-Universität Bochum, 44780 Bochum, Germany*
[b] *Research Center Chemical Sciences and Sustainability, Research Alliance Ruhr, 44780 Bochum, Germany*

E-mail: joerg.behler@ruhr-uni-bochum.de

Contents

S1 Computational Details 3
S1.1 Density Functional Theory Calculations . . . 3
S1.2 Tkatchenko-Scheffler Dispersion Model . . . 3
S1.3 Construction of the Reference Data Set . . . 4
S1.4 Committee High-Dimensional Neural Network Potential . . . 4
S1.4.1 n2p2 Settings . . . 15
S1.5 High-Dimensional Neural Network for Spin Prediction . . . 15
S1.5.1 RuNNer Settings . . . 17
S1.6 Molecular Dynamics Simulations . . . 17
S1.7 Velocity Autocorrelation Function . . . 18
S1.8 Effective Simulation Temperature . . . 18
S1.9 Bulk Water Vibrational Density of States . . . 19

S2 Results and Discussion 21
S2.1 Construction of Interface Simulation Boxes . . . 21
S2.2 Reliability of MD trajectories . . . 21
S2.2.1 Committee Disagreement . . . 21
S2.2.2 Charge Conservation . . . 22
S2.3 Mn Oxidation State distribution . . . 27
S2.4 Cooperative Jahn-Teller Effect . . . 29
S2.5 Remaining Time-averaged Surface Facets . . . 32
S2.6 Density Profiles . . . 32
S2.7 Concentration Profiles . . . 34
S2.8 Vibrational Density of States Decomposition . . . 36
S2.8.1 Spatial Definition of Interfacial and Bulk Regions . . . 36
S2.8.2 Interface and Bulk Background Contributions . . . 41
S2.8.3 Remaining Chemical Species Decomposed Spectra . . . 41
S2.8.4 Remaining Orientation Decomposed Spectra . . . 41
S2.9 Thermodynamics of Water Dissociation . . . 45
S2.9.1 Definition of Reaction Coordinates . . . 45
S2.9.2 Remaining Potentials of Mean Force . . . 47
S2.10 Effect of Local Li Content and Vacancies . . . 47
S2.10.1 Correlation Between Local Li Content and Surface Mn Oxidation States . . . 50

S2.10.2 Correlation Between Sub-surface Li Vacancy Concentration and Proton Stabilization . . . 52

S1 Computational Details

S1.1 Density Functional Theory Calculations

$LiMn_2O_4$ (LMO) is a strongly correlated system characterized by highly localized d-electrons. Standard Density Functional Theory (DFT) within the Generalized Gradient Approximation (GGA) suffers from a severe self-interaction error (SIE), which artificially delocalizes these states via the unphysical interaction of an electron with its own charge density. Consequently, standard GGA incorrectly predicts metallic behavior and a uniform, average low-spin $Mn^{3.5+}$ state, rather than recovering the experimental mixed-valence electronic structure consisting of distinct Mn^{4+} and high-spin (hs)-Mn^{3+} ions.[1]

To overcome these limitations, the electronic structure can be corrected using either DFT+U methods, which introduce an empirical Hubbard-like penalty term to favor d-electron localization, or hybrid functionals, which incorporate a fraction of exact Hartree-Fock (HF) exchange.[2] As demonstrated in our prior work,[1] the local hybrid functional PBE0r[3], which we use here, provides a physically accurate description of LMO. By restricting the exact HF exchange to on-site terms, PBE0r mitigates the SIE while omitting computationally expensive long-range and bond-exchange interactions. This approach successfully captures the distinct Mn^{4+} and hs-Mn^{3+} oxidation states, correctly predicting the characteristic Jahn-Teller (JT) distortion of the hs-$Mn^{3+}O_6$ octahedra.

To model aqueous LMO interfaces under varying lithiation states, the DFT reference dataset from our previous studies[1, 4, 5] was extended to include LMO–water configurations with varying lithium contents. To ensure consistency across the entire reference dataset, we utilized the exact same computational setup as established previously, employing the PBE0r functional as implemented in the Car-Parrinello Projector Augmented-Wave (CP-PAW) code (version dated September 28, 2016).[6]

Upon electronic convergence, atomic spins were evaluated to provide training data for the auxiliary High-Dimensional Neural Network for Spin prediction (HDNNS).[7] These spin states were determined by projecting the spin density onto the one-center expansions of the partial waves, utilizing atom-centered spheres with radii equal to 1.2 times the respective covalent radii.[5] Additionally, Hirshfeld partitioning[8] was performed to compute the Hirshfeld volumes required for the Tkatchenko-Scheffler (TS) dispersion correction.[9]

S1.2 Tkatchenko-Scheffler Dispersion Model

A critical challenge in simulating aqueous transition metal oxide (TMO) interfaces is that while PBE-derived[10] hybrid functionals accurately describe the solid phase, they often fail to capture the properties of liquid water.[11] Due to missing long-range dispersion interactions and over-structured hydrogen bonding, these functionals typically yield an ice-like water structure with sluggish diffusion.[12, 13] To overcome this limitation, we correct the PBE0r reference data using the Tkatchenko-Scheffler (TS) dispersion model,[9] employing our previously reported implementation.[13]

The TS model includes an empirical, exchange-correlation (xc) functional-dependent damping term to avoid the double-counting of short-range correlation.[14] Because xc functionals differ in the extent to which they describe dispersion locally, the damping function parameter s_R must be optimized for the specific functional.[9, 14, 13] Following the procedure established by Tkatchenko and Scheffler, we parameterized the Fermi-type damping function using the S22 benchmark dataset,[15] which consists of CCSD(T) interaction energies for non-covalently bound complexes. The uncorrected PBE0r functional exhibits a Mean Absolute Error (MAE) of 105.99 meV on the S22 set, underlining its only moderate description of non-covalent interactions. By optimizing the damping function parameter to $s_R = 0.98$, the MAE of PBE0r-TS is reduced to 13.92 meV, a performance comparable to the original PBE-TS parameterization ($s_R = 0.94$, MAE = 13 meV).[9]

S1.3 Construction of the Reference Data Set

The initial reference data set was obtained from our previous studies[1, 4, 5] and includes bulk water, bulk LMO, and stoichiometric LMO–water interfaces comprising different surface facets. To minimize computational cost while maximizing configuration space coverage, we employed an active learning workflow based on the Query-by-Committee (QbC) approach to extend this dataset to include LMO–water interfaces with varying Li content ($Li_{x_{Li}}Mn_2O_4$, with $x_{Li} = 1.0, 0.5,$ and 0.0).

In the first phase, we utilized the RuNNerActiveLearn tool[16, 17], which initiated numerous short MD simulations (length ≈ 100 ps) from various structures within the reference dataset at temperatures ranging from 250 to 400 K. These simulations were driven by a single preliminary HDNNP. In each cycle, the uncertainty of a configuration was determined in post-processing by re-predicting the trajectory with an ensemble of HDNNPs. New structures were selected based on an uncertainty threshold criterion that was dynamically shifted as the active learning progressed. As the sampling reached convergence, we transitioned to a strategy focused on fewer but longer trajectories (5 ns) at 350 K driven directly by a committee of eight HDNNPs[18, 19] (c-HDNNP), matching the intended production setup. In this second phase, new structures were selected based on the standard deviation of the energy and the maximum atomic force component standard deviation between the committee members.

The final dataset consists of 45,672 structures, comprising 5,127 bulk water configurations, 14,203 bulk LMO structures, and 26,342 LMO–water interface structures. The bulk LMO set spans Li contents from zero to eight ions per unit cell, while the LMO–water configurations include the $x_{Li} = 1.0, 0.5,$ and 0.0 lithiation states investigated in this work.

S1.4 Committee High-Dimensional Neural Network Potential

Machine Learning Potentials (MLPs) provide *ab initio* accuracy at a significantly reduced computational cost by learning a representation of the potential energy surface (PES) from electronic structure reference data. High-Dimensional Neural Network Potentials (HDNNPs) belong to a class of MLPs that decompose the total PES into a sum of environment-dependent atomic energy contributions using neural networks.[20, 21, 22, 23] These atomic environments are defined by a local cutoff radius R_c and characterized by Atom-Centered Symmetry Functions (ACSFs).[24] As many-body descriptors of the local coordination sphere, ACSFs are by construction invariant to the translation, rotation, and permutation of identical atoms, thereby ensuring that the predicted total energy obeys these fundamental physical symmetries.

To describe the local atomic environment of an atom i, we employ radial and angular ACSFs.[24] The radial and angular ACSFs are defined as:

$$G_i^{\mathrm{rad}} = \sum_j \mathrm{e}^{-\eta R_{ij}^2} f_{\mathrm{c}}\left(R_{ij}\right) \ , \tag{S1}$$

$$G_i^{\mathrm{ang}} = 2^{1-\zeta} \sum_j \sum_{k \neq j} \left[1 + \lambda \cos\left(\theta_{ijk}\right)\right]^{\zeta} \mathrm{e}^{-\eta\left(R_{ij}^2 + R_{ik}^2 + R_{jk}^2\right)} f_{\mathrm{c}}\left(R_{ij}\right) f_{\mathrm{c}}\left(R_{ik}\right) f_{\mathrm{c}}\left(R_{jk}\right) \ , \tag{S2}$$

where R_{ij} is the distance to neighbor j and θ_{ijk} is the angle formed with neighboring atoms j and k. The cutoff function $f_{\mathrm{c}}\left(R_{ij}\right)$ is defined as:

$$f_{\mathrm{c}}\left(R_{ij}\right) = \begin{cases} \frac{1}{2}\left[\cos\left(\frac{\pi R_{ij}}{R_{\mathrm{c}}}\right) + 1\right] & \text{for } R_{ij} \leq R_{\mathrm{c}} \\ 0 & \text{otherwise} \end{cases} . \tag{S3}$$

This function ensures that both the ACSF values and their derivatives smoothly decay to zero at the cutoff radius R_c.[24] The ACSF descriptor parameters are summarized in Table S2 and Table S3.

Table S1: Performance metrics of the individual committee members for energies (E in meV/atom) and force components (F in meV/Å) evaluated on the training and test sets. Values are presented as Root Mean Square Errors (RMSE).

Member	E_{train}	E_{test}	F_{train}	F_{test}
1	2.16	2.44	183.72	184.28
2	2.15	2.38	183.11	183.53
3	2.17	2.44	182.99	184.60
4	2.17	2.48	183.47	183.59
5	2.13	2.40	183.27	184.61
6	2.13	2.42	183.03	183.75
7	2.15	2.38	183.33	184.47
8	2.15	2.45	184.35	184.86

Table S2: Radial symmetry function parameters for the committee HDNNP and HDNNS. For all element pairs, the cutoff radius was kept constant at $R_c = 10.5\,a_0$.

Element pair	$\eta\ /\ a_0^{-2}$
H–H	0.000000, 0.007031, 0.020208, 0.049412, 0.132157
H–Li	0.000000, 0.005662, 0.015064, 0.032586, 0.070041
H–O	0.000000, 0.007489, 0.022097, 0.056480, 0.165513
H–Mn	0.000000, 0.003945, 0.009553, 0.018052, 0.031776
Li–Li	0.000000, 0.004694, 0.011838, 0.023672, 0.045116
Li–O	0.000000, 0.004718, 0.011914, 0.023868, 0.045612
Li–Mn	0.000000, 0.002186, 0.004834, 0.008119, 0.012286
O–O	0.000000, 0.005051, 0.012988, 0.026715, 0.053074
O–Mn	0.000000, 0.004457, 0.011095, 0.021785, 0.040440
Mn–Mn	0.000000, 0.001448, 0.003088, 0.004968, 0.007148

Multiple MLPs trained independently on the same reference data can be combined to form a committee. By averaging the predictions of the individual members, an improved overall accuracy for energies and forces can be obtained. Furthermore, the committee disagreement—quantified as the standard deviation of the individual HDNNP outputs—provides a measure of the generalization error, enabling uncertainty quantification of the committee's output.[18, 19]

All HDNNP committee members were trained using the neural-network potential package (n2p2) (version 2.2.0).[25, 26] They utilize the same architecture—consisting of three hidden layers with 20, 15, and 10 nodes, respectively—and the same descriptor set, but differ in their initial weights and train/test splits. Parameters were initialized following the scheme described in our previous work.[4] The specific n2p2 training settings can be found in Section S1.4.1.

The training statistics of the individual committee members are summarized in Table S1. Plots illustrating the training accuracy are shown in Figures S1 to S8.

Table S3: Angular symmetry function parameters for the committee HDNNP and HDNNS. For all triplets, the parameter $R_c = 10.5\,a_0$ was kept constant. For every listed η value, the parameters λ and ζ took the specified values $\lambda \in \{-1, 1\}$ and $\zeta \in \{1, 2, 4, 16\}$.

Central Atom	Neighbors	$\eta\ /\ a_0^{-2}$
H	H–H	0.00000, 0.04941
	H–Li	0.00000, 0.03819
	H–O	0.00000, 0.05412

Central Atom	Neighbors	η / a_0^{-2}
	H–Mn	0.00000, 0.02851
	Li–Li	0.00000, 0.02961
	Li–O	0.00000, 0.03764
	Li–Mn	0.00000, 0.01959
	O–O	0.00000, 0.04656
	O–Mn	0.00000, 0.03211
	Mn–Mn	0.00000, 0.01369
Li	H–H	0.00000, 0.03819
	H–Li	0.00000, 0.02961
	H–O	0.00000, 0.03764
	H–Mn	0.00000, 0.01959
	Li–Li	0.00000, 0.02367
	Li–O	0.00000, 0.02380
	Li–Mn	0.00000, 0.01330
	O–O	0.00000, 0.02482
	O–Mn	0.00000, 0.01792
	Mn–Mn	0.00000, 0.00707
O	H–H	0.00000, 0.05412
	H–Li	0.00000, 0.03764
	H–O	0.00000, 0.04656
	H–Mn	0.00000, 0.03211
	Li–Li	0.00000, 0.02380
	Li–O	0.00000, 0.02482
	Li–Mn	0.00000, 0.01792
	O–O	0.00000, 0.02671
	O–Mn	0.00000, 0.02343
	Mn–Mn	0.00000, 0.01618
Mn	H–H	0.00000, 0.02851
	H–Li	0.00000, 0.01959
	H–O	0.00000, 0.03211
	H–Mn	0.00000, 0.01369
	Li–Li	0.00000, 0.01330
	Li–O	0.00000, 0.01792
	Li–Mn	0.00000, 0.00707
	O–O	0.00000, 0.02343
	O–Mn	0.00000, 0.01618
	Mn–Mn	0.00000, 0.00497

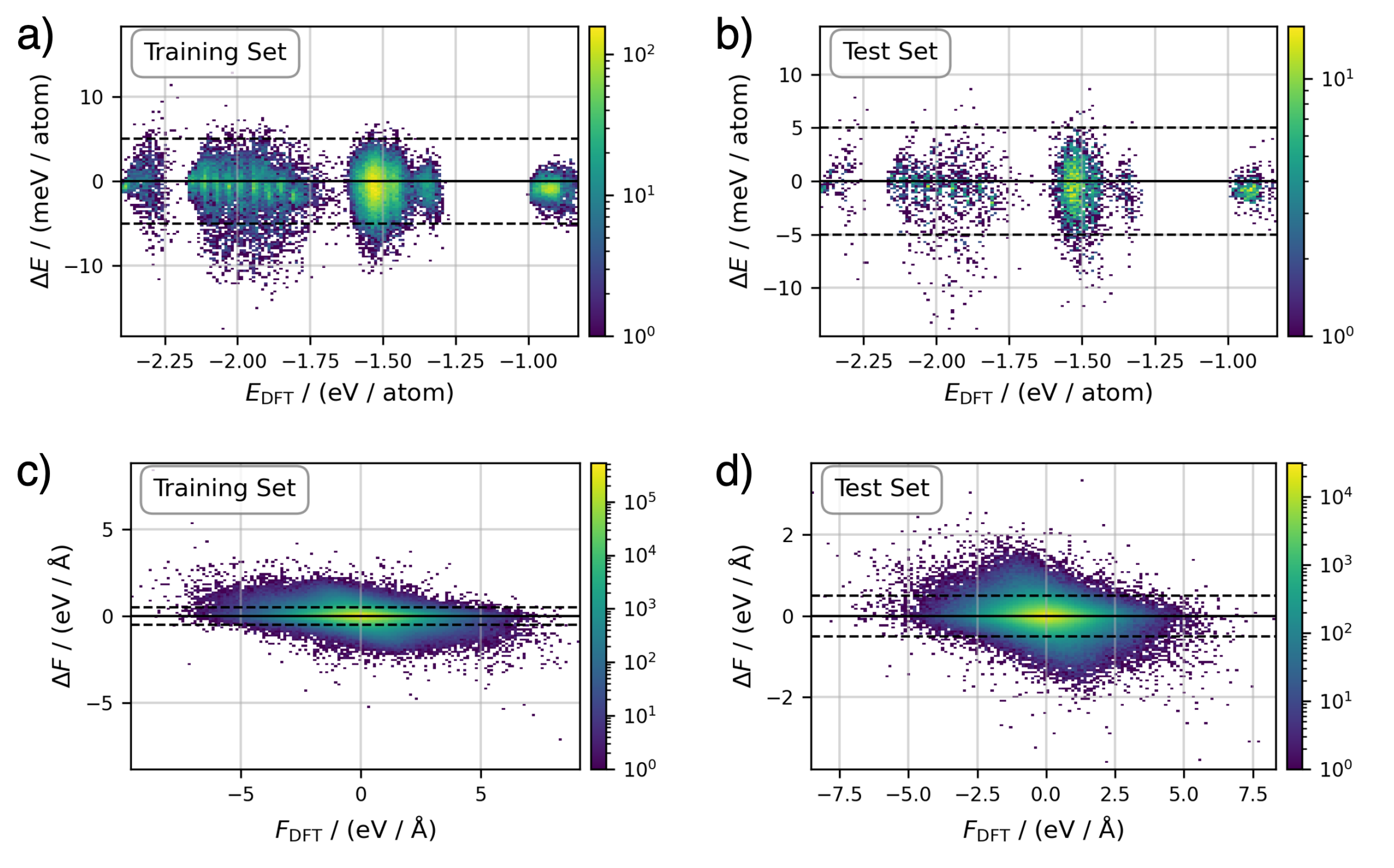

Figure S1: Accuracy of the first member of the committee-HDNNP ensemble. 2D heatmaps illustrate the energy prediction error ($\Delta E = E_{\mathrm{HDNNP}} - E_{\mathrm{DFT}}$) as a function of the DFT reference energy (E_{DFT}) for the **a)** training and **b)** test sets. Corresponding 2D heatmaps of the force component prediction error ($\Delta F = F_{\mathrm{HDNNP}} - F_{\mathrm{DFT}}$) relative to the DFT reference forces (F_{DFT}) are shown for the **c)** training and **d)** test sets. The color scale indicates the logarithmic density of the data points. To guide the eye, horizontal dashed lines denote error margins of $\pm 5\,\mathrm{meV/atom}$ for energies and $\pm 0.5\,\mathrm{eV/\AA}$ for forces.

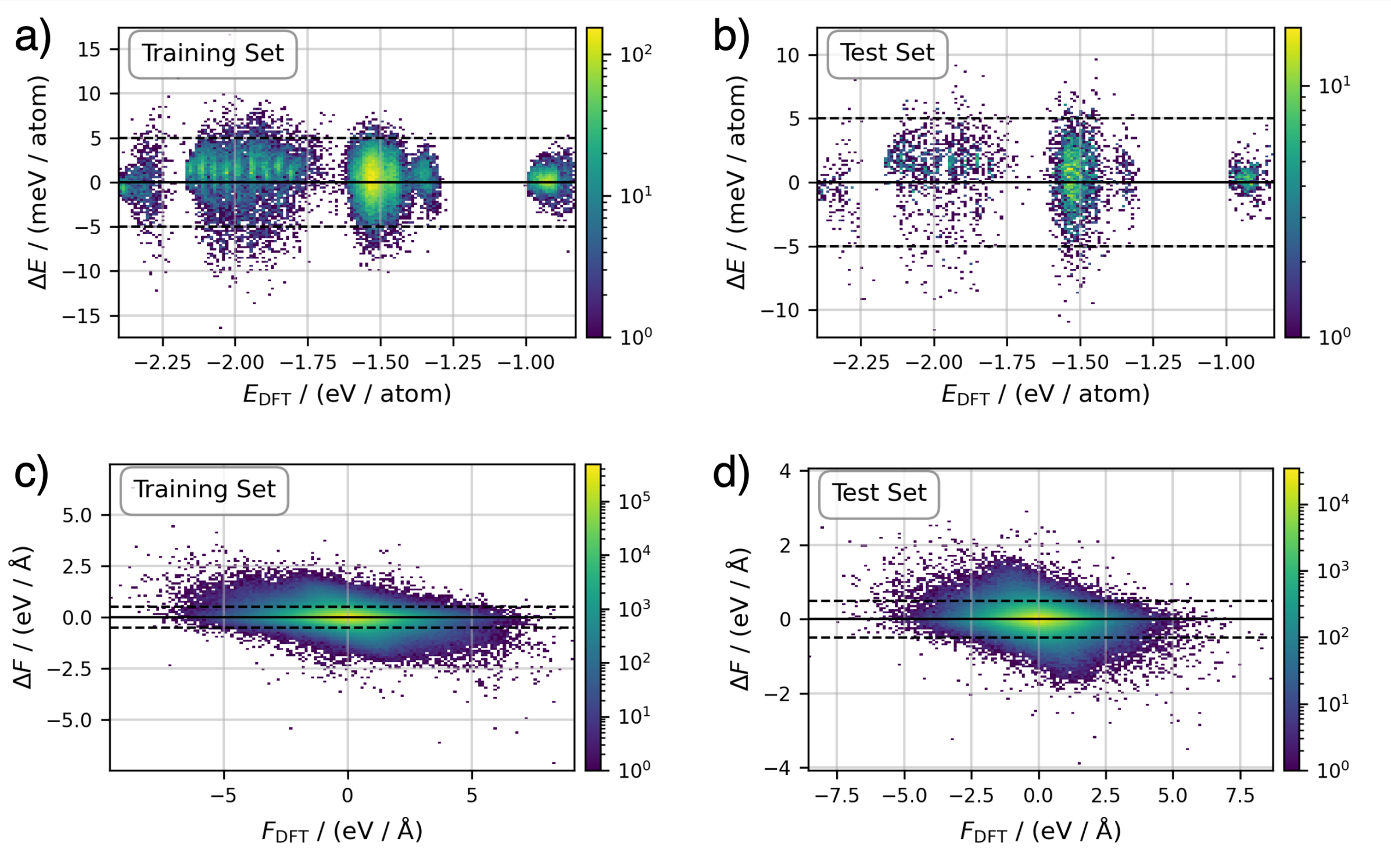

Figure S2: Accuracy of the second member of the committee-HDNNP ensemble. 2D heatmaps illustrate the energy prediction error ($\Delta E = E_{\text{HDNNP}} - E_{\text{DFT}}$) as a function of the DFT reference energy (E_{DFT}) for the **a)** training and **b)** test sets. Corresponding 2D heatmaps of the force component prediction error ($\Delta F = F_{\text{HDNNP}} - F_{\text{DFT}}$) relative to the DFT reference forces (F_{DFT}) are shown for the **c)** training and **d)** test sets. The color scale indicates the logarithmic density of the data points. To guide the eye, horizontal dashed lines denote error margins of $\pm 5\,\text{meV/atom}$ for energies and $\pm 0.5\,\text{eV/Å}$ for forces.

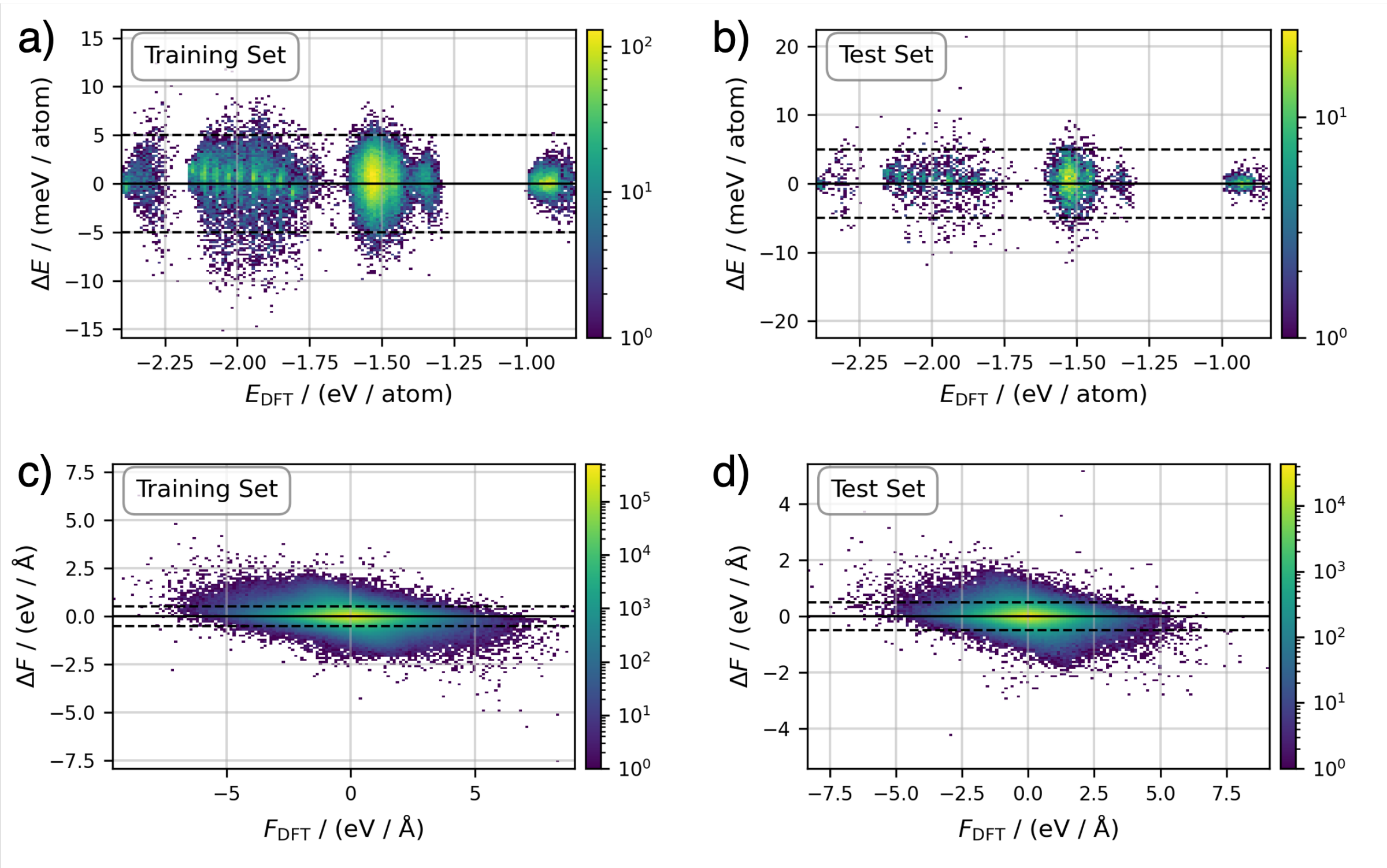

Figure S3: Accuracy of the third member of the committee-HDNNP ensemble. 2D heatmaps illustrate the energy prediction error ($\Delta E = E_{\mathrm{HDNNP}} - E_{\mathrm{DFT}}$) as a function of the DFT reference energy (E_{DFT}) for the **a)** training and **b)** test sets. Corresponding 2D heatmaps of the force component prediction error ($\Delta F = F_{\mathrm{HDNNP}} - F_{\mathrm{DFT}}$) relative to the DFT reference forces (F_{DFT}) are shown for the **c)** training and **d)** test sets. The color scale indicates the logarithmic density of the data points. To guide the eye, horizontal dashed lines denote error margins of $\pm 5\,\mathrm{meV/atom}$ for energies and $\pm 0.5\,\mathrm{eV/\AA}$ for forces.

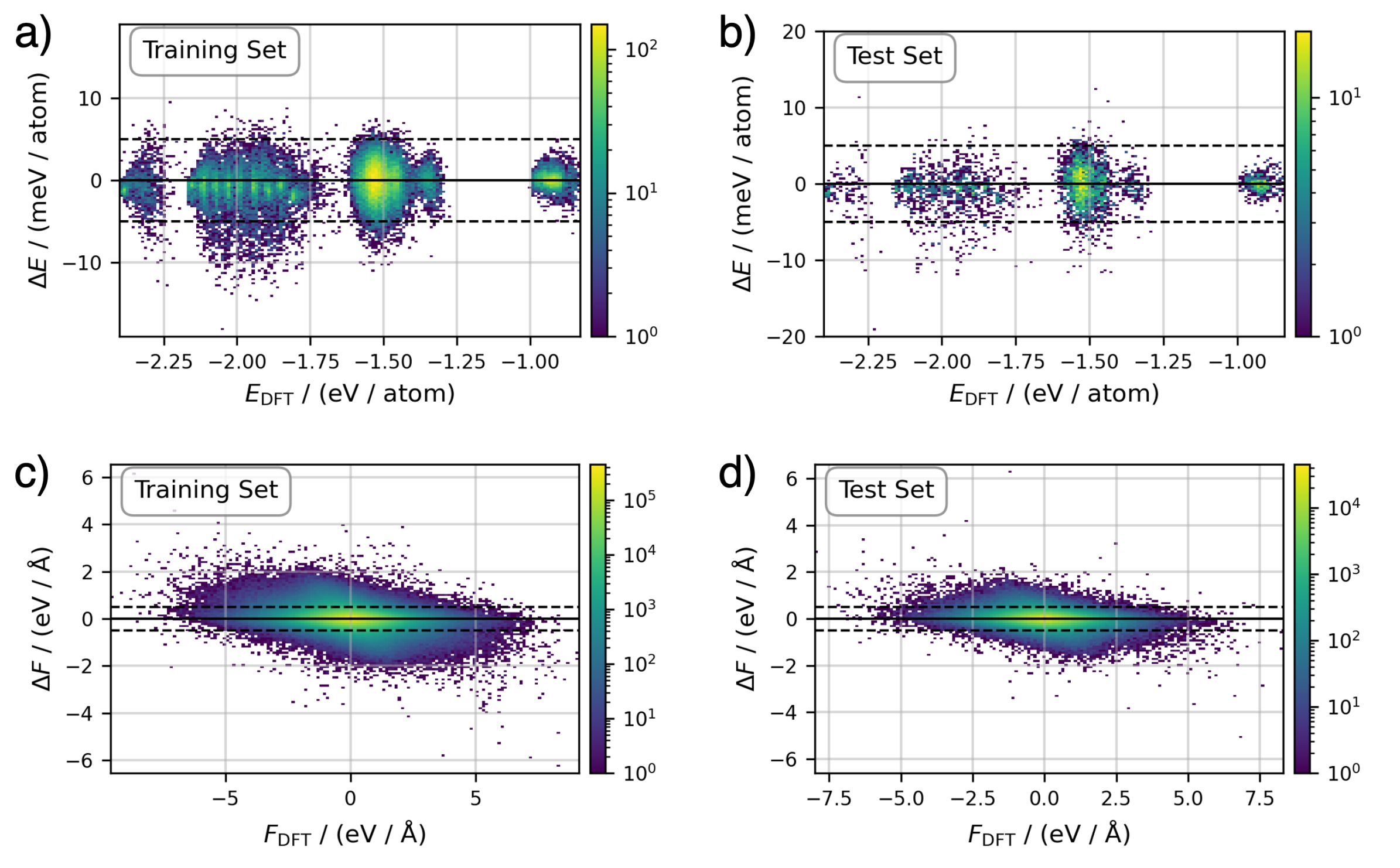

Figure S4: Accuracy of the fourth member of the committee-HDNNP ensemble. 2D heatmaps illustrate the energy prediction error ($\Delta E = E_{\mathrm{HDNNP}} - E_{\mathrm{DFT}}$) as a function of the DFT reference energy (E_{DFT}) for the **a)** training and **b)** test sets. Corresponding 2D heatmaps of the force component prediction error ($\Delta F = F_{\mathrm{HDNNP}} - F_{\mathrm{DFT}}$) relative to the DFT reference forces (F_{DFT}) are shown for the **c)** training and **d)** test sets. The color scale indicates the logarithmic density of the data points. To guide the eye, horizontal dashed lines denote error margins of $\pm 5\,\mathrm{meV/atom}$ for energies and $\pm 0.5\,\mathrm{eV/Å}$ for forces.

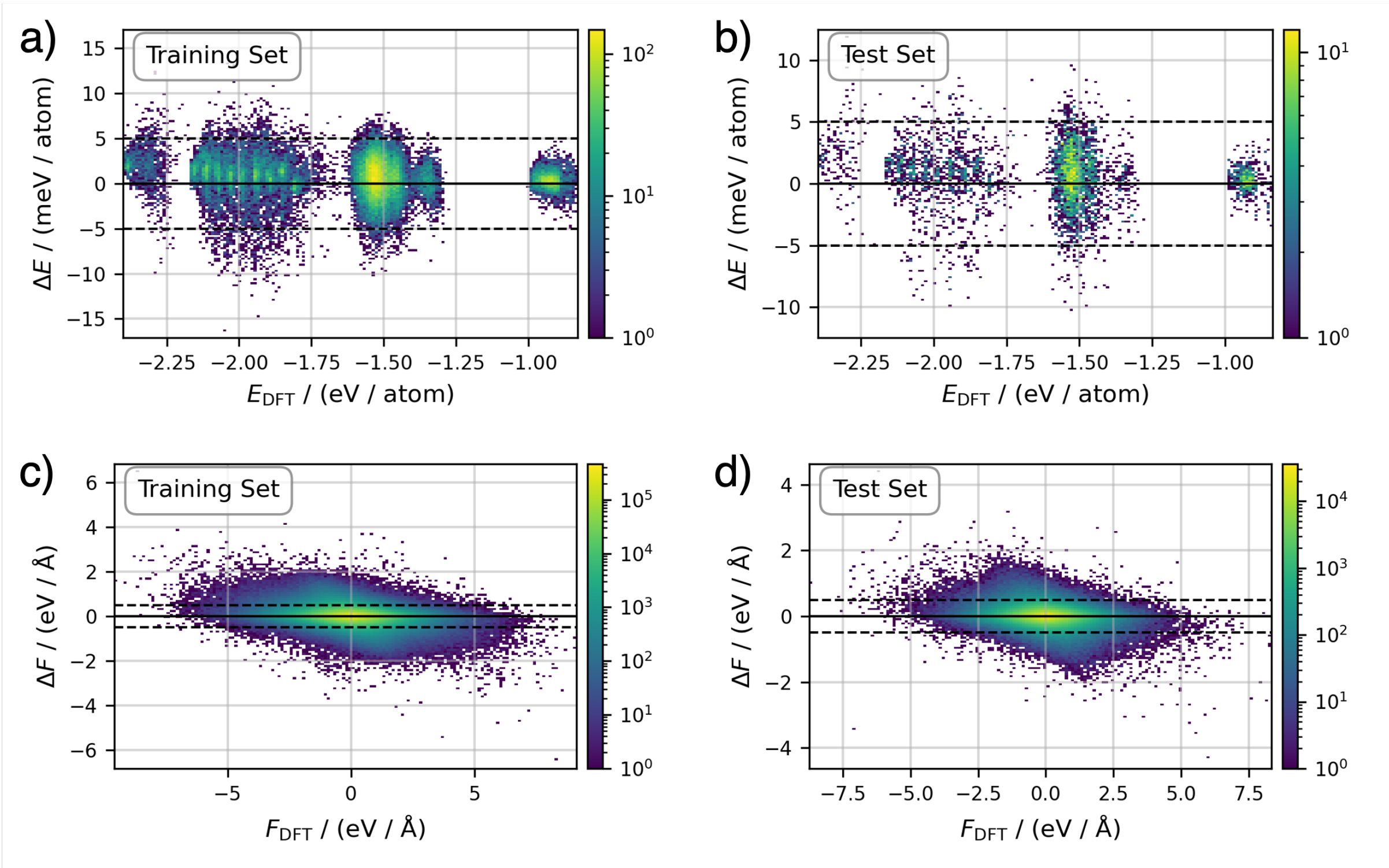

Figure S5: Accuracy of the fifth member of the committee-HDNNP ensemble. 2D heatmaps illustrate the energy prediction error ($\Delta E = E_{\mathrm{HDNNP}} - E_{\mathrm{DFT}}$) as a function of the DFT reference energy (E_{DFT}) for the **a)** training and **b)** test sets. Corresponding 2D heatmaps of the force component prediction error ($\Delta F = F_{\mathrm{HDNNP}} - F_{\mathrm{DFT}}$) relative to the DFT reference forces (F_{DFT}) are shown for the **c)** training and **d)** test sets. The color scale indicates the logarithmic density of the data points. To guide the eye, horizontal dashed lines denote error margins of $\pm 5\,\mathrm{meV/atom}$ for energies and $\pm 0.5\,\mathrm{eV/Å}$ for forces.

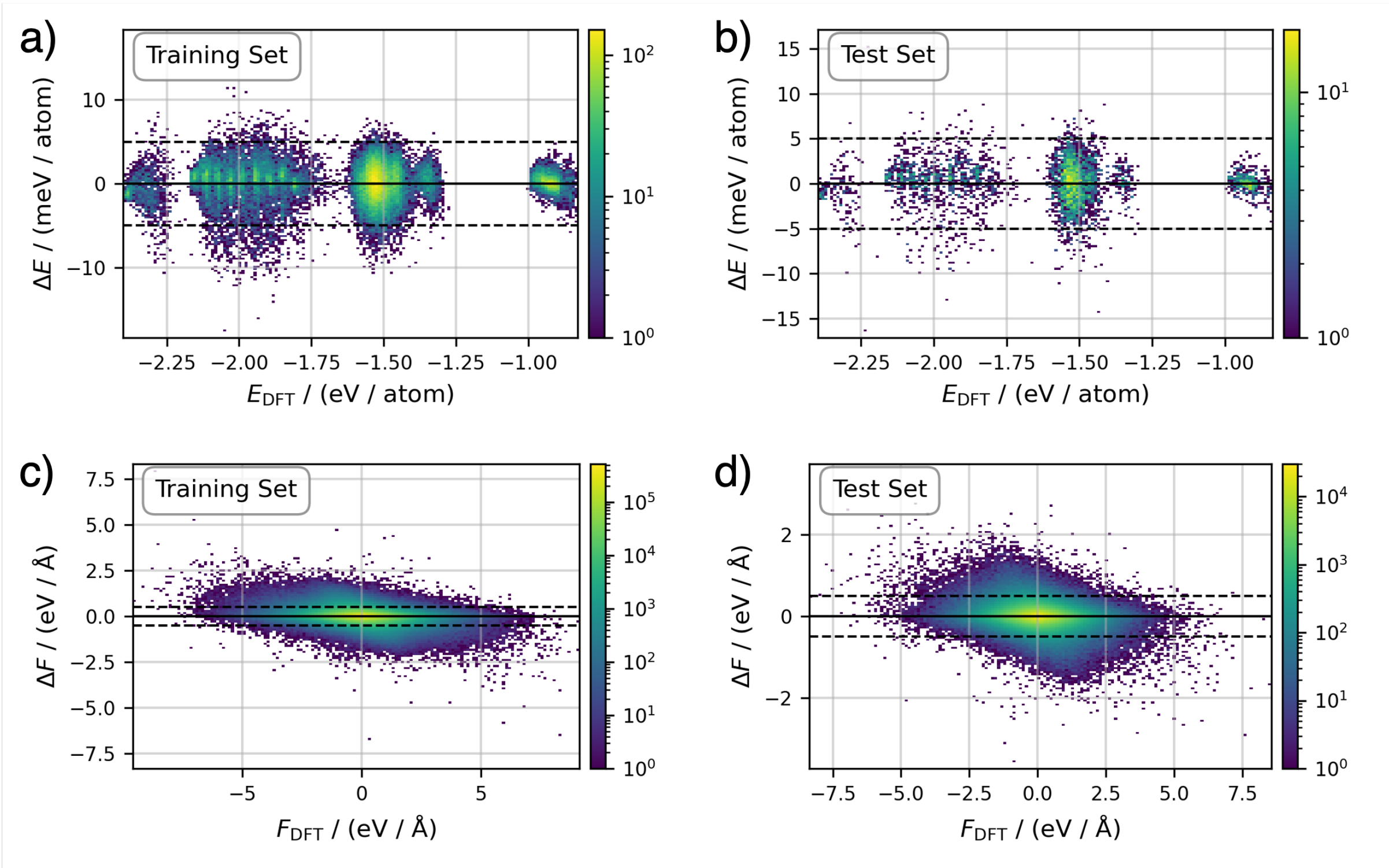

Figure S6: Accuracy of the sixth member of the committee-HDNNP ensemble. 2D heatmaps illustrate the energy prediction error ($\Delta E = E_{\mathrm{HDNNP}} - E_{\mathrm{DFT}}$) as a function of the DFT reference energy (E_{DFT}) for the **a)** training and **b)** test sets. Corresponding 2D heatmaps of the force component prediction error ($\Delta F = F_{\mathrm{HDNNP}} - F_{\mathrm{DFT}}$) relative to the DFT reference forces (F_{DFT}) are shown for the **c)** training and **d)** test sets. The color scale indicates the logarithmic density of the data points. To guide the eye, horizontal dashed lines denote error margins of $\pm 5\,\mathrm{meV/atom}$ for energies and $\pm 0.5\,\mathrm{eV/Å}$ for forces.

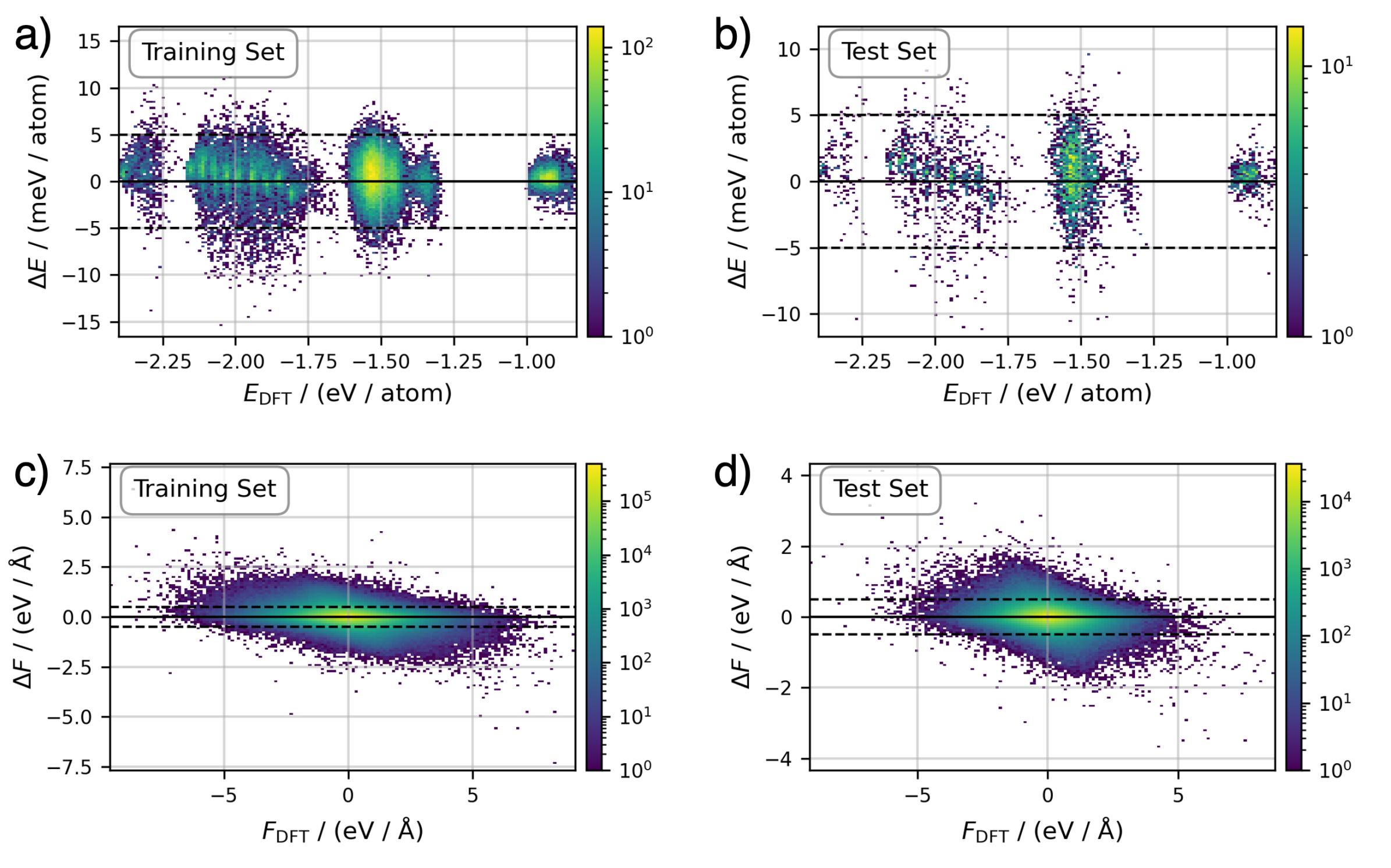

Figure S7: Accuracy of the seventh member of the committee-HDNNP ensemble. 2D heatmaps illustrate the energy prediction error ($\Delta E = E_{\mathrm{HDNNP}} - E_{\mathrm{DFT}}$) as a function of the DFT reference energy (E_{DFT}) for the **a)** training and **b)** test sets. Corresponding 2D heatmaps of the force component prediction error ($\Delta F = F_{\mathrm{HDNNP}} - F_{\mathrm{DFT}}$) relative to the DFT reference forces (F_{DFT}) are shown for the **c)** training and **d)** test sets. The color scale indicates the logarithmic density of the data points. To guide the eye, horizontal dashed lines denote error margins of $\pm 5\,\mathrm{meV/atom}$ for energies and $\pm 0.5\,\mathrm{eV/Å}$ for forces.

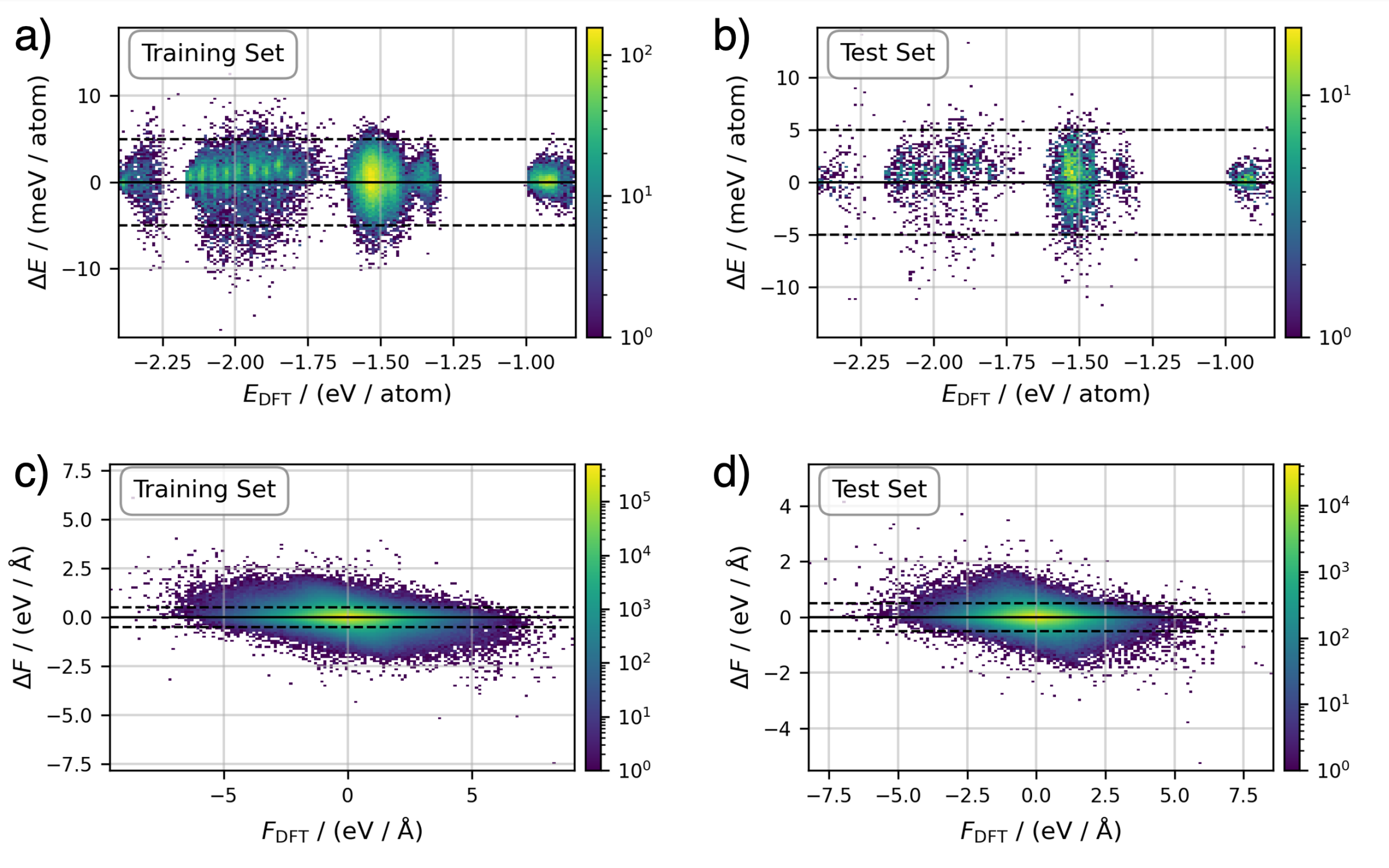

Figure S8: Accuracy of the eighth member of the committee-HDNNP ensemble. 2D heatmaps illustrate the energy prediction error ($\Delta E = E_{\mathrm{HDNNP}} - E_{\mathrm{DFT}}$) as a function of the DFT reference energy (E_{DFT}) for the **a)** training and **b)** test sets. Corresponding 2D heatmaps of the force component prediction error ($\Delta F = F_{\mathrm{HDNNP}} - F_{\mathrm{DFT}}$) relative to the DFT reference forces (F_{DFT}) are shown for the **c)** training and **d)** test sets. The color scale indicates the logarithmic density of the data points. To guide the eye, horizontal dashed lines denote error margins of $\pm 5\,\mathrm{meV/atom}$ for energies and $\pm 0.5\,\mathrm{eV/Å}$ for forces.

S1.4.1 n2p2 Settings

```
number_of_elements 4
elements H Li O Mn
global_hidden_layers_short 3
global_activation_short t t t l
global_nodes_short 20 15 10
atom_energy H -0.58437
atom_energy Li -0.27150
atom_energy O -16.09339
atom_energy Mn -104.87543
cutoff_type 1
scale_symmetry_functions
center_symmetry_functions
scale_min_short 0.0
scale_max_short 1.0
random_seed <integer>
main_error_metric RMSEpa
epochs 30
updater_type 1
parallel_mode 0
jacobian_mode 1
update_strategy 0
selection_mode 2
task_batch_size_energy 1
task_batch_size_force 1
use_short_forces
test_fraction 0.10000
force_weight 1.5
short_energy_fraction 1.000
short_force_fraction 0.0500
force_energy_ratio 1.0
short_energy_error_threshold 0.0
short_force_error_threshold 1.0
rmse_threshold_trials 3
use_old_weights_short
kalman_type 0
kalman_epsilon 1.0E-2
kalman_q0 0.01
kalman_qtau 2.302
kalman_qmin 1.0E-6
kalman_eta 0.01
kalman_etatau 2.302
kalman_etamax 1.0
write_trainpoints
write_trainforces
write_weights_epoch 1
write_neuronstats 1
write_trainlog
```

S1.5 High-Dimensional Neural Network for Spin Prediction

The High-Dimensional Neural Network for Spin prediction (HDNNS) is closely related to the HDNNP approach.[7] However, while the HDNNP decomposes a global property, such as the total potential energy, into atomic contributions, the HDNNS is trained directly on an atomic property: the absolute DFT-derived atomic spins. This local representation allows the HDNNS to identify the atomic spin state of individual atoms based on their local atomic environments.

The HDNNS was trained using the RuNNer code[21, 22] and uses the same ACSF descriptor set as the HDNNPs. However, for Li, O, and H, a reduced set consisting only radial ACSFs (see Table S2) was employed. This modification reduces computational costs without sacrificing accuracy, as the spin states of these elements are consistently zero in the reference data. The RuNNer settings are summarized in Section S1.5.1.

The HDNNS exhibits excellent accuracy, achieving training and test root-mean-squared errors (RMSEs) of just 0.02 $\hbar$ for the atomic spins. Figure S9 presents the Mn spin prediction errors ($\Delta S = S_{\mathrm{HDNNS}} - S_{\mathrm{DFT}}$) as a function of the DFT reference spin (S_{DFT}). Consistent with the low RMSE, the vast majority of spins in both sets is reproduced with negligible error. The data points are primarily clustered around $S_{\mathrm{DFT}} = 1.6$ and $S_{\mathrm{DFT}} = 2.0$, with a smaller population near $S_{\mathrm{DFT}} = 2.5$. These values correspond to the Mn^{4+}, high-spin (hs)-Mn^{3+}, and hs-Mn^{2+} oxidation states, respectively.[7, 5]

A few outliers appear in the 0.5 to 1.0 $\hbar$ range, exhibiting a larger prediction error of $\Delta S \approx 1.0\,\hbar$. An analysis reveals that these correspond to Mn ions for which the reference DFT calculations have converged to a low-spin (ls) state. For ls-Mn^{3+}, the absolute spin is 1.0 $\hbar$ (two paired and two unpaired electrons). Because the local atomic environment of these ls states are virtually indistinguishable from the hs-Mn^{3+} state, the environment-dependent HDNNS systematically predicts the more common hs value of 2.0 $\hbar$, resulting in the observed offset. However, we note that these ls-Mn species constitute a negligible fraction of the overall data set. They arise exclusively on specific surface facets that were included in the broader training data but are absent from the (001) interfaces investigated in this work. Consequently, this specific misclassification has no impact on our analysis. Indeed, as demonstrated in Section S2.2, the production simulations maintain excellent charge conservation and overall stability.

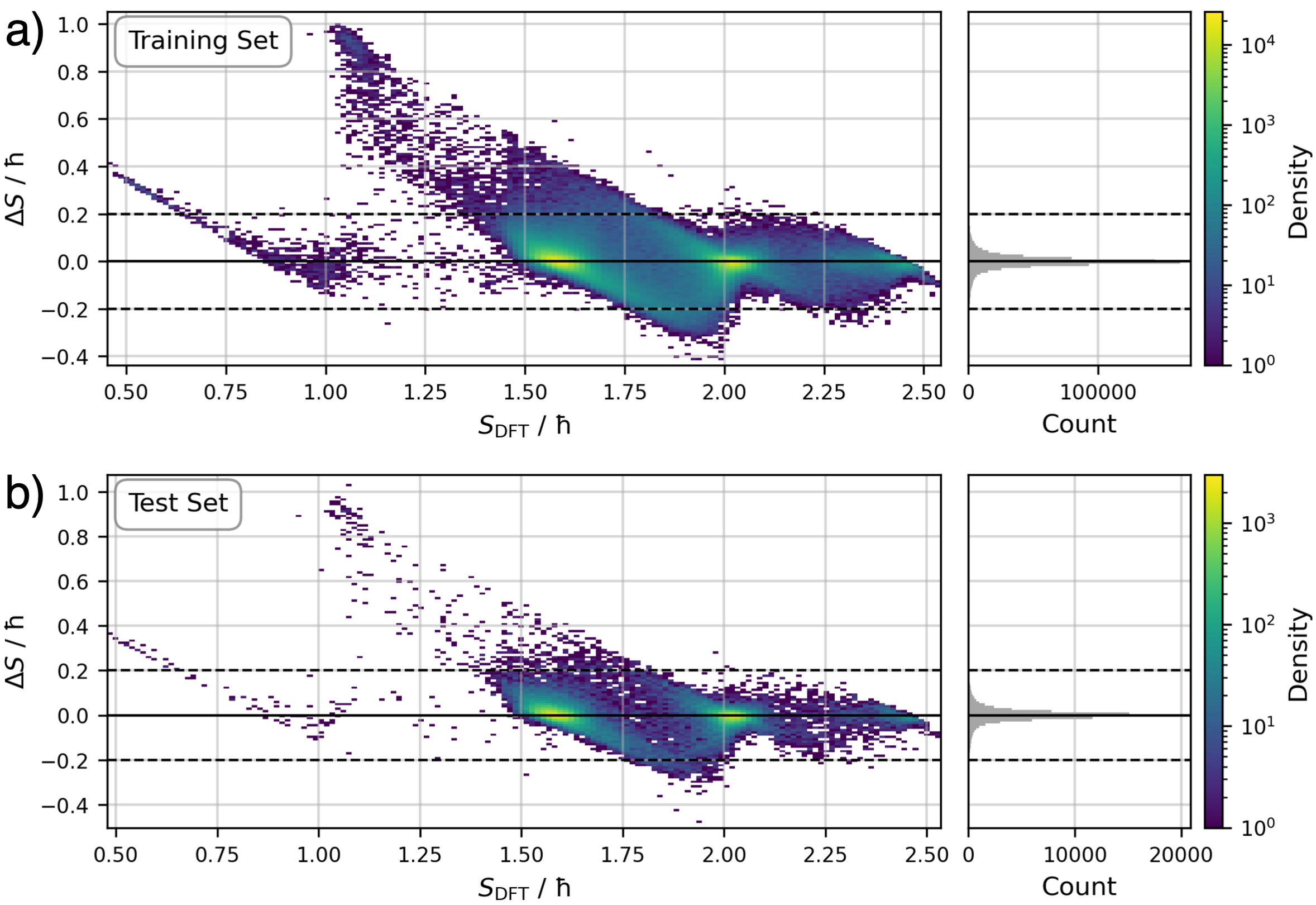

Figure S9: Accuracy of the HDNNS. 2D heatmaps illustrating the spin prediction error ($\Delta S = S_{\mathrm{HDNNS}} - S_{\mathrm{DFT}}$) for the Mn ions as a function of the DFT reference spin (S_{DFT}) for the **a)** training and **b)** test sets. The color intensity indicates the logarithmic density of data points. The right-hand panel of each subfigure displays the distribution of the prediction errors, highlighting that the vast majority of prediction errors is tightly centered around zero. To guide the eye, horizontal dashed lines denote error margins of $\pm 0.2\,\hbar$ for the spins.

Table S4: Classification criteria for assigning integer Mn oxidation states based on the absolute atomic spin S_{Mn} predicted by the HDNNS.[7, 5]

Oxidation State	Spin Range / $\hbar$
hs-Mn^{2+}	$2.2 \leq S_{\mathrm{Mn}} < 2.6$
hs-Mn^{3+}	$1.8 \leq S_{\mathrm{Mn}} < 2.2$
Mn^{4+}	$1.4 \leq S_{\mathrm{Mn}} < 1.8$
Mn^{5+}	$0.8 \leq S_{\mathrm{Mn}} < 1.4$

Following the methodology established in our previous work,[7, 5] integer oxidation states were assigned to individual Mn ions based on their absolute atomic spins S_{Mn} as predicted by the HDNNS. The classification intervals, which partition the spin density into discrete oxidation states, are summarized in Table S4. This mapping provides the necessary link between the geometry and the local electronic structure of the spinel structure to analyze its effect on the interface dynamics.

S1.5.1 RuNNer Settings

```
nn_type_short 1
runner_mode 1
parallel_mode 1
number_of_elements 4
elements H Li O Mn
random_seed 1200000002
random_number_type 5
energy_threshold 1.0
bond_threshold 1.0
use_electrostatics
electrostatic_type 1
global_hidden_layers_electrostatic 3
global_nodes_static 20 15 10
global_activation_electrostatic t t t l
ewald_alpha 1.0
ewald_kmax 0
ewald_cutoff 1.0
element_nodes_electrostatic H 1 4
element_nodes_electrostatic H 2 3
element_nodes_electrostatic H 3 2
element_nodes_electrostatic Li 1 4
element_nodes_electrostatic Li 2 3
element_nodes_electrostatic Li 3 2
element_nodes_electrostatic O 1 4
element_nodes_electrostatic O 2 3
element_nodes_electrostatic O 3 2
use_atom_spins
test_fraction 0.10000
cutoff_type 1
```

S1.6 Molecular Dynamics Simulations

All Molecular Dynamics (MD) simulations were performed using the Large-scale Atomic/Molecular Massively Parallel Simulator (LAMMPS)[27, 28] coupled with our in-house RuNNer 2.0 package, which enables the use of committee HDNNPs to drive the MD trajectories.

Interfacial structures were characterized in the isothermal-isobaric (NpT) ensemble at 350 K and 1 bar. Temperature and pressure were controlled using a Nosé-Hoover thermostat and barostat[29, 30] with coupling constants of 0.05 ps and 0.5 ps, respectively. The barostat was applied anisotropically to allow for independent relaxation of the simulation cell vectors. All trajectories were integrated with a time step of 0.5 fs for a total duration of 4 ns. The first 1 ns was discarded as equilibration, with the subsequent 3 ns used for production sampling at 0.1 ps intervals.

For the calculation of the Vibrational Density of States (VDOS), an ensemble of 30 independent microcanonical (NVE) simulations was performed for each system. To ensure representative sampling of the NpT phase space, 30 distinct replicas were generated starting from the final configuration of the 1 ns equilibration run. For each replica, atomic velocities were reinitialized from an uniform distribution at 350 K, followed by an additional 100 ps NpT equilibration period to decorrelate the starting configurations. Subsequently, NVE production runs were conducted for 20 ps, with atomic velocities sampled every 1 fs. This high temporal resolution is necessary to resolve the high-frequency O–H stretching modes (up to $\approx 4000\,\mathrm{cm}^{-1}$) while avoiding aliasing effects.

S1.7 Velocity Autocorrelation Function

For each of the NVE trajectories, we applied the Cross-Correlation Analysis (CCA) decomposition scheme [31, 32] described in the main text to partition the velocity autocorrelation function (VACF) into discrete group contributions. We performed block averaging on the individual group auto- and cross-correlation functions: the 30 independent 20 ps NVE trajectories were divided into three subsets of ten. Within each subset, the correlation functions were averaged, damped with a half-Hann window (decaying from one to zero within 20 ps) to minimize spectral leakage, and converted to the frequency domain via a Discrete Cosine Transform (DCT).

Subsequent spectral analyses were performed on each subset's spectra individually; this approach enabled us to use the standard error of the mean for the mean frequencies of the OH-stretch (incorporating Bessel's correction) as the error metric for each respective group contribution. The final spectra presented in the main text and supporting information represent the grand average of these three subsets (i.e., encompassing all 30 trajectories) to minimize statistical noise.

S1.8 Effective Simulation Temperature

A critical challenge in simulating aqueous transition metal oxide (TMO) interfaces is that while PBE-derived[10] hybrid functionals (i.e., PBE0r[3]) accurately describe the solid phase, they often fail to capture the properties of liquid water.[12, 11, 13] This deficiency arises from missing long-range dispersion interactions and over-structured hydrogen bonding, which typically results in an ice-like water structure with sluggish diffusion.[12, 13] While we apply the TS dispersion correction to remedy the missing dispersion (see Section S1.2), previous work has shown that for PBE-based simulations, the addition of dispersion corrections alone is often insufficient to reproduce ambient water properties.[13] A common strategy to resolve this is to utilize an elevated simulation temperature, calibrated such that the structural and dynamical properties of water at ambient conditions are accurately recovered.[33, 34, 35, 13]

The simulation temperature was calibrated using benchmark data from Daru et al.[36], who reported radial distribution functions (RDFs) of liquid water at 298.15 K obtained from coupled-cluster molecular dynamics (CCMD). These benchmarks show excellent agreement with experimental neutron and X-ray diffraction results. Additionally, we utilized the self-diffusion coefficient of liquid water at 298.15 K reported by Stolte et al.[37], also derived from CCMD simulations.

To calculate the RDFs, MD simulations were performed in the NVT ensemble using a cubic periodic cell (box length $L = 24.83$ Å) containing 512 water molecules, corresponding to a density of $1.0\,\mathrm{g/cm^3}$. Trajectories were integrated for 2.1 ns with a 0.5 fs time step. The first 100 ps were discarded as equilibration, and the RDFs were subsequently averaged over the remaining 2 ns using snapshots sampled every 5 fs.

Self-diffusion coefficients (D) were determined from NVE simulations using a 0.5 fs time step for 100 ps. To account for finite-size effects and extrapolate D to the infinite-system limit, simulations were performed for systems containing 64, 128, 216, and 512 water molecules. For each system size, starting configurations were sampled from 100 ps NVT runs at a density of $1.0\,\mathrm{g/cm^3}$. Ten independent replicas were generated per size to ensure sufficient sampling of the canonical phase space. The full workflow for the diffusion coefficient determination follows the methodology detailed in our previous work.[13]

Finally, the equilibrium density of bulk water was determined from a 2 ns NpT simulation of 512 water molecules, allowing for isotropic cell fluctuations. The first 1 ns was discarded for equilibration, while the subsequent 1 ns was used to sample the system density at 1 ps intervals.

We found good agreement with the CCMD reference data at an elevated simulation temperature of 350 K, as illustrated in Figure S10. The O–O RDF (Figure S10a) closely matches the CCMD benchmark. While a slight increase in structuring is evident from the higher first peak and deeper

first minimum, the overall structure is significantly improved and appears much "softer" than the PBE-TS results obtained at 300 K in our previous work[13]. A similar trend is observed for the O–H RDF in Figure S10b. Compared to the O–O RDF, the O–H distribution exhibits more pronounced structuring relative to the CCMD reference. This discrepancy can be attributed to the neglect of nuclear quantum effects (NQEs) in our classical MD simulations; previous studies have shown that NQEs affect the O–H RDF more significantly than the O–O RDF.[38, 39, 40]

The self-diffusion coefficient extrapolated to the infinite-system limit, D_∞, for PBE0r-TS at 350 K is $0.28 \pm 0.06\,\text{Å}^2/\text{ps}$ (Figure S10c). This value is slightly higher than, but in reasonable agreement with, the CCMD benchmark of $0.234 \pm 0.009\,\text{Å}^2/\text{ps}$ at 298.15 K. While a slight reduction in the simulation temperature might improve the agreement for the mean diffusion coefficient, the RDFs already exhibit slightly enhanced structuring compared to the CCMD reference; a further temperature reduction would exacerbate this over-structuring. Finally, the equilibrium density obtained at 350 K is $1.01\,\text{g/cm}^3$ (Figure S10d), which is in excellent agreement with the experimental value of $0.997\,\text{g/cm}^3$.[41]

For LMO, no high-fidelity electronic structure reference data is available for a similar calibration. However, LMO undergoes a distinct orthorhombic-to-cubic phase transition at 290 K.[42] In our previous work,[4] we found that PBE0r overestimates this transition temperature by approximately 60 K, with the transition occurring between 350 K and 380 K. Consequently, the elevated simulation temperature of 350 K—which significantly improves the description of liquid water—should similarly improve the description of LMO by bringing its structure dynamics closer to those expected at ambient conditions.

S1.9 Bulk Water Vibrational Density of States

To establish a bulk reference spectrum, we performed 20 independent MD simulations of 512 water molecules. The initial configuration was sampled after a 1 ns NpT equilibration at 350 K and 1 bar, allowing for isotropic cell fluctuations. To ensure statistically decorrelated starting states, the atomic velocities for each MD trajectory were reinitialized from a uniform distribution at 350 K, followed by a 100 ps NpT equilibration period. Finally, NVE production runs were conducted for 20 ps, with atomic velocities sampled every 1 fs.

For each trajectory, the H-atom velocity autocorrelation function (VACF) was calculated. We applied a block-averaging scheme to the individual autocorrelation functions by dividing the 20 independent trajectories into four subsets of five. As detailed in Section S1.7, the correlation functions were averaged within each subset, damped with a half-Hann window (decaying from one to zero within 20 ps) to minimize spectral leakage, and converted to the frequency domain via a Discrete Cosine Transform (DCT).

Spectral peak and mean analyses were performed on each subset's spectrum individually. This approach enabled the use of the standard error of the mean for the resulting frequencies (incorporating Bessel's correction) as an error metric. The final bulk water spectrum presented in Figure S11 represents the grand average of all four subsets (encompassing all 20 trajectories) to maximize the signal-to-noise ratio.

To validate the simulated spectra, we determined the peak positions and mean frequency for the H–O–H bending and O–H stretching modes of bulk water. The bending mode is centered at $1609 \pm 1\,\text{cm}^{-1}$, while the stretching mode features a peak at $3441 \pm 5\,\text{cm}^{-1}$ with a corresponding mean frequency at $3443 \pm 2\,\text{cm}^{-1}$.

These values are in good agreement with experimental data, where the bending mode is typically located at $1640\,\text{cm}^{-1}$ and the stretching region is centered at $\approx 3400\,\text{cm}^{-1}$.[43]

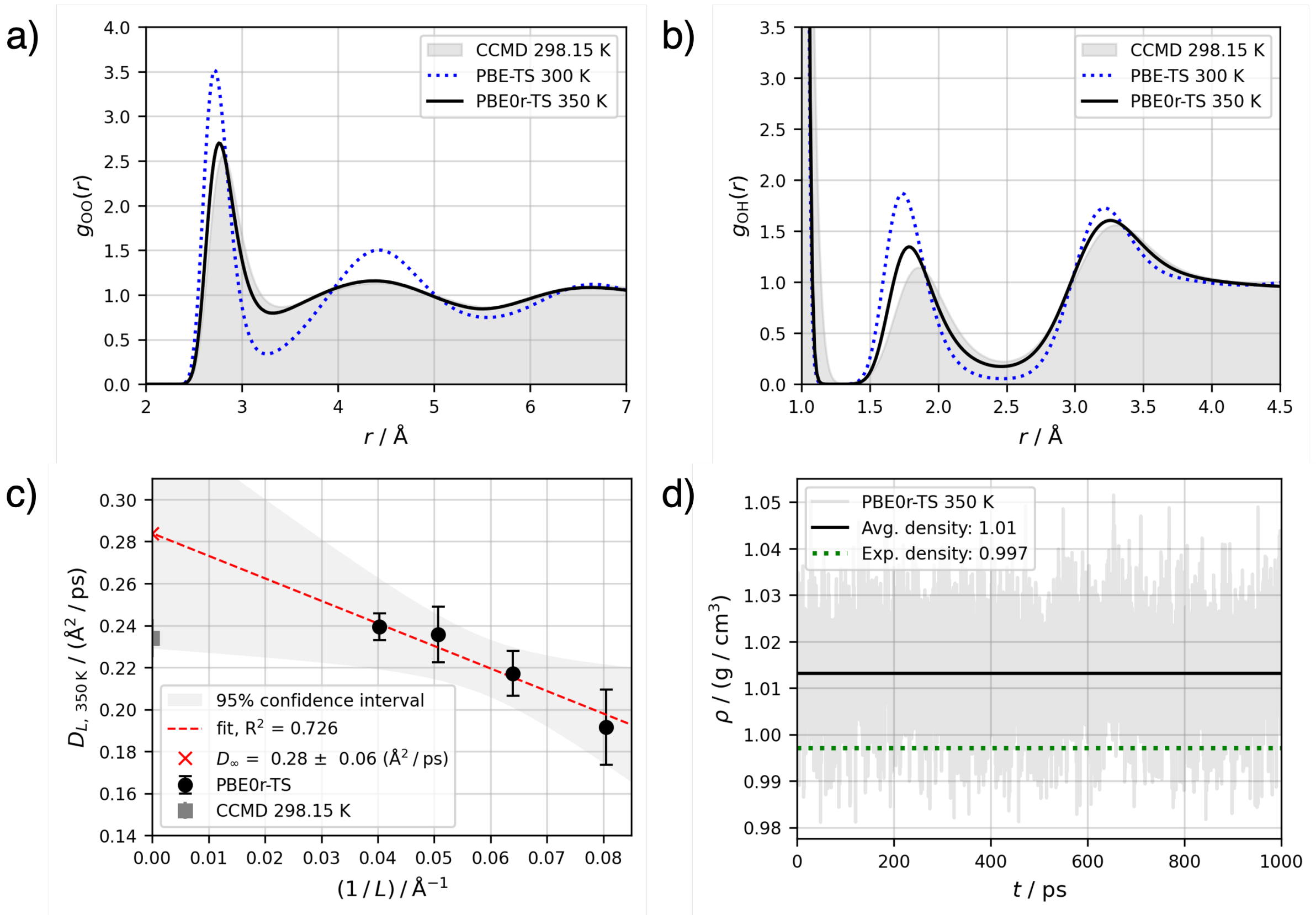

Figure S10: Calibration of the simulation temperature for liquid water using the PBE0r-TS committee HDNNP. **a)** Oxygen-oxygen (O–O) and **b)** oxygen-hydrogen (O–H) radial distribution functions (RDFs) computed at 350 K, compared against coupled-cluster molecular dynamics (CCMD) benchmark data at 298.15 K[36] and PBE-TS data at 300 K.[13] **c)** Extrapolation of the self-diffusion coefficient to the infinite-system limit (D_∞) at 350 K, alongside the CCMD reference value.[37] **d)** Equilibrium density of bulk water sampled over a 1 ns NpT trajectory at 350 K, with the experimental value at ambient conditions (0.997 g/cm^3)[41] indicated for comparison.

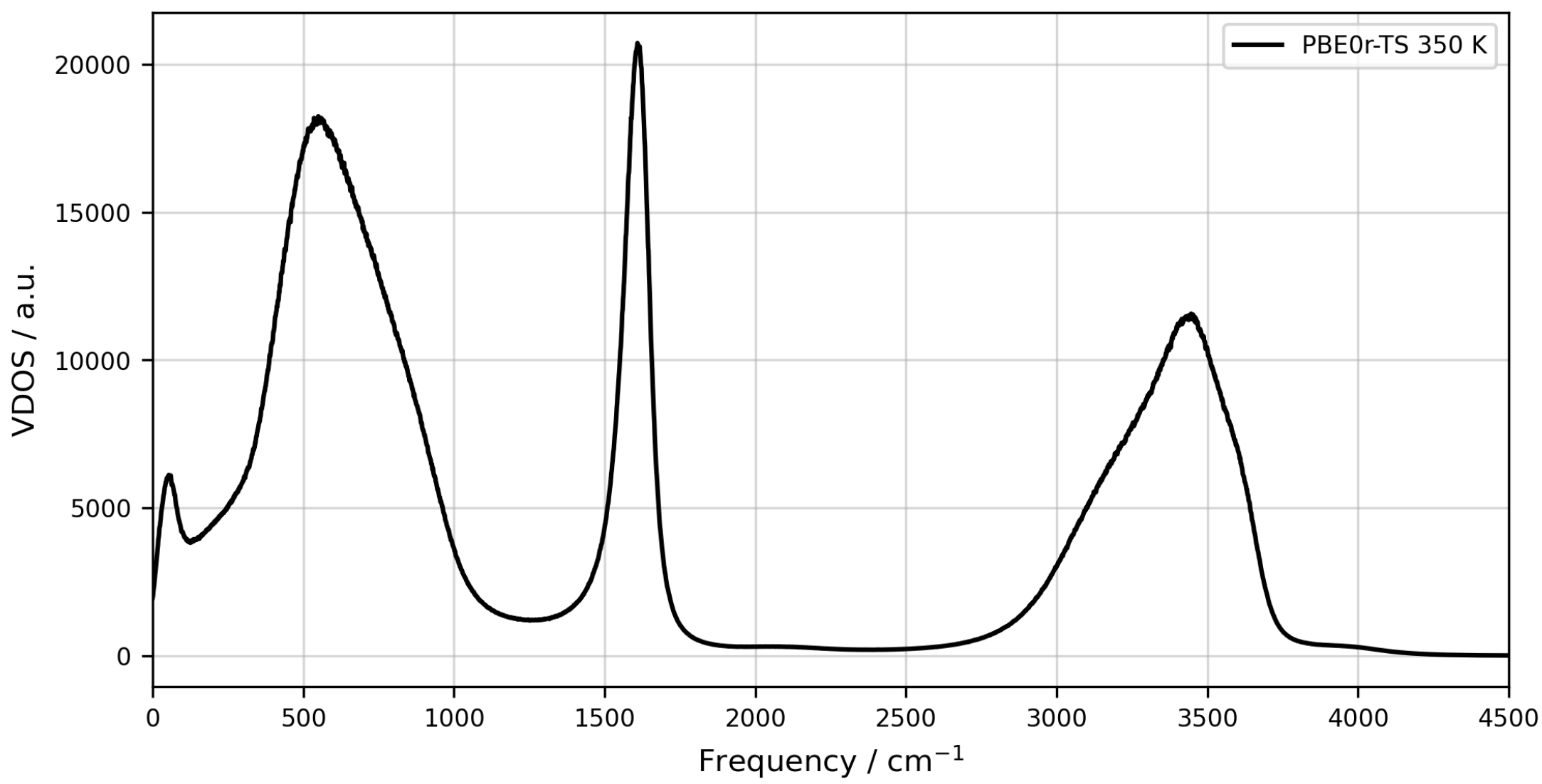

Figure S11: Vibrational density of states (VDOS) of bulk liquid water at an elevated temperature of 350 K, which recovers the dynamics of water under ambient conditions.

S2 Results and Discussion

S2.1 Construction of Interface Simulation Boxes

We represented the aqueous $LiMn_2O_4$ (001) interfaces using 4×4 supercells providing a surface area of $\approx 10\,nm^2$. These supercells comprise 40 atomic layers in total, featuring an alternating Li and MnO_2 layer sequence that yields a slab thickness of $\approx 40\,Å$. The resulting slabs were placed in contact with water films of 40 Å thickness containing 1500 water molecules. The initial oxide slab was constructed using a Crystallographic Information File (CIF) derived from experimental XRD data.[42] However, to ensure consistency with our underlying level of theory, this starting structure was subsequently fully relaxed to yield the equilibrium atomic positions and lattice vectors of our reference method.

The spinel (001) surface is a Tasker Type III polar interface, which conventionally requires reconstruction to quench the net dipole moment of the slab.[44] However, DFT studies by Kim et al.[45] demonstrated that simple cleaved slabs (T1) of LMO yield consistent surface energies compared to reconstructed models containing vacancies, steps due to ion relocation (T2), or additional non-stoichiometric layers (T3), provided the slab geometry is allowed to relax. Guided by these findings, we retained the as-cleaved T1 termination while allowing flexible changes of the surface during the simulations. This approach preserves the exact stoichiometry and ideal planar topology of the (001) facet. Unlike reconstruction schemes that inherently alter the slab composition or introduce artificial steps, our model avoids creating extrinsic defect sites that would obscure the vibrational signatures of the intrinsic surface chemistry. Furthermore, it mimics the relevant metastable state during electrochemical cycling, where transient charge imbalances are stabilized by the material's mixed-valence electronic buffer (Mn^{3+}/Mn^{4+} ratio) rather than immediate ionic rearrangement.[46]

To model different charge states, we systematically removed Li ions and rescaled the initial lattice parameters according to experimental data[47, 48] to minimize stress and facilitate equilibration in the NpT ensemble. For the intermediate concentration ($x_{\mathrm{Li}} = 0.5$), Li ions were arranged to exclusively populate one of the two interpenetrating face-centered cubic (*fcc*) sub-lattices of the diamond-like *8a* sites,[46] capturing the Li-Li repulsion-driven ordering effects observed in previous work.[4] Given that the Li diffusion barrier is $\approx 0.5\,eV$,[49, 4] ion migration is rare at 350 K on the nanosecond timescales accessible in HDNNP-driven MD, preventing spontaneous global equilibration. Consequently, two independent configurations were generated to sample the symmetry breaking associated with each sub-lattice ($Li^{A}_{0.5}Mn_2O_4$ and $Li^{B}_{0.5}Mn_2O_4$). Finally, complete delithiation yielded the λ-Mn_2O_4 structure ($x_{\mathrm{Li}} = 0.0$). This slab presents symmetric Tasker Type II facets with no net dipole, rendering the as-cleaved surface inherently stable without the need for further intrinsic or extrinsic compensation. The resulting simulation cells contained 8980 ($x_{\mathrm{Li}} = 1.0$), 8660 ($x_{\mathrm{Li}} = 0.5$), and 8340 ($x_{\mathrm{Li}} = 0.0$) atoms, respectively, and are shown in Figure S12.

S2.2 Reliability of MD trajectories

S2.2.1 Committee Disagreement

To monitor the reliability of the MD simulations, we evaluated the committee disagreement for both the predicted energies and atomic forces. For the energy, we calculated the standard deviation of the predicted total energy, σ_E, for each configuration. For the atomic forces, we determined both the mean standard deviation across all atomic force components, $\sigma_{F,\mathrm{mean}}$, and the maximum standard deviation of any single force component, $\sigma_{F,\mathrm{max}}$, within a given configuration.

Figures S13 to S16 illustrate the evolution of the committee disagreement throughout the production trajectories. The consistently low values of $\sigma_E \approx 0.5\,meV/atom$ and $\sigma_{F,\mathrm{mean}} \approx 35\,meV/Å$ demonstrate the high reliability of the committee HDNNP within the sampled configuration space. Furthermore, the maximum force standard deviations ($\sigma_{F,\mathrm{max}} \approx 210\,meV/Å$) fall well

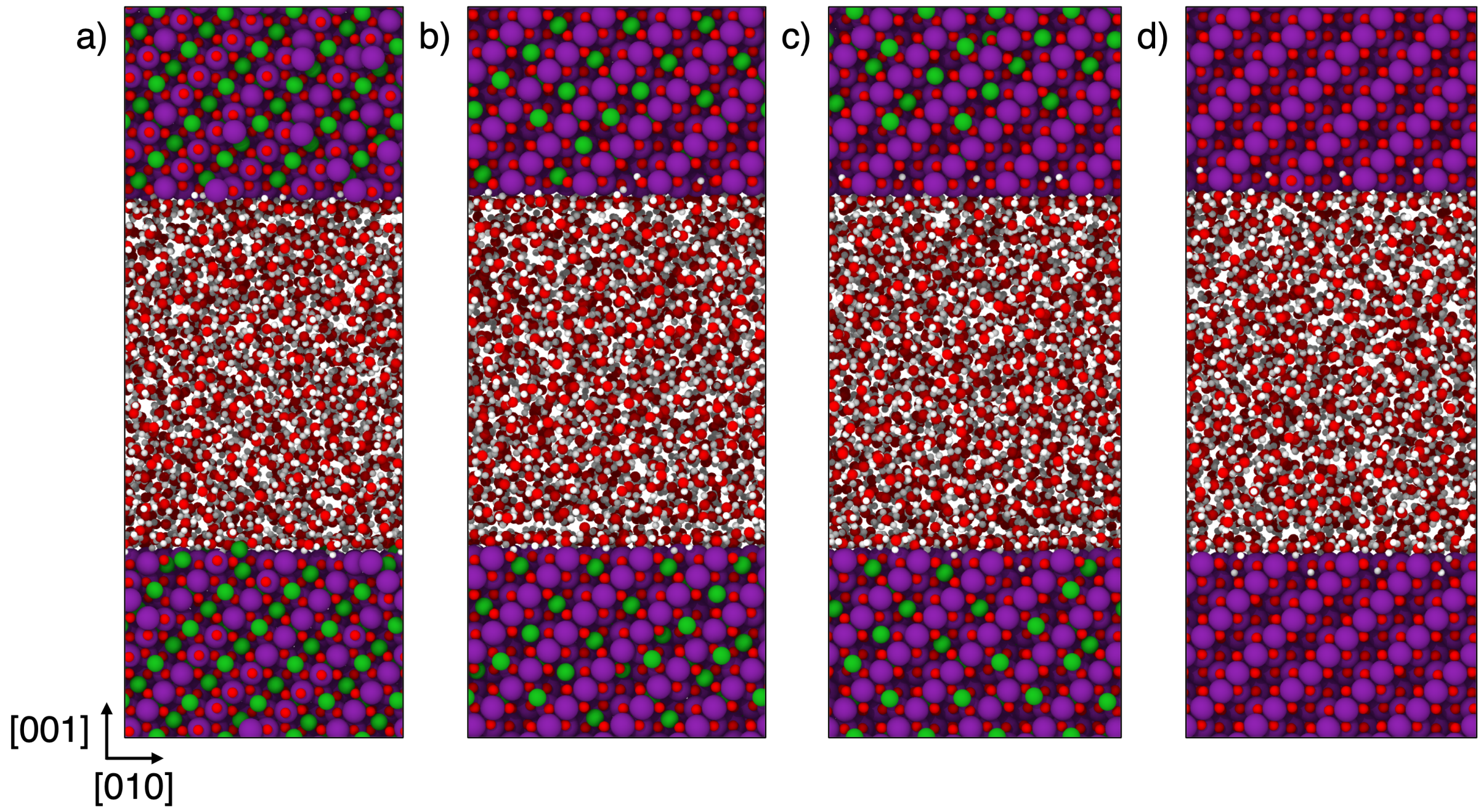

Figure S12: Side views of the equilibrated aqueous LMO interfaces with varying Li content. **a)** shows the stoichiometric $LiMn_2O_4$ interface with $x_{\mathrm{Li}} = 1.0$; **b)** and **c)** show the two LMO interface systems with $x_{\mathrm{Li}} = 0.5$ ($Li^{\mathrm{A}}_{0.5}Mn_2O_4$ and $Li^{\mathrm{B}}_{0.5}Mn_2O_4$, respectively); and **d)** shows the delithiated λ-Mn_2O_4 interface with $x_{\mathrm{Li}} = 0.0$. All simulation boxes are ≈ 80 Å tall and ≈ 30 Å wide. Atomic species are color-coded as follows: H (white), O (red), Li (green), and Mn (purple). The interfaces were rendered using OVITO Pro (version 3.14.1).[50]

within the expected range, given the force root-mean-squared error (RMSE) of 184–185 meV/Å obtained for the test set. While occasional outliers are visible, our investigation revealed that these predominantly originate from atoms located directly at the solid-liquid interface. This behavior is expected: the interfacial region exhibits the highest chemical reactivity and strongest local interactions, resulting in larger absolute atomic forces. Consequently, the standard deviation scales accordingly, even though the relative prediction error remains stable across the trajectory.

S2.2.2 Charge Conservation

Like most machine learning potentials, second-generation HDNNPs do not explicitly include information about electronic degrees of freedom. To address this, we integrated an additional HDNNS[7] to determine the spin state of the Mn ions based on their local atomic environments. As detailed in Section S1.5, these predicted atomic spins are used to assign integer oxidation states to individual ions. However, because this approach does not strictly enforce global charge conservation across the simulation cell by construction, the validity and stability of the assigned oxidation states must be explicitly verified.

In this work, we investigated three distinct lithiation states ($x_{\mathrm{Li}} = 1.0$, 0.5, and 0.0), where the Li content directly dictates the ratio of mixed-valence Mn^{3+}/Mn^{4+} ions. For the stoichiometric $x_{\mathrm{Li}} = 1.0$ system, this ratio is nominally 1:1, yielding an expected average Mn oxidation state of +3.5. At the intermediate concentration ($x_{\mathrm{Li}} = 0.5$), the expected average shifts to +3.75. Finally, in the fully delithiated λ-Mn_2O_4 limit ($x_{\mathrm{Li}} = 0.0$), all Mn ions should formally oxidize to Mn^{4+}. To verify that our HDNNS model correctly captures this stoichiometry-dependent oxidation state balance without explicit global constraints, we calculated the mean oxidation state, $\bar{q}_{\mathrm{Mn}}$, of all Mn ions for every configuration along the trajectory. The temporal evolution of $\bar{q}_{\mathrm{Mn}}$ during the production simulations is presented in Figure S17.

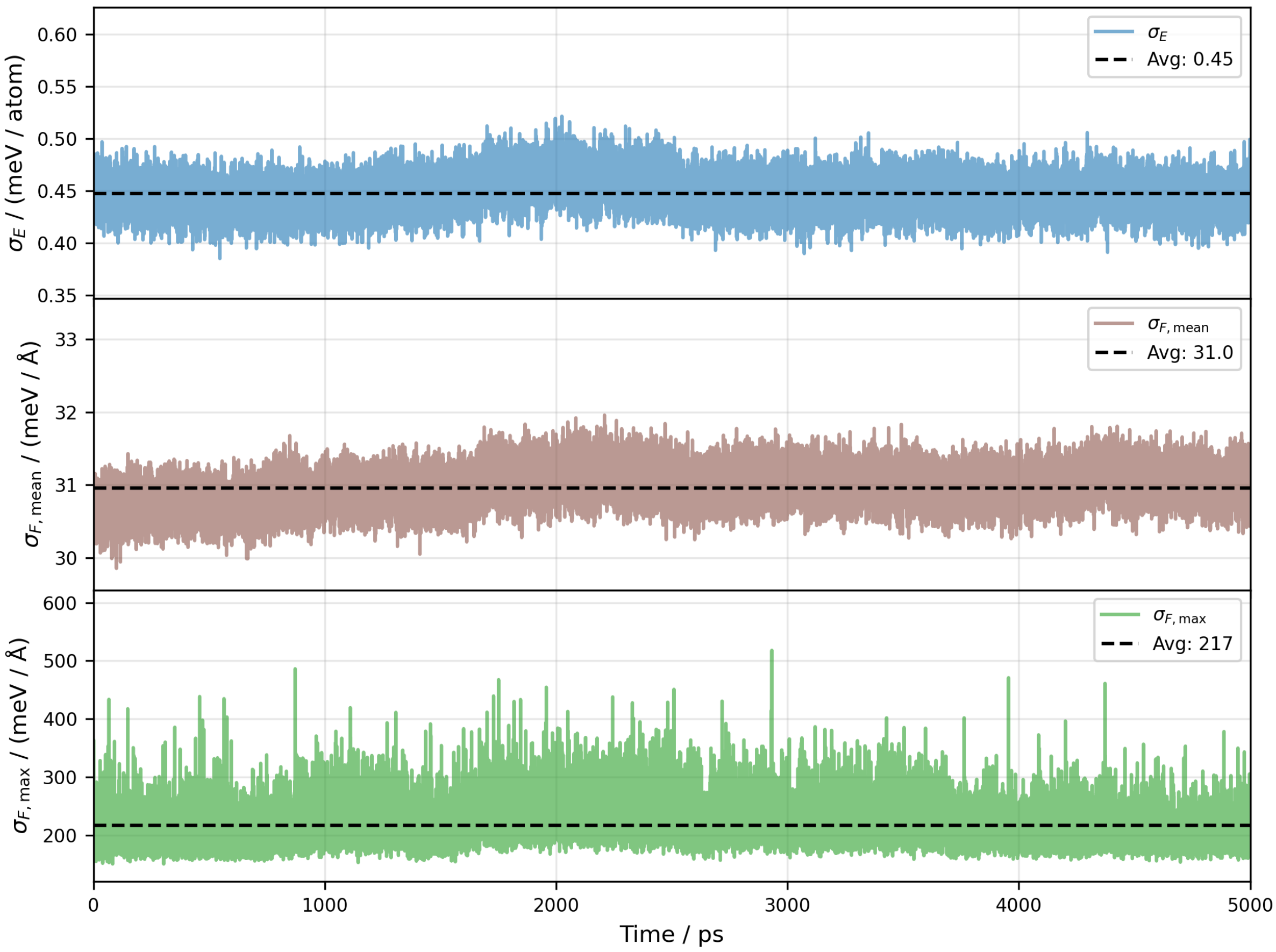

Figure S13: Evaluation of the committee disagreement during the 5 ns production MD simulation of the aqueous stoichiometric $LiMn_2O_4$ interface ($x_{\mathrm{Li}} = 1.0$). The panels illustrate the time evolution of the standard deviation for the predicted total energy (σ_E), the mean standard deviation across all atomic force components ($\sigma_{F,\mathrm{mean}}$), and the maximum standard deviation of any single force component ($\sigma_{F,\mathrm{max}}$).

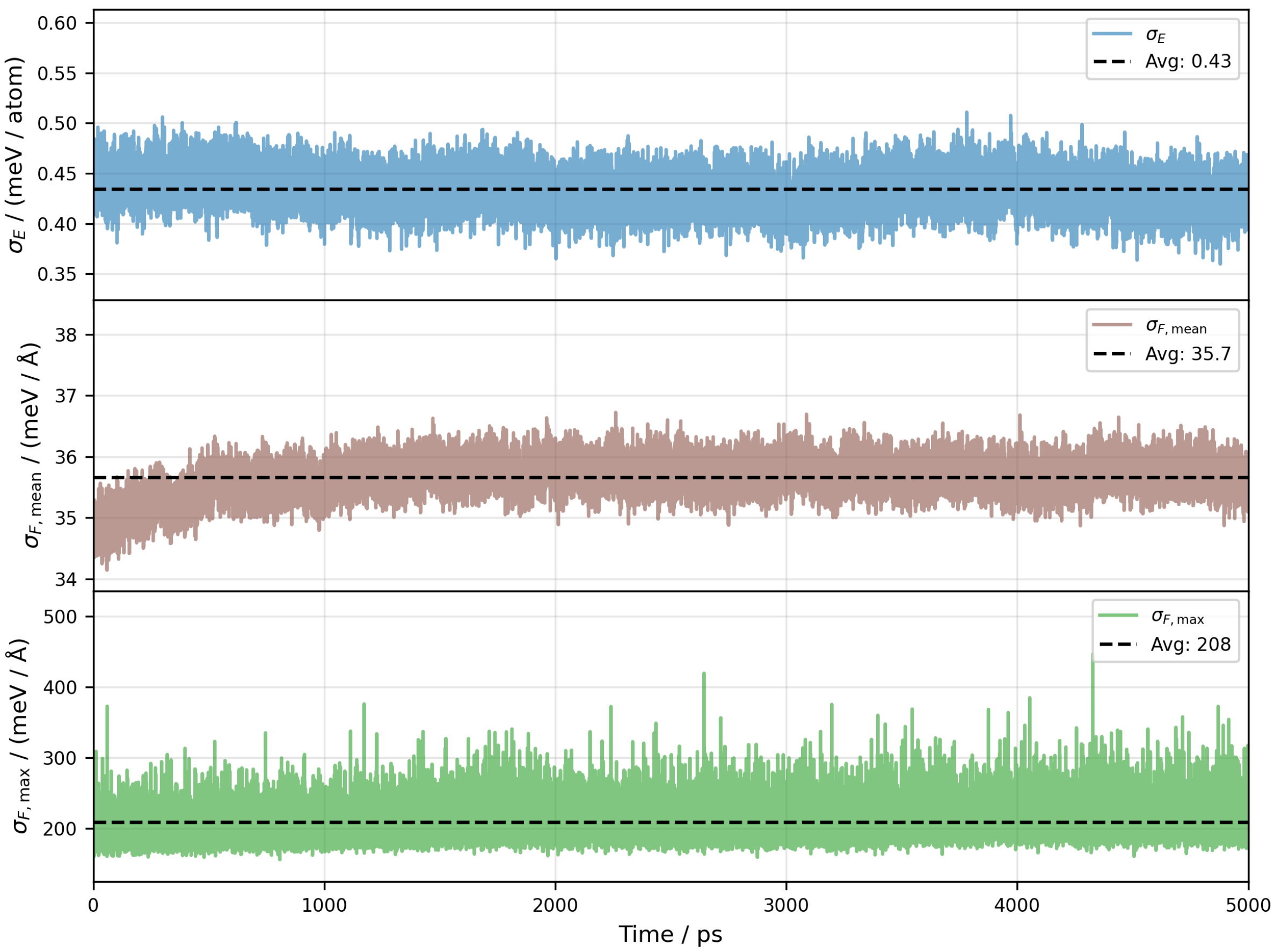

Figure S14: Evaluation of the committee disagreement during the 5 ns production MD simulation of the aqueous $Li^{A}_{0.5}Mn_2O_4$ interface ($x_{Li} = 0.5$). The panels illustrate the time evolution of the standard deviation for the predicted total energy (σ_E), the mean standard deviation across all atomic force components ($\sigma_{F,\text{mean}}$), and the maximum standard deviation of any single force component ($\sigma_{F,\text{max}}$).

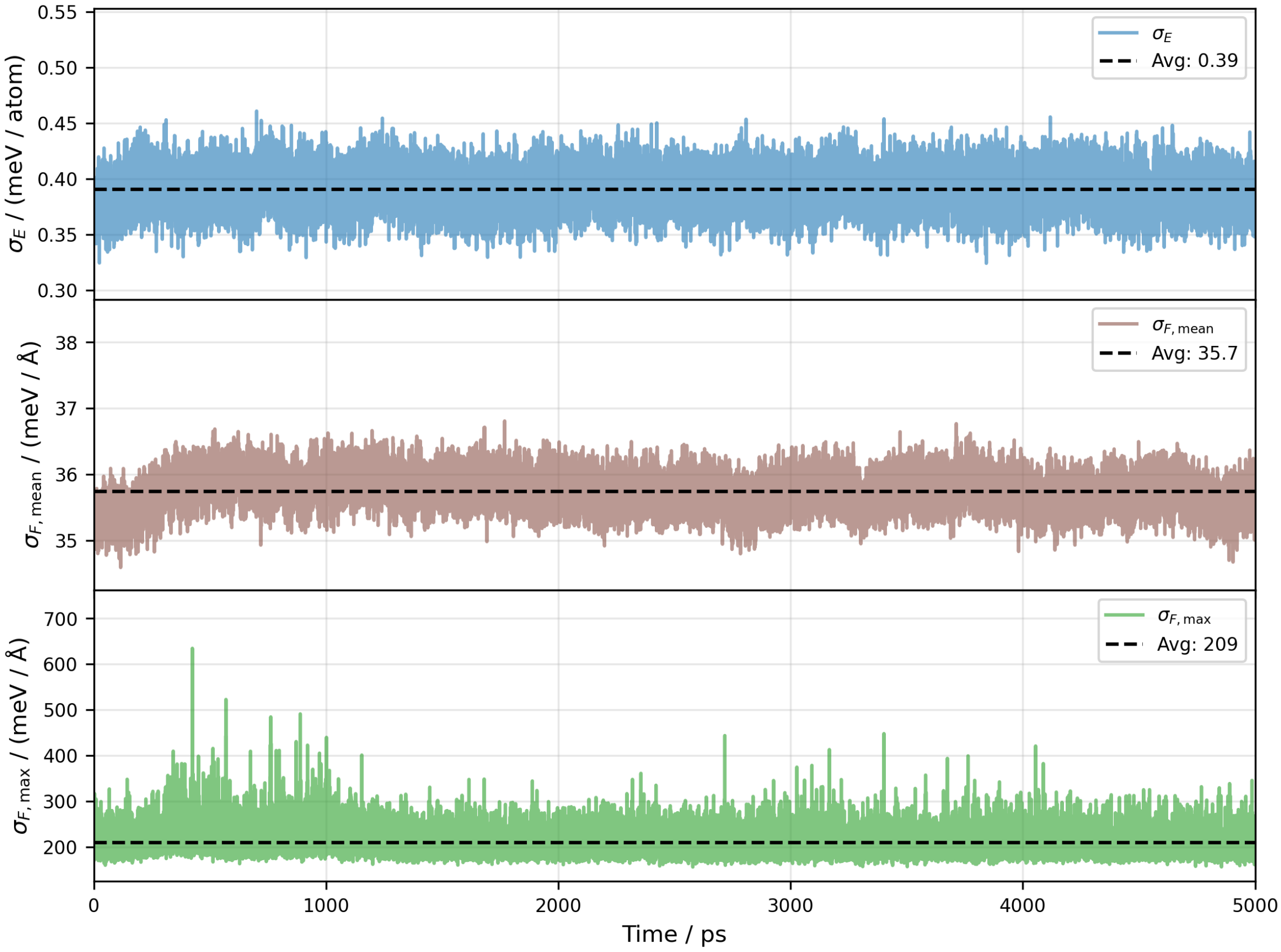

Figure S15: Evaluation of the committee disagreement during the 5 ns production MD simulation of the aqueous $Li^{B}_{0.5}Mn_2O_4$ interface ($x_{Li} = 0.5$). The panels illustrate the time evolution of the standard deviation for the predicted total energy (σ_E), the mean standard deviation across all atomic force components ($\sigma_{F,\mathrm{mean}}$), and the maximum standard deviation of any single force component ($\sigma_{F,\mathrm{max}}$).

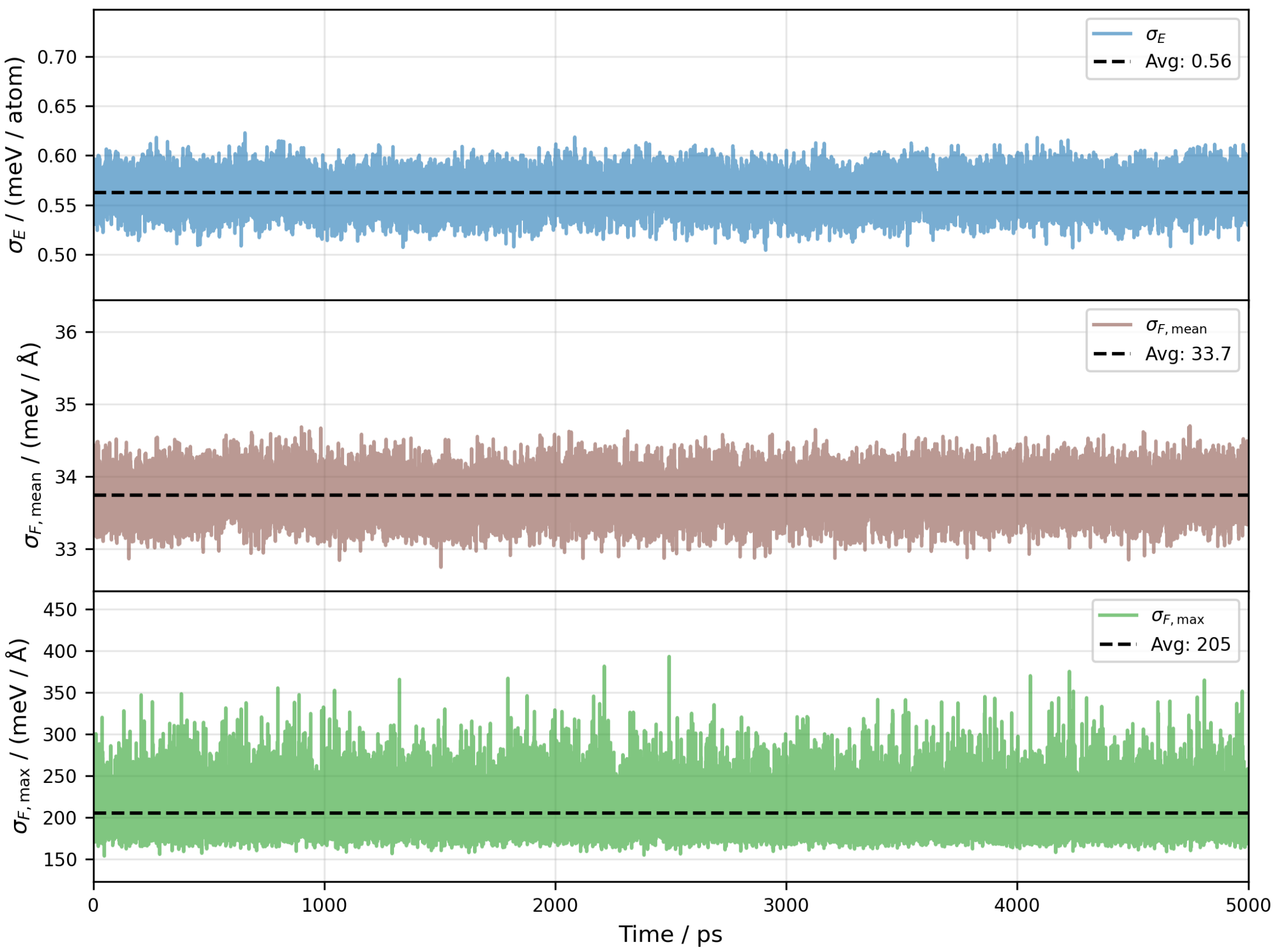

Figure S16: Evaluation of the committee disagreement during the 5 ns production MD simulation of the aqueous λ-Mn_2O_4 interface ($x_{\mathrm{Li}} = 0.0$). The panels illustrate the time evolution of the standard deviation for the predicted total energy (σ_E), the mean standard deviation across all atomic force components ($\sigma_{F,\mathrm{mean}}$), and the maximum standard deviation of any single force component ($\sigma_{F,\mathrm{max}}$).

The predicted mean oxidation state remains highly stable across all four investigated systems. Fluctuations around the temporal mean are approximately 0.5% for the $x_{\mathrm{Li}} = 1.0$ and 0.5 configurations, and drop to $\approx 0.05\%$ for the fully delithiated $x_{\mathrm{Li}} = 0.0$ case. The computed averages are in excellent agreement with theoretical expectations: we obtain an average Mn oxidation state of +3.49 for the stoichiometric LMO cell, and +3.80 and +3.79 for the $Li^{A}_{0.5}Mn_2O_4$ and $Li^{B}_{0.5}Mn_2O_4$ cells, respectively. For the delithiated MO system, the mean converges precisely to +4.00.

These minor deviations from the ideal oxidation state ratio (< 2%) are consistent with our previous studies applying this HDNNS methodology to bulk LMO and stoichiometric interfaces.[7, 5] Physically, these small uncertainties arise from the dynamic J-T effect and rapid electron hopping events between adjacent Mn sites. During such electronic transitions, the local oxidation state is not uniquely defined by the local atomic environment. This inherent physical ambiguity occasionally results in erroneous predictions by the HDNNS, leading to misclassifications that introduce slight noise into the oxidation state average. This interpretation is reinforced by the results for the fully delithiated MO simulation cell. Because this system lacks hs-Mn^{3+} ions, it exhibits neither J-T distortions nor electron hopping events. Consequently, the oxidation state is unambiguously defined by the local atomic environments, yielding the +4.00 oxidation state with near-perfect accuracy and an order of magnitude lower fluctuation. Finally, we note that any small imbalance in the predicted oxidation states does not affect the quality of the c-HDNNP since no explicit electrostatic interactions are included in the potential.

S2.3 Mn Oxidation State distribution

The spatial distribution of Mn oxidation states within the solid slab is highly dependent on both the overall Li content and its specific local arrangement. We determined these oxidation states using the HDNNS by predicting the absolute atomic spin values of the Mn ions for each configuration in the production trajectories based on their local atomic environment, subsequently applying the classification criteria detailed in Section S1.5. The resulting layer-resolved oxidation state profiles for the various aqueous interfaces are presented in Figures S18 to S21.

For the stoichiometric slab ($x_{\mathrm{Li}} = 1.0$, Figure S18), the interior exhibits the expected bulk-like 1:1 ratio of Mn^{3+} and Mn^{4+}. However, moving toward the solid-liquid boundaries reveals pronounced electronic reconstructions. Because the initial cleaved slab presents asymmetric terminations (as discussed in Section S2.1), the oxidation state profiles at the two opposing surfaces differ significantly. On the Li-terminated right-hand facet, the outermost surface layer consists almost exclusively of Mn^{3+}. To compensate for this localized charge imbalance, the immediate subsurface layer adopts an exclusively Mn^{4+} configuration. This alternating, layer-by-layer excess of Mn^{3+} and Mn^{4+} rapidly decreases, converging to the stoichiometric 1:1 ratio toward the center of the slab.

Conversely, the MnO_2-terminated left-hand facet exhibits a mixed-valence surface layer comprising $\approx 64\%$ Mn^{3+} and $\approx 36\%$ Mn^{4+}. This moderate Mn^{3+} excess is similarly compensated in the adjacent subsurface layers before stabilizing into the bulk-like 1:1 regime. This rapid convergence demonstrates the material's ability to screen surface charge imbalances locally through purely electronic (oxidation state) redistributions.

Reassuringly, the specific oxidation state profiles observed at the MnO_2- and Li-terminated surfaces of this asymmetric, as-cleaved slab closely match the distributions found in our prior work on stoichiometric LMO interfaces,[5] where the two terminations were modeled individually using symmetric reconstructed slabs with explicit Li excess or deficit. Our dynamic observations therefore strongly support the static calculations of Kim et al.,[45] who demonstrated that simply cleaved LMO slabs—despite being formally Tasker type III polar interfaces—can stabilize upon relaxation. Our analysis explicitly confirms that the divergent dipole moment inherent to such Tasker III slabs is dynamically neutralized by the spontaneous spatial redistribution of the Mn valences.

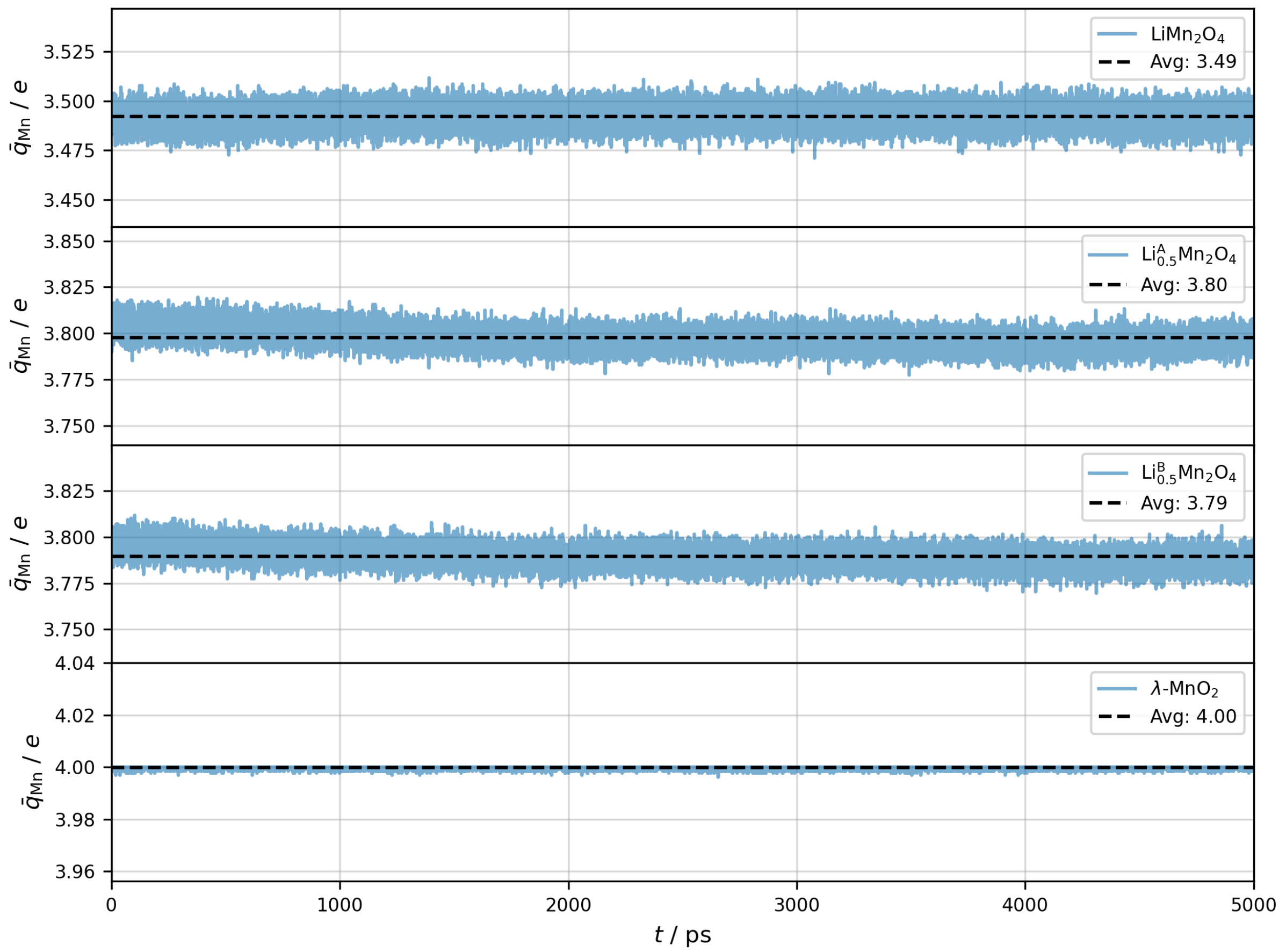

Figure S17: Charge conservation during the four 5 ns production MD trajectories. The panels illustrate the evolution of the predicted mean Mn oxidation state ($\bar{q}_{\mathrm{Mn}}$) across the three distinct lithiation states ($x_{\mathrm{Li}} = 1.0$, 0.5, and 0.0). Note that the $x_{\mathrm{Li}} = 0.5$ state comprises two independent configurations ($Li^A_{0.5}Mn_2O_4$ and $Li^B_{0.5}Mn_2O_4$), yielding four trajectories in total. The minimal fluctuations around the theoretically expected stable means demonstrate robust charge conservation throughout the simulations.

Similarly, for the two half-delithiated systems ($x_{\mathrm{Li}} = 0.5$, configurations $\mathrm{Li}^{\mathrm{A}}_{0.5}\mathrm{Mn_2O_4}$ and $\mathrm{Li}^{\mathrm{B}}_{0.5}\mathrm{Mn_2O_4}$, Figures S19 and S20), the interior regions exhibit the expected bulk-like 1:3 stoichiometric ratio of Mn^{3+} to Mn^{4+}, while the solid-liquid boundaries undergo pronounced electronic reconstructions. The two distinct initial Li distributions—which sample the symmetry breaking associated with populating only one of the two interpenetrating *fcc* sub-lattices of the diamond-like $8a$ sites—naturally result in two different electronic reconstruction profiles.

In the $\mathrm{Li}^{\mathrm{A}}_{0.5}\mathrm{Mn_2O_4}$ slab (Figure S19; cf. the density profile in Figure S25), the Li ions are distributed relatively evenly throughout the bulk, retaining only a slight excess on the right-hand side from the initial configuration. This distribution induces a largely mirror-symmetric oxidation state profile across the slab. Both exposed surfaces exhibit a mixed-valence population comprising $\approx 30\%$ hs-Mn^{3+} and $\approx 70\%$ Mn^{4+}. To compensate for this surface state, the immediate subsurface layer becomes almost exclusively populated by Mn^{4+} ($\approx 100\%$). This localized Mn^{4+} excess then rapidly attenuates, restoring the 1:3 $\mathrm{Mn}^{3+}/\mathrm{Mn}^{4+}$ bulk ratio toward the center of the slab.

In the alternative $\mathrm{Li}^{\mathrm{B}}_{0.5}\mathrm{Mn_2O_4}$ configuration (Figure S20; cf. the density profile in Figure S26), the initial asymmetric Li distribution remains pronounced over the simulation timescale. This leaves the left-hand side of the slab completely devoid of subsurface Li ions, while a slight Li excess persists near the right-hand boundary. Consequently, this structural asymmetry induces a highly asymmetric oxidation state profile. The fully delithiated left-hand facet consists exclusively of Mn^{4+} ions, whereas the right-hand facet exhibits a mixed-valence distribution comprising $\approx 40\%$ hs-Mn^{3+} and 60% Mn^{4+}. As observed in the $\mathrm{Li}^{\mathrm{A}}_{0.5}\mathrm{Mn_2O_4}$ system, this surface charge imbalance is rapidly compensated within the adjacent subsurface layers, attenuating toward the expected bulk ratio in the center of the slab.

Finally, the fully delithiated λ-$\mathrm{MnO_2}$ configuration (Figure S21; cf. the density profile in Figure S27) exhibits the most straightforward oxidation state profile. Because the system is entirely devoid of Li ions and presents symmetric, non-polar Tasker type II facets, no electronic surface reconstruction is required. Consequently, every Mn ion uniformly adopts the Mn^{4+} oxidation state throughout the entire slab, from the bulk interior to the solid-liquid interfaces.

S2.4 Cooperative Jahn-Teller Effect

The hs-Mn^{3+} ions are J-T active, resulting in a pronounced distortion of their octahedral $\mathrm{MnO_6}$ coordination environment. At interfaces, these local distortions tend to couple, forming a long-range cooperative J-T effect with collinear orbital ordering.[51, 52] To investigate the preferred direction of this long-range order, we resolved the average Mn–O bond lengths within the $\mathrm{MnO_6}$ octahedra along the x, y, and z axes throughout the production simulations. The resulting Mn–O bond distances as a function of the lithiation state (x_{Li}) are illustrated in Figure S22. We find that the $\mathrm{MnO_6}$ octahedral elongation is aligned along the [001] surface normal. This leads to a distinct splitting between the extended z-directed bonds (≈ 2.07 Å) and the x- and y-directed bonds, which remain identical and significantly shorter (≈ 1.92 Å). This unidirectional alignment can be rationalized as the minimization of elastic strain within the rigid xy-plane of the infinite bulk crystal.

As the Li content decreases, the magnitude of the gap between the z and xy bond lengths progressively shrinks due to the reduced overall concentration of J-T active Mn^{3+} species within the slab. Furthermore, the average length of the x- and y-directed bonds slightly increases (≈ 1.94 Å), indicating that the cooperative long-range order becomes less stable, allowing a fraction of the remaining J-T active $\mathrm{MnO_6}$ octahedra to distort locally within the xy-plane. Finally, as expected, in the fully delithiated MO slab, the Mn–O distances are identical along all three Cartesian axes (≈ 1.92 Å), perfectly consistent with the undistorted, highly symmetric octahedral coordination environment of the J-T inactive Mn^{4+} ions.

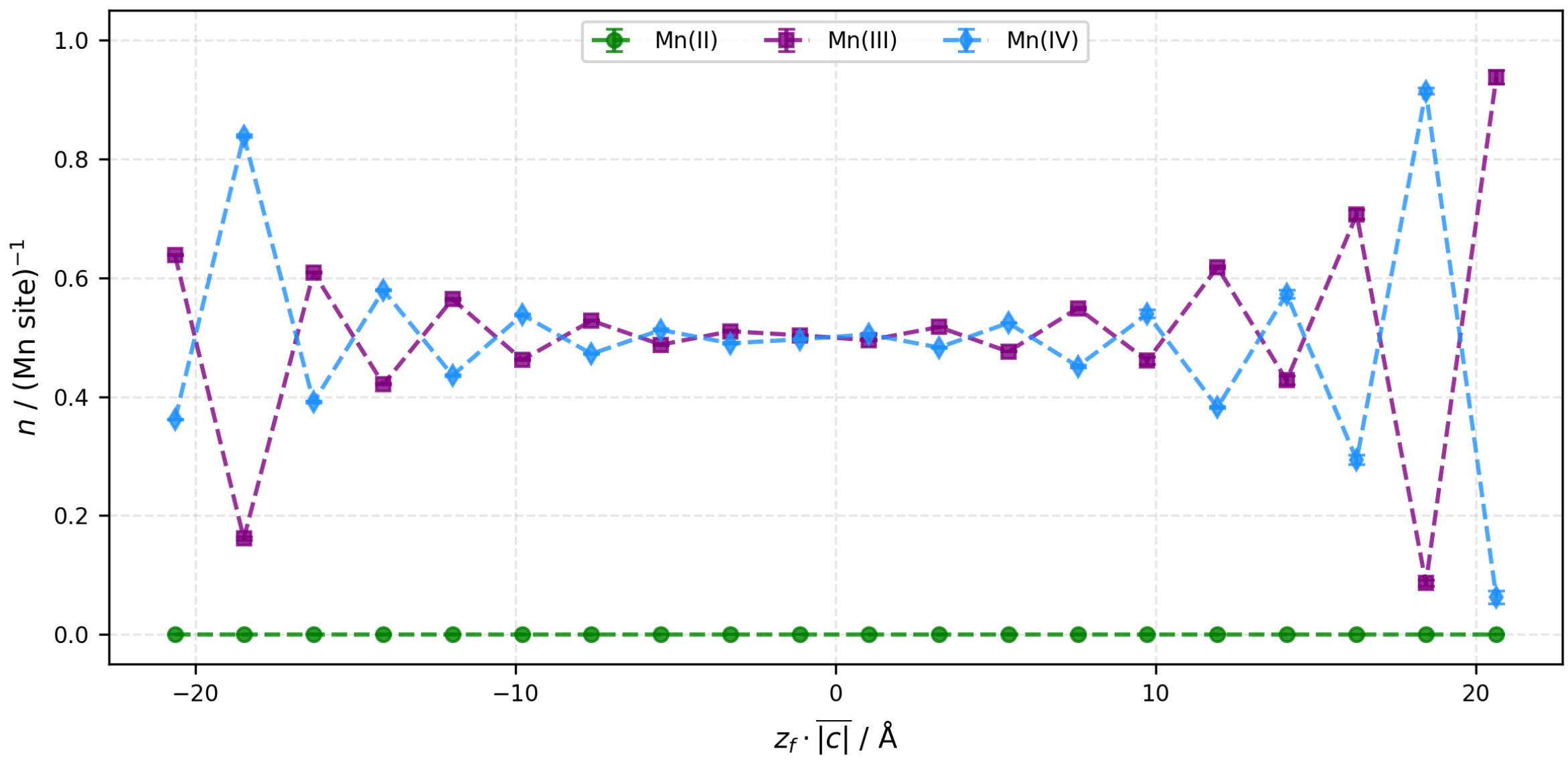

Figure S18: Layer-resolved Mn oxidation state distribution of the solid slab along the [001] surface normal of the aqueous stoichiometric $LiMn_2O_4$ interface ($x_{Li} = 1.0$), averaged over the 3 ns production simulation. The error bars represent the standard error of the mean (SEM), calculated with Bessel's correction via block averaging the trajectory over three consecutive 1 ns windows. To decouple the spatial distributions from the cell volume fluctuations inherent to the NpT ensemble, the atomic positions at each timestep were transformed into fractional coordinates and subsequently rescaled using the time-averaged lattice parameters ($z_f \cdot |\bar{c}|$). Furthermore, to eliminate artificial smearing from minor center-of-mass (COM) drifts, the entire system was re-centered at each timestep by anchoring the COM of the Mn sub-lattice.

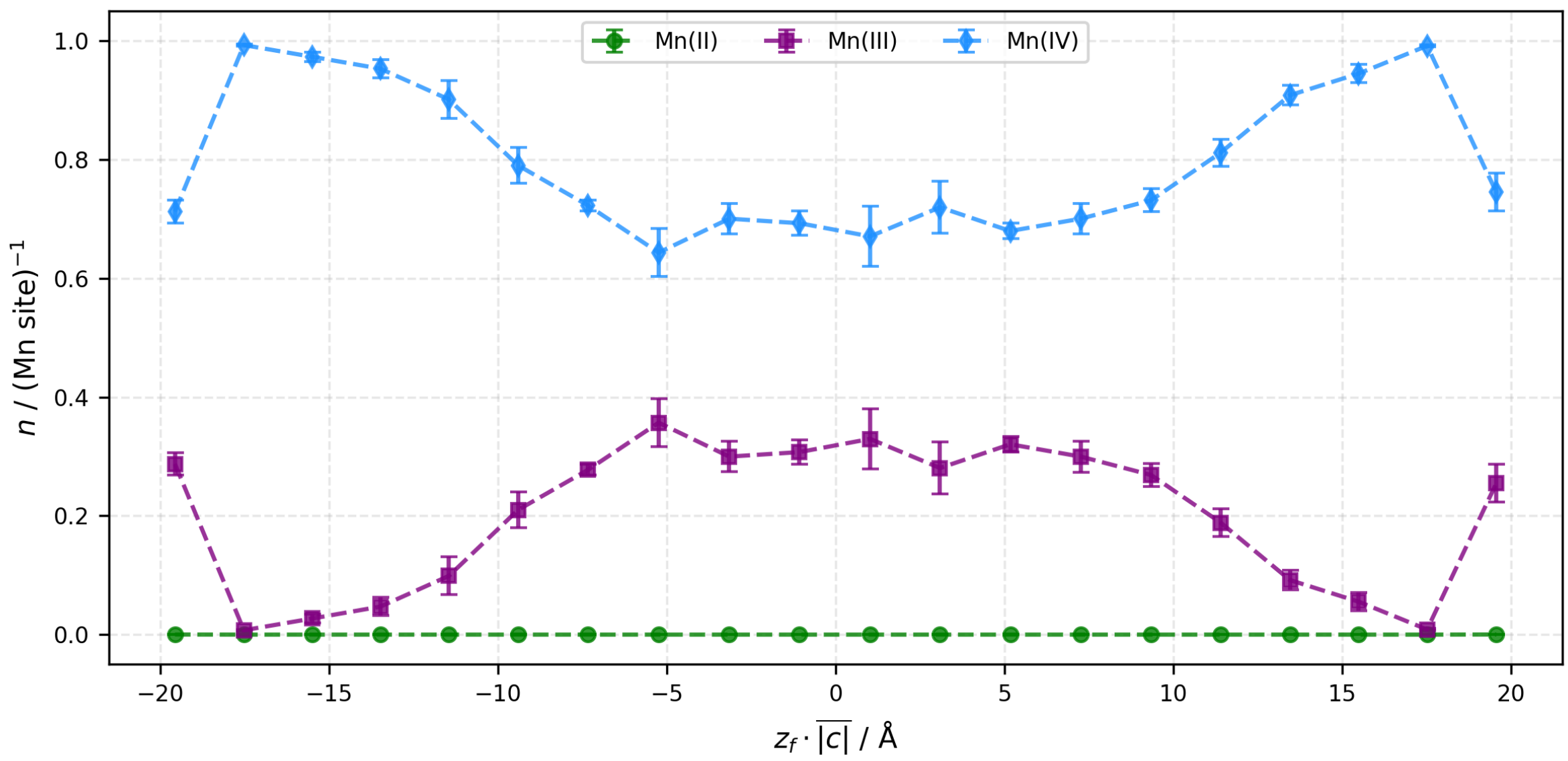

Figure S19: Layer-resolved Mn oxidation state distribution of the solid slab along the [001] surface normal of the aqueous $Li^{A}_{0.5}Mn_2O_4$ interface ($x_{Li} = 0.5$), averaged over the 3 ns production simulation. The error bars represent the standard error of the mean (SEM), calculated with Bessel's correction via block averaging the trajectory over three consecutive 1 ns windows. To decouple the spatial distributions from the cell volume fluctuations inherent to the NpT ensemble, the atomic positions at each timestep were transformed into fractional coordinates and subsequently rescaled using the time-averaged lattice parameters ($z_f \cdot |\bar{c}|$). Furthermore, to eliminate artificial smearing from minor center-of-mass (COM) drifts, the entire system was re-centered at each timestep by anchoring the COM of the Mn sub-lattice.

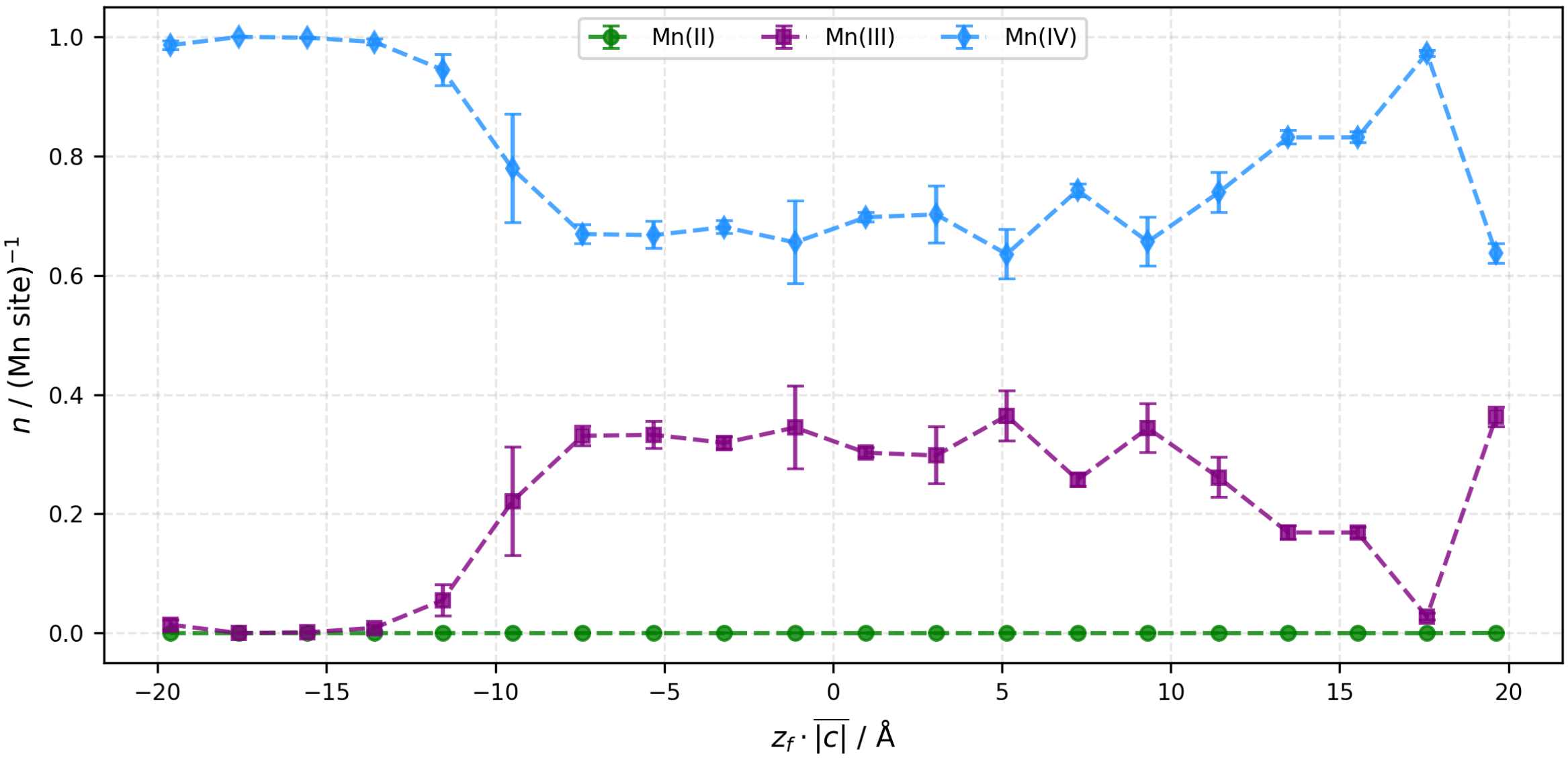

Figure S20: Layer-resolved Mn oxidation state distribution of the solid slab along the [001] surface normal of the aqueous $Li^{B}_{0.5}Mn_2O_4$ interface ($x_{Li} = 0.5$), averaged over the 3 ns production simulation. The error bars represent the standard error of the mean (SEM), calculated with Bessel's correction via block averaging the trajectory over three consecutive 1 ns windows. To decouple the spatial distributions from the cell volume fluctuations inherent to the NpT ensemble, the atomic positions at each timestep were transformed into fractional coordinates and subsequently rescaled using the time-averaged lattice parameters ($z_f \cdot |\bar{c}|$). Furthermore, to eliminate artificial smearing from minor center-of-mass (COM) drifts, the entire system was re-centered at each timestep by anchoring the COM of the Mn sub-lattice.

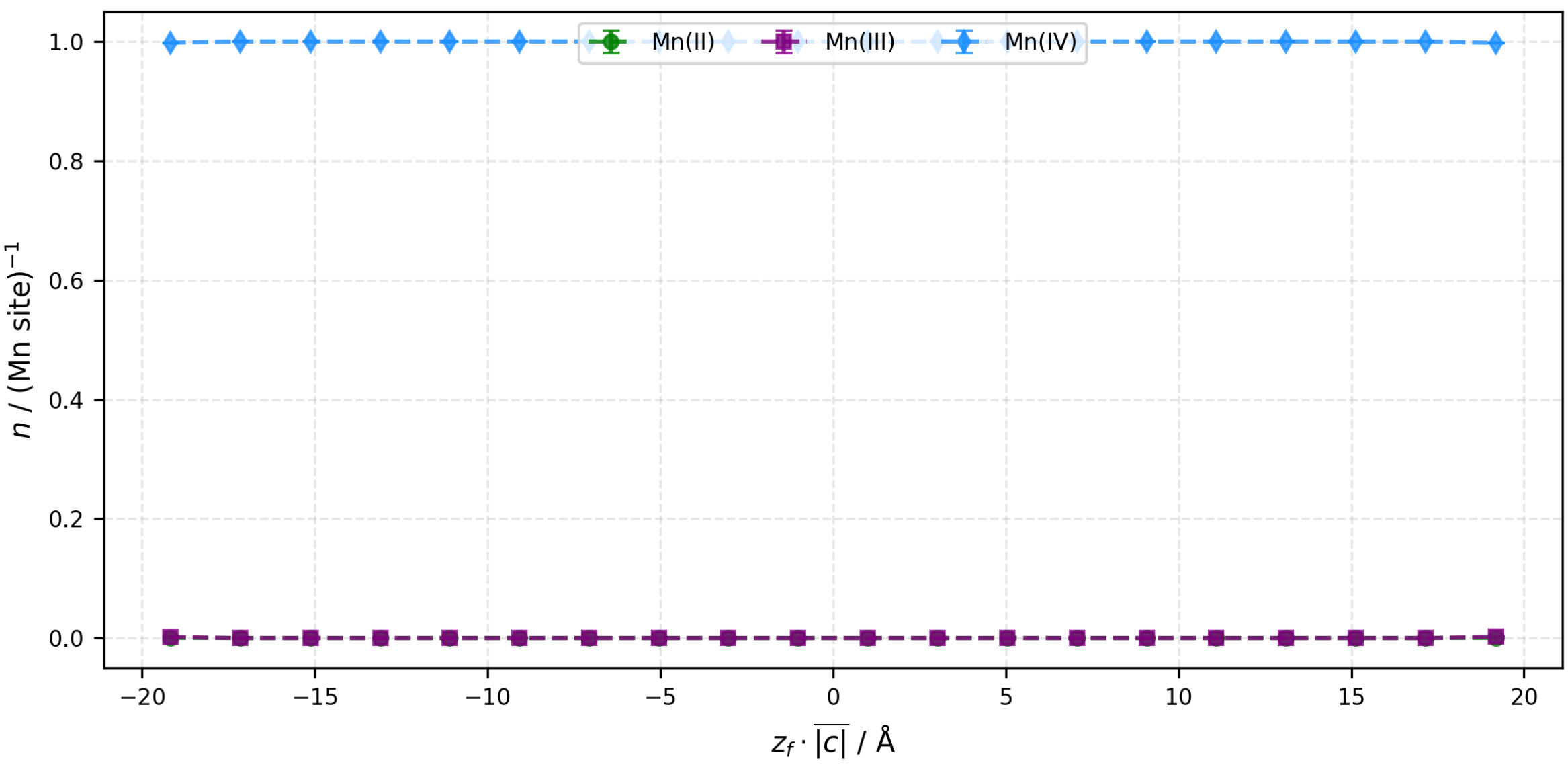

Figure S21: Layer-resolved Mn oxidation state distribution of the solid slab along the [001] surface normal of the aqueous λ-MnO_2 interface ($x_{Li} = 0.0$), averaged over the 3 ns production simulation. The error bars represent the standard error of the mean (SEM), calculated with Bessel's correction via block averaging the trajectory over three consecutive 1 ns windows. To decouple the spatial distributions from the cell volume fluctuations inherent to the NpT ensemble, the atomic positions at each timestep were transformed into fractional coordinates and subsequently rescaled using the time-averaged lattice parameters ($z_f \cdot |\bar{c}|$). Furthermore, to eliminate artificial smearing from minor center-of-mass (COM) drifts, the entire system was re-centered at each timestep by anchoring the COM of the Mn sub-lattice.

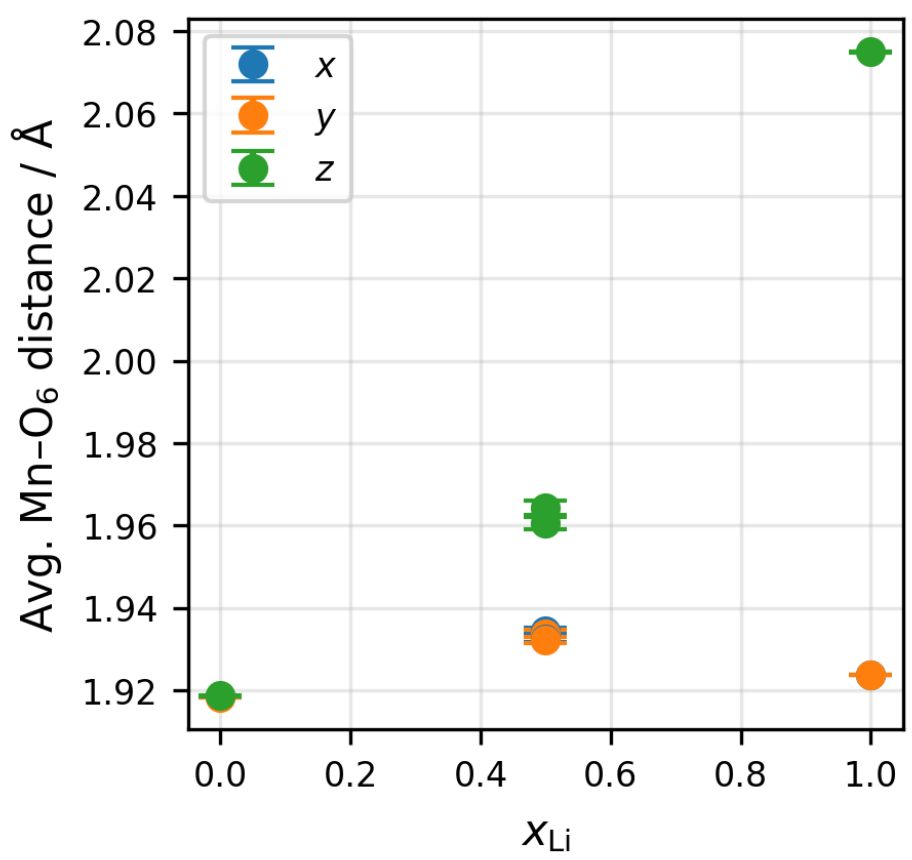

Figure S22: Average Mn–O bond lengths within the MnO_6 octahedra as a function of the lithiation state (x_{Li}), averaged over the 3 ns production simulation. The error bars represent the standard error of the mean (SEM), calculated with Bessel's correction via block averaging the trajectory over three consecutive 1 ns windows. The bond lengths are resolved along the x, y, and z crystallographic axes, with the z-axis aligning with the [001] surface normal. This directional breakdown illustrates the magnitude and preferred orientation of the cooperative Jahn-Teller (J-T) distortion.

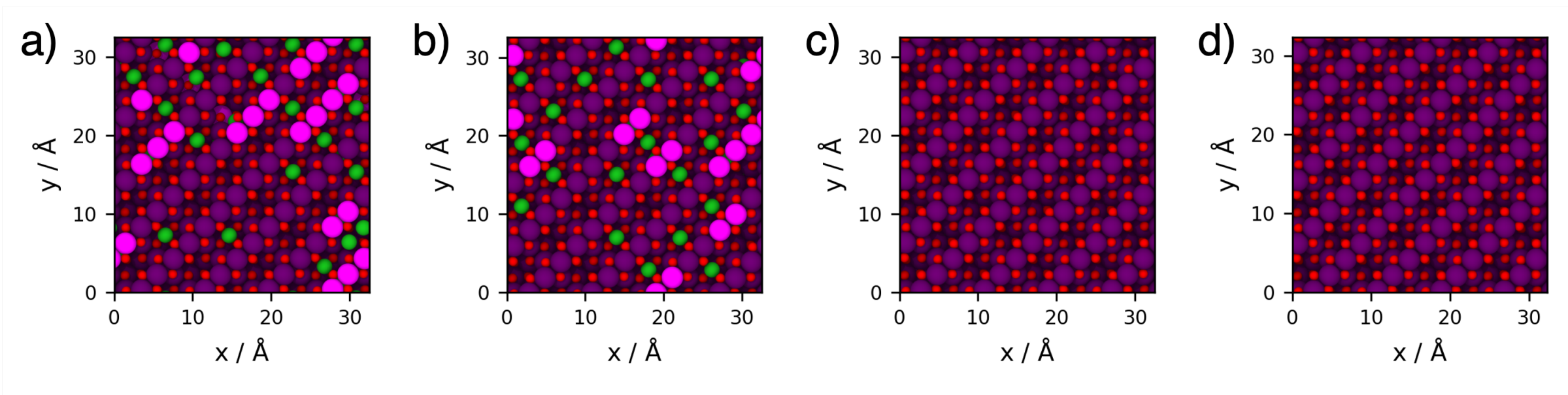

Figure S23: Time-averaged surface configurations of LMO (001) slab facets with different Li content. Each panel shows the mean atomic positions sampled over 3 ns of NpT MD simulation, with the aqueous phase omitted for clarity. To account for thermal fluctuations in the NpT ensemble, lattice parameters were averaged over the trajectory and atomic positions rescaled accordingly. Surface environments are labeled as: **a)** $\mathrm{Li}^{\mathrm{B}}_{0.5}\mathrm{Mn_2O_4}(001)$, **b)** $\mathrm{Li}^{\mathrm{A}}_{0.5}\mathrm{Mn_2O_4}(00\bar{1})$, **c)** $\mathrm{Li}^{\mathrm{B}}_{0.5}\mathrm{Mn_2O_4}(00\bar{1})$, and **d)** λ-$\mathrm{MnO_2}(00\bar{1})$. Atomic species are color-coded as follows: O (red), Li (green), Mn^{3+} (magenta), and Mn^{4+} (dark purple). Oxidation states were assigned via time-averaged atomic spins predicted by the HDNNS.

S2.5 Remaining Time-averaged Surface Facets

In the main text, we showed the time-averaged surface configurations of the $\mathrm{LiMn_2O_4}(001)$, $\mathrm{LiMn_2O_4}(00\bar{1})$, $\mathrm{Li}^{\mathrm{A}}_{0.5}\mathrm{Mn_2O_4}(001)$, and λ-$\mathrm{MnO_2}(001)$. The remaining surface configurations ($\mathrm{Li}^{\mathrm{B}}_{0.5}\mathrm{Mn_2O_4}(001)$, $\mathrm{Li}^{\mathrm{A}}_{0.5}\mathrm{Mn_2O_4}(00\bar{1})$, $\mathrm{Li}^{\mathrm{B}}_{0.5}\mathrm{Mn_2O_4}(00\bar{1})$, and λ-$\mathrm{MnO_2}(00\bar{1})$) are presented in Figure S23, sorted according to increasing average surface oxidation state. As discussed in Section S2.3, the $\mathrm{Li}^{\mathrm{B}}_{0.5}\mathrm{Mn_2O_4}(001)$ and $\mathrm{Li}^{\mathrm{A}}_{0.5}\mathrm{Mn_2O_4}(00\bar{1})$ surface facets feature a mixed-valence Mn population, whereas the $\mathrm{Li}^{\mathrm{B}}_{0.5}\mathrm{Mn_2O_4}(00\bar{1})$ and λ-$\mathrm{MnO_2}(00\bar{1})$ facets are exclusively populated by Mn^{4+} due to the complete absence of interfacial Li ions.

S2.6 Density Profiles

The density profiles in Figures S24 to S27 illustrate the structural evolution of the aqueous interfaces along the [001] surface normal as a function of the lithiation state. In the stoichiometric system ($x_{\mathrm{Li}} = 1.0$, Figure S24), the interior of the slab exhibits bulk-like crystalline ordering,

characterized by periodic density oscillations of the Li, Mn, and O sub-lattices. This periodicity is disrupted near the solid-liquid boundaries. Because the initial cleaved slab presents asymmetric terminations (as discussed in Section S2.1), the structural response at the two interfaces differs significantly. On the Li-terminated facet (right-hand side), interfacial dynamics drive surface Li ions to migrate into the underlying MnO_2 layer, occupying initially vacant octahedral $16c$ sites. This rearrangement, driven by inter-ionic repulsion at the interface, is confined to the surface layers and does not propagate into the bulk. In contrast, on the MnO_2-terminated facet (left-hand side), the subsurface Li ions remain stably anchored within their native tetrahedral $8a$ sites.

Strikingly, the density profile of the liquid-phase oxygen exhibits a distinct double peak exclusively at the MnO_2-terminated surface. This bimodal solvation structure directly stems from the mixed-valence nature of this specific facet (see Section S2.3). While the Li-terminated side is almost exclusively populated by hs-Mn^{3+} ions, approximately 36% of the surface Mn species on the MnO_2-terminated facet are in the +4.0 oxidation state. Consequently, the interface presents a mixture of J-T distorted and undistorted adsorption sites. As detailed in Section S2.4, the cooperative J-T effect is predominantly aligned along the [001] surface normal. Because Mn^{4+} lacks an electron in the antibonding e_g orbital, it forms substantially shorter bonds with adsorbed interfacial species compared to the J-T active hs-Mn^{3+}.[7] This difference in the coordination geometry naturally resolves the interfacial oxygen density into two distinct populations: a closer peak corresponding to species adsorbed at Mn^{4+} sites, and a broader, more distant peak corresponding to species at hs-Mn^{3+} sites. The higher intensity of the outer peak perfectly mirrors the higher relative concentration of hs-Mn^{3+} ($\approx 64\%$) over Mn^{4+} ($\approx 36\%$) on this surface.

This bimodal solvation structure is similarly evident at the mixed-valence surfaces of the half-delithiated ($x_{\mathrm{Li}} = 0.5$) interface systems. For the $Li^{A}_{0.5}Mn_2O_4$ interface, both exposed facets exhibit a mixed-valence Mn population comprising $\approx 30\%$ hs-Mn^{3+} and 70% Mn^{4+}. Consequently, the distribution of the bimodal oxygen peak is inverted relative to the MnO_2-terminated facet of the stoichiometric LMO interface system: the closer peak (associated with Mn^{4+} coordinate bonds) gains prominence, while the more distant peak (associated with the longer hs-Mn^{3+} coordinate bonds) is correspondingly diminished.

The alternative $Li^{B}_{0.5}Mn_2O_4$ configuration, initialized with a different Li distribution, presents one mixed-valence (right-hand side) and one single-valence facet (left-hand side). Although Li diffusion between adjacent $8a$ sites and through $16c$ sites is observed, evidenced by an increase in Li density at the $16c$ sites, global redistribution across the slab is kinetically hindered by the $\approx 0.5\,\mathrm{eV}$ Li diffusion barrier.[4] As a result, the initial asymmetry of the Li distribution persists, with Li being present on the right-hand facet and absent on the left-hand facet. Consequently, the right-hand interface exhibits a bimodal solvation profile closely resembling that of the $Li^{A}_{0.5}Mn_2O_4$ system, consistent with the oxidation state distribution of $\approx 40\%$ hs-Mn^{3+} and 60% Mn^{4+} (see Section S2.3).

In contrast, the left-hand facet of the $Li^{B}_{0.5}Mn_2O_4$ slab is completely devoid of subsurface Li ions and, consequently, exclusively populated by Mn^{4+}. As expected, this interface lacks the bimodal solvation structure entirely, exhibiting instead a single, sharp peak. This structure is identical to the solvation profiles observed at both interfaces of the fully delithiated MO system (Figure S27). This similarity between the purely Mn^{4+} facet of the partially lithiated $Li^{B}_{0.5}Mn_2O_4$ slab and the fully delithiated λ-MnO_2 slab demonstrates that the influence of the Li ions on the oxidation states—and by extension, the interfacial solvent structure—is a localized phenomenon.

Interestingly, for the facets featuring subsurface Li vacancies, an increase in H atom density is observed just below the surface plane. This feature corresponds to H atoms from dissociated water molecules that are bound to surface oxygen sites. In the absence of repulsive subsurface Li cations, these surface hydroxyl protons orient inward, pointing directly toward the vacant $8a$ tetrahedral sites. This rotation into the electron-rich vacancy provides substantial thermodynamic

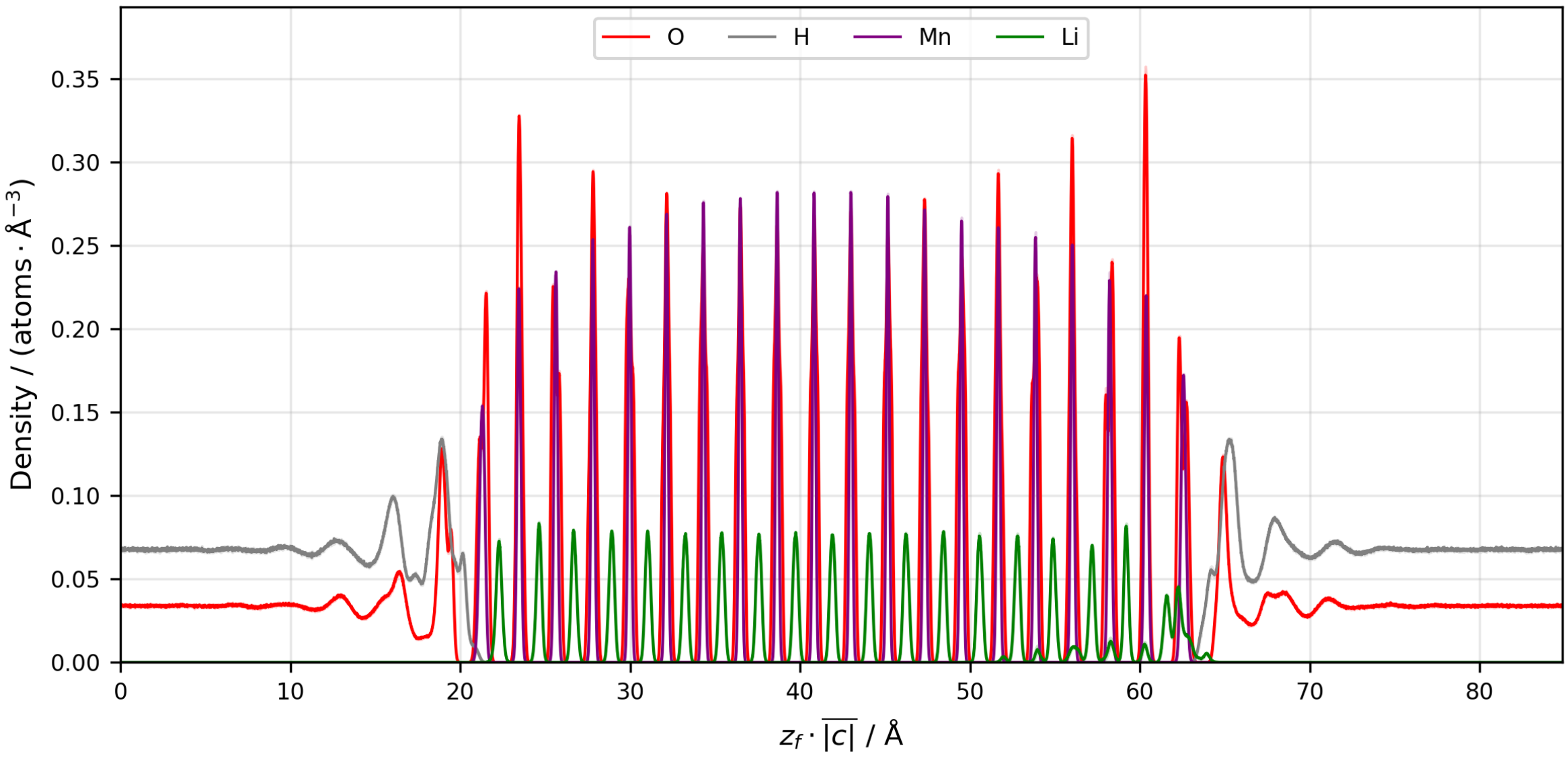

Figure S24: Element-specific density profile along the [001] surface normal of the aqueous stoichiometric $LiMn_2O_4$ interface ($x_{Li} = 1.0$), averaged over the 3 ns production simulation. The shaded regions around the density lines represent the standard error of the mean (SEM), calculated with Bessel's correction via block averaging the trajectory over three consecutive 1 ns windows. To decouple the spatial distributions from the cell volume fluctuations inherent to the NpT ensemble, the atomic positions at each timestep were transformed into fractional coordinates and subsequently rescaled using the time-averaged lattice parameters ($z_f \cdot \overline{|c|}$). Furthermore, to eliminate artificial smearing from minor center-of-mass (COM) drift, the entire system was re-centered at each timestep by anchoring the COM of the Mn sub-lattice.

stabilization, as discussed in the main text, and is in agreement with powder neutron diffraction studies identifying this as the favored orientation for lattice hydroxyls in MnO_2 spinels.[53]

S2.7 Concentration Profiles

To investigate the spatial distribution of water dissociation products at the aqueous LMO interfaces as a function of Li content, we determined the local concentrations of individual oxygen-containing species: H_2O, OH^-, H_3O^+, and unprotonated surface O^{2-}. Species classification was achieved using a geometric distance criterion. For every O atom in the system, the number of adjacent H atoms within a cutoff radius of $r_{OH} \leq 1.25$ Å was evaluated at each configuration of the production simulations. If a given H atom fell within the r_{OH} cutoff of multiple O centers, it was exclusively assigned to the nearest O atom. The spatial coordinate of each identified species was subsequently assigned to its respective center of mass (COM). The resulting concentration profiles of these species along the [001] surface normal are presented in Figures S28 to S31 for the various interfacial systems.

Across all simulated interfaces, water dissociation products are consistently observed, with OH^- exhibiting the highest concentration at the solid-liquid boundary. The two distinct OH^- peaks at the interface correspond to adsorbed OH^- species and protonated surface oxygen sites, respectively. Conversely, H_3O^+ species are comparatively rare and remain predominantly confined to the first adsorption layer. In our previous work,[5] we investigated the specific proton transfer (PT) mechanisms at the stoichiometric aqueous LMO interface in detail. We found that the dominant pathway is a solvent-assisted PT mechanism, wherein a proton from a protonated surface oxygen site is transferred by an interfacial water molecule—temporarily forming a transient H_3O^+ intermediate—to a neighboring unprotonated surface O^{2-} site.

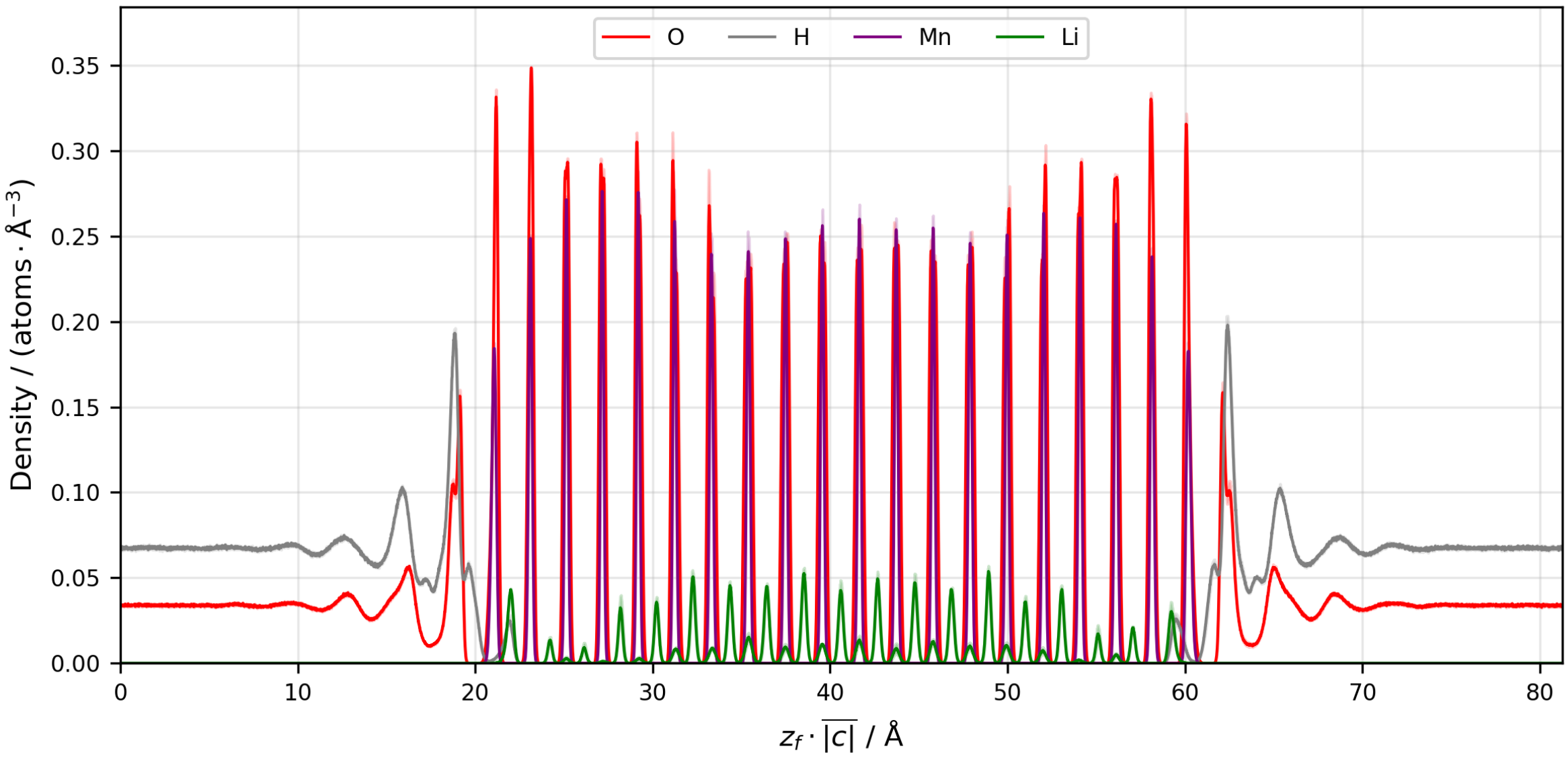

Figure S25: Element-specific density profile along the [001] surface normal of the aqueous $Li^{A}_{0.5}Mn_2O_4$ interface ($x_{Li} = 0.5$), averaged over the 3 ns production simulation. The shaded regions around the density lines represent the standard error of the mean (SEM), calculated with Bessel's correction via block averaging the trajectory over three consecutive 1 ns windows. To decouple the spatial distributions from the cell volume fluctuations inherent to the NpT ensemble, the atomic positions at each timestep were transformed into fractional coordinates and subsequently rescaled using the time-averaged lattice parameters ($z_f \cdot |\bar{c}|$). Furthermore, to eliminate artificial smearing from minor center-of-mass (COM) drift, the entire system was re-centered at each timestep by anchoring the COM of the Mn sub-lattice.

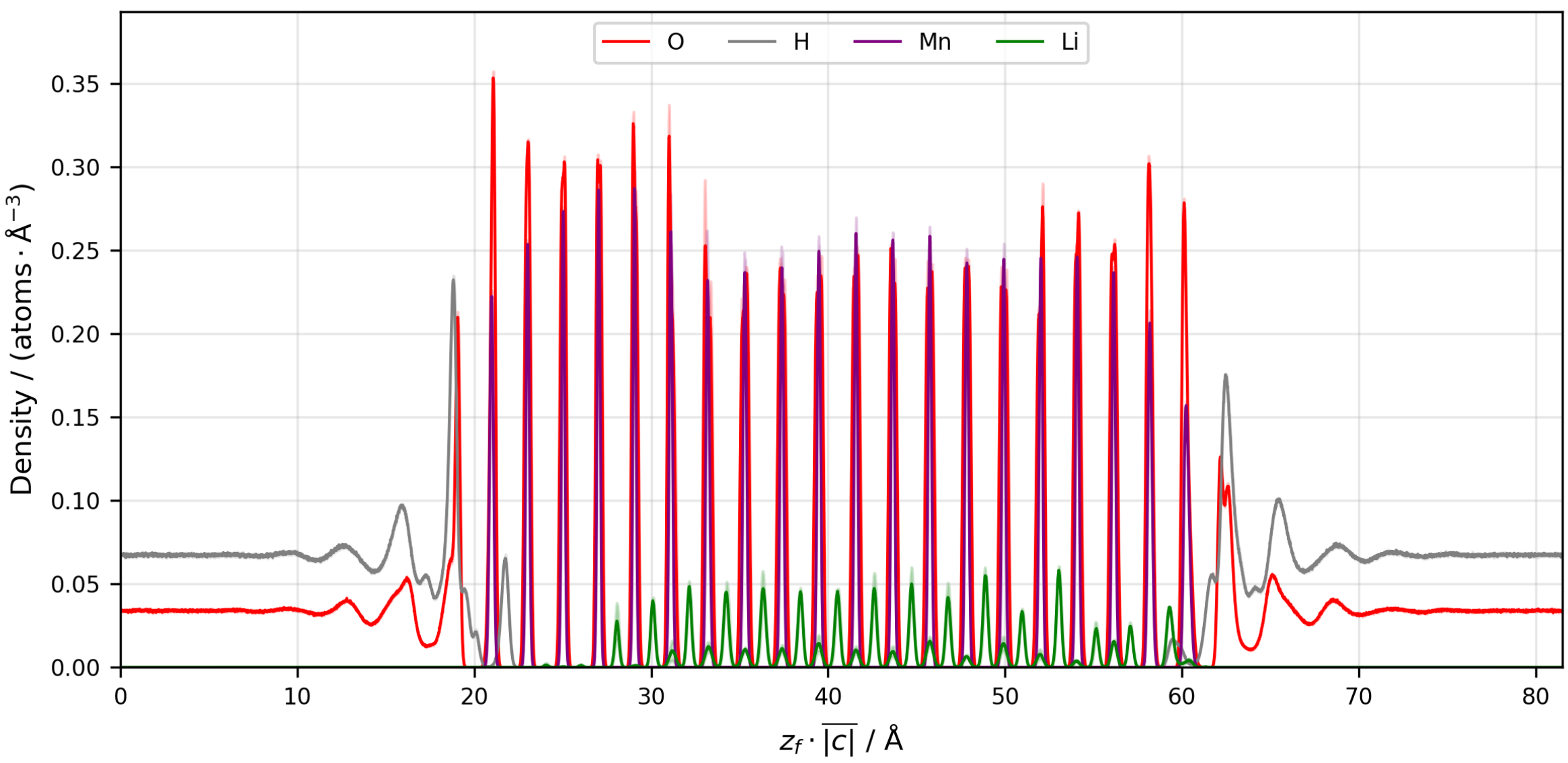

Figure S26: Element-specific density profile along the [001] surface normal of the aqueous $Li^{B}_{0.5}Mn_2O_4$ interface ($x_{Li} = 0.5$), averaged over the 3 ns production simulation. The shaded regions around the density lines represent the standard error of the mean (SEM), calculated with Bessel's correction via block averaging the trajectory over three consecutive 1 ns windows. To decouple the spatial distributions from the cell volume fluctuations inherent to the NpT ensemble, the atomic positions at each timestep were transformed into fractional coordinates and subsequently rescaled using the time-averaged lattice parameters ($z_f \cdot |\bar{c}|$). Furthermore, to eliminate artificial smearing from minor center-of-mass (COM) drift, the entire system was re-centered at each timestep by anchoring the COM of the Mn sub-lattice.

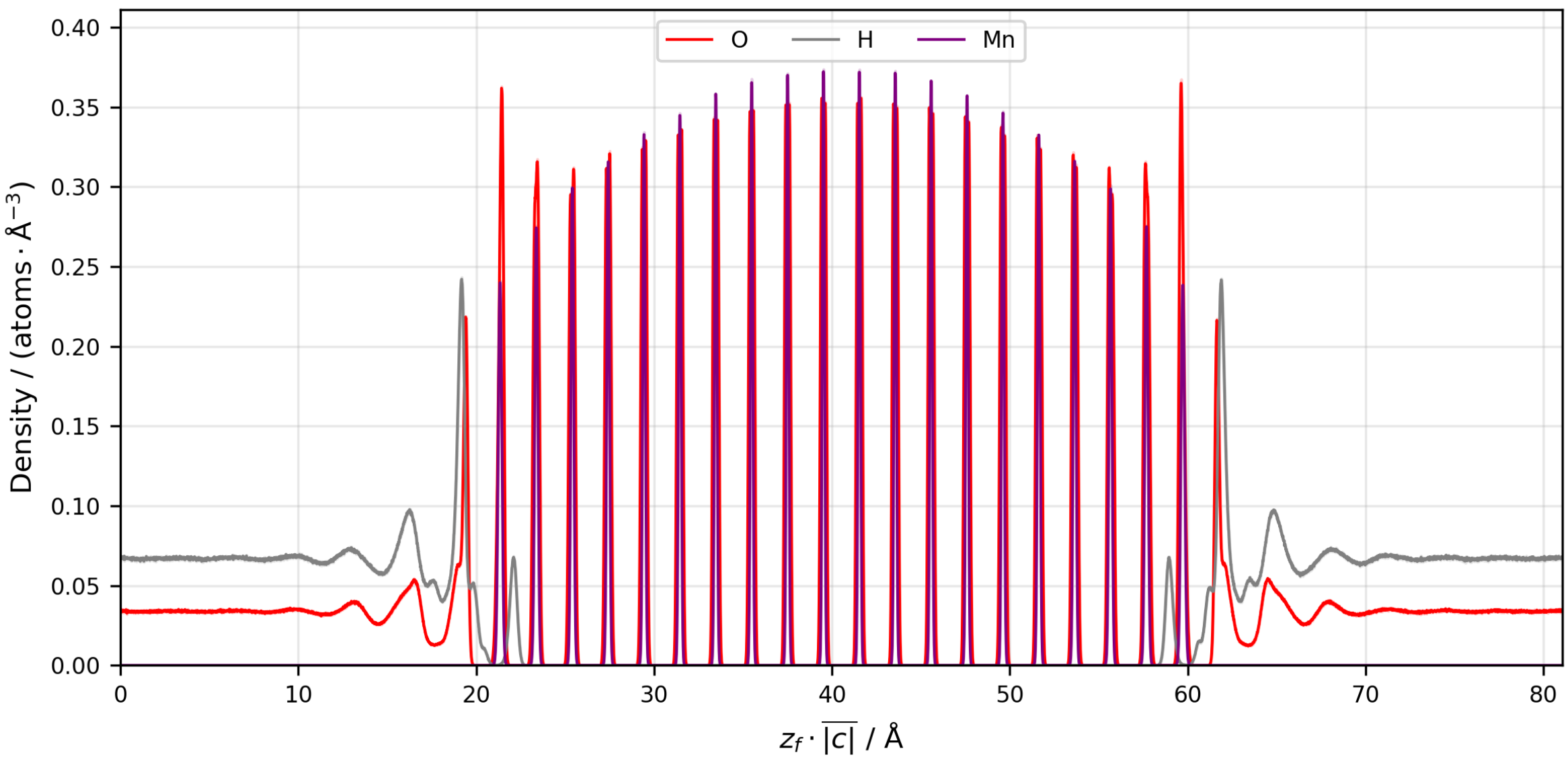

Figure S27: Element-specific density profile along the [001] surface normal of the aqueous λ-MnO_2 interface ($x_{\mathrm{Li}} = 0.0$), averaged over the 3 ns production simulation. The shaded regions around the density lines represent the standard error of the mean (SEM), calculated with Bessel's correction via block averaging the trajectory over three consecutive 1 ns windows. To decouple the spatial distributions from the cell volume fluctuations inherent to the NpT ensemble, the atomic positions at each timestep were transformed into fractional coordinates and subsequently rescaled using the time-averaged lattice parameters ($z_f \cdot |\bar{c}|$). Furthermore, to eliminate artificial smearing from minor center-of-mass (COM) drift, the entire system was re-centered at each timestep by anchoring the COM of the Mn sub-lattice.

As previously discussed in the main text, the interfacial OH^- concentration is strongly dependent on the underlying Mn oxidation state distribution at the surface. A direct comparison between the layer-resolved oxidation states (Section S2.3) and the species concentration profiles reveals a clear correlation: an increased presence of Mn^{4+} species at the surface directly corresponds to an elevated concentration of interfacial OH^-. As demonstrated by the decomposition of the VDOS, the highly oxidized Mn^{4+} ions strongly polarize their coordinating water molecules. This pronounced polarization lowers the activation barrier for proton transfer, ultimately driving the observed increase in water dissociation.

S2.8 Vibrational Density of States Decomposition

S2.8.1 Spatial Definition of Interfacial and Bulk Regions

The H-atom VDOS has been decomposed into interface and background (bulk) contributions to isolate the spectral signatures relevant to the acid-base chemistry of the aqueous LMO interfaces. We define the interface as the solid surface plus the first hydration layer, with the latter spanning from the surface to the first minimum in the liquid-phase oxygen density profile (cf. Section S2.6). Since both slab surfaces are generally different, a rigorous spatial definition of the surface-specific background is required. To prevent artifacts that could arise from including H atoms too close to the opposite slab surface, the slab was centered within the simulation box along the surface normal (the z-axis), as illustrated in Section S2.6. This effectively splits the liquid phase into two distinct regions with ≈ 20 Å of bulk-like water adjacent to each facet. Consequently, the bulk background for the left-hand facet is defined as the liquid region extending from the box boundary at $z = 0$ to the left interfacial density minimum. Similarly, the bulk background for the right-hand facet spans from the right interfacial density minimum to the opposite box boundary at $z = L_z$. This straightforward geometric partitioning ensures identical and independent backgrounds for

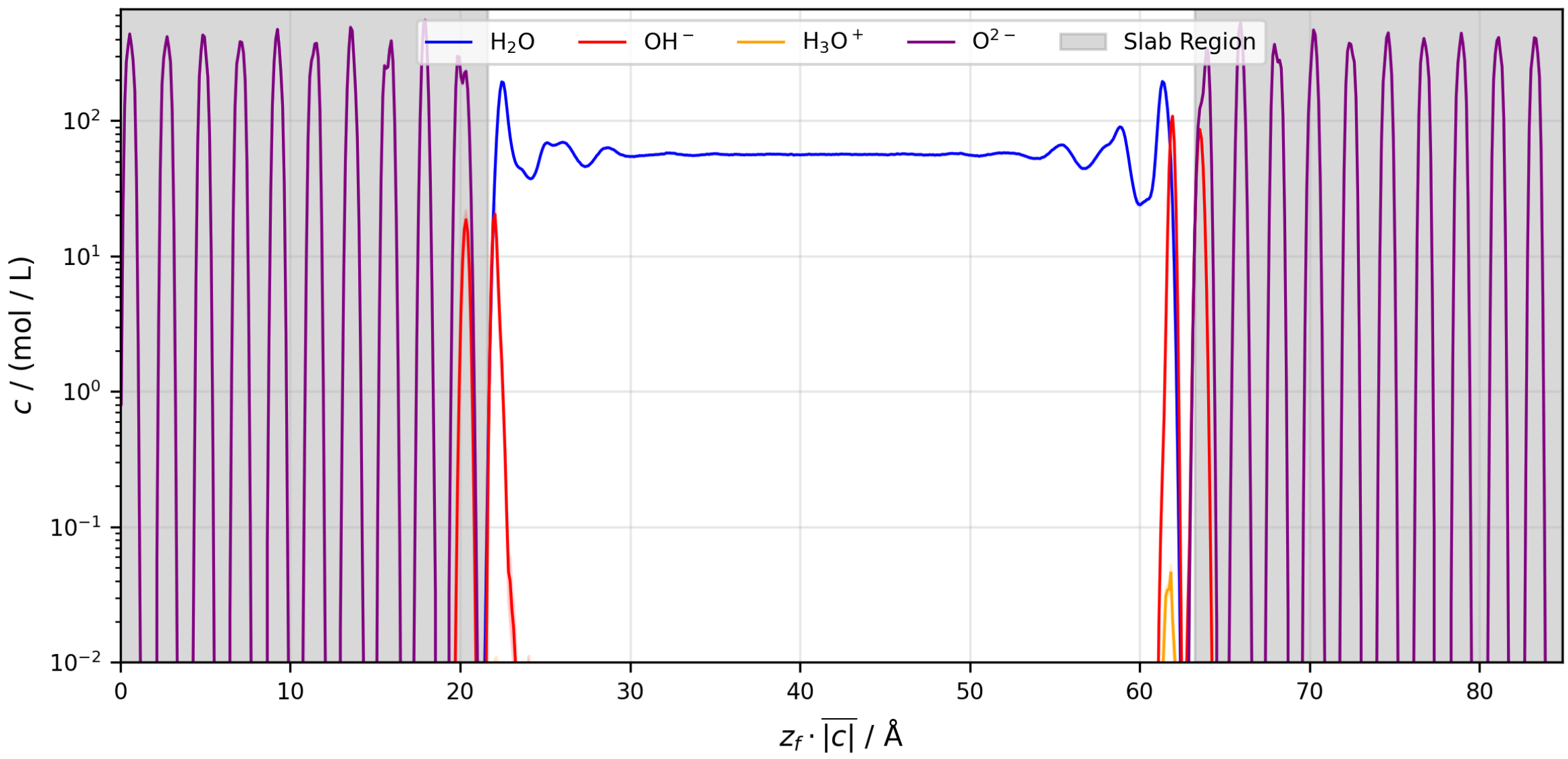

Figure S28: Concentration profiles of distinct oxygen-containing species along the [001] surface normal of the aqueous stoichiometric $LiMn_2O_4$ interface ($x_{Li} = 1.0$), averaged over the 3 ns production simulation. The shaded regions around the density lines represent the standard error of the mean (SEM), calculated with Bessel's correction via block averaging the trajectory over three consecutive 1 ns windows. The individual species (H_2O, OH^-, H_3O^+, and unprotonated O^{2-}) were identified using a geometric distance criterion: for every O atom in the system, the number of adjacent H atoms within a cutoff radius of $r_{OH} \leq 1.25$ Å was counted. The spatial coordinate plotted for each species corresponds to its molecular center-of-mass (COM). The solid slab region is shaded in gray for improved visibility of the solid-liquid boundary. To decouple the spatial distributions from the cell volume fluctuations inherent to the NpT ensemble, the atomic positions at each timestep were transformed into fractional coordinates and subsequently rescaled using the time-averaged lattice parameters ($z_f \cdot |\bar{c}|$). Furthermore, to eliminate artificial smearing from minor COM drift, the entire system was re-centered at each timestep by anchoring the COM of the Mn sub-lattice. *Note:* Because the spatial coordinates in these concentration profiles are centered on the liquid phase, whereas the overall density profiles in Figure S24 were centered on the solid slab, the spatial representation is inverted: the left-hand interface in this figure corresponds to the right-hand interface in Figure S24, and vice versa.

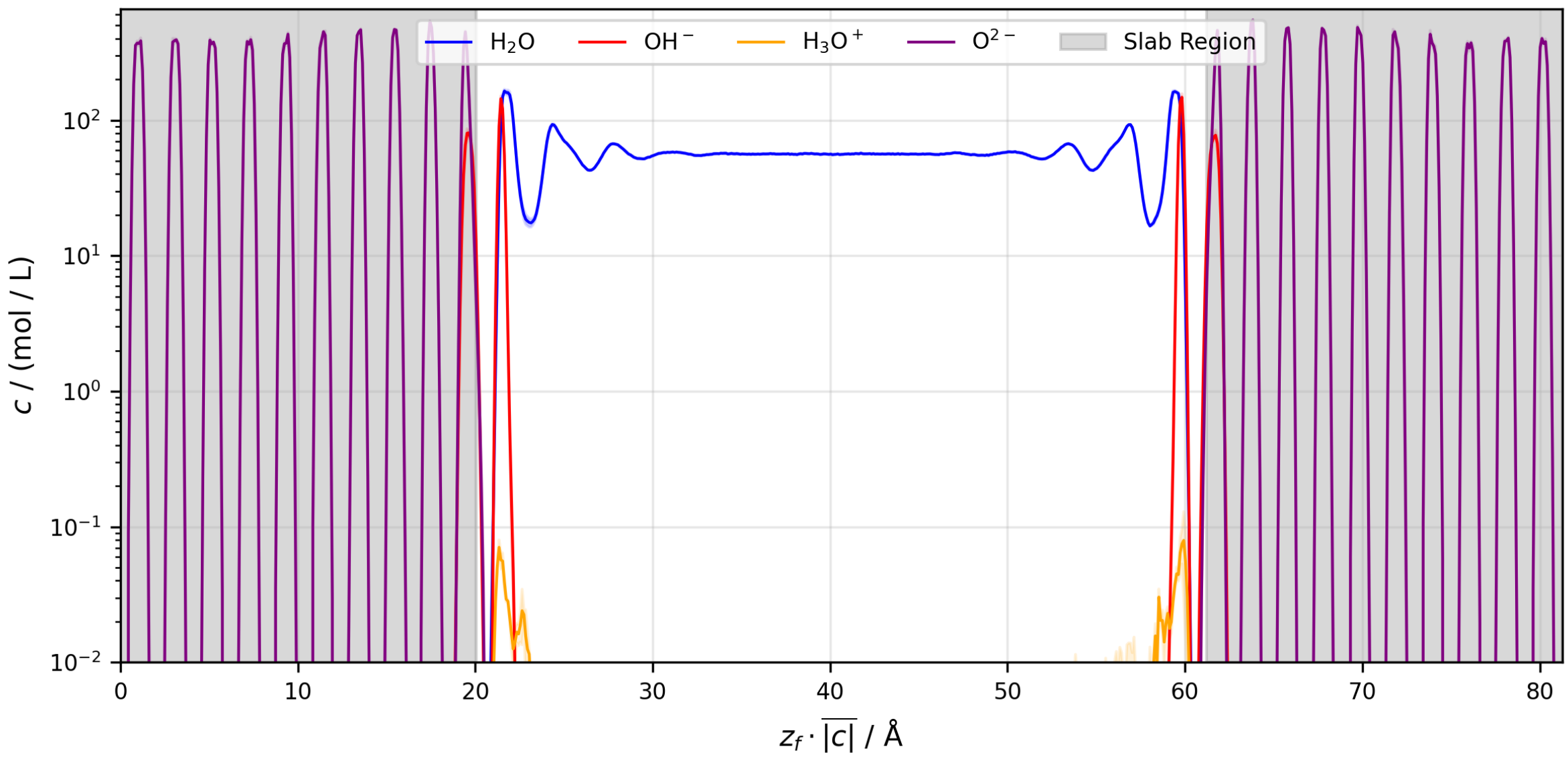

Figure S29: Concentration profiles of distinct oxygen-containing species along the [001] surface normal of the aqueous $Li^A_{0.5}Mn_2O_4$ interface ($x_{Li} = 0.5$), averaged over the 3 ns production simulation. The shaded regions around the density lines represent the standard error of the mean (SEM), calculated with Bessel's correction via block averaging the trajectory over three consecutive 1 ns windows. The individual species (H_2O, OH^-, H_3O^+, and unprotonated O^{2-}) were identified using a geometric distance criterion: for every O atom in the system, the number of adjacent H atoms within a cutoff radius of $r_{OH} \leq 1.25$ Å was counted. The spatial coordinate plotted for each species corresponds to its molecular center-of-mass (COM). The solid slab region is shaded in gray for improved visibility of the solid-liquid boundary. To decouple the spatial distributions from the cell volume fluctuations inherent to the NpT ensemble, the atomic positions at each timestep were transformed into fractional coordinates and subsequently rescaled using the time-averaged lattice parameters ($z_f \cdot |\bar{c}|$). Furthermore, to eliminate artificial smearing from minor COM drift, the entire system was re-centered at each timestep by anchoring the COM of the Mn sub-lattice. *Note:* Because the spatial coordinates in these concentration profiles are centered on the liquid phase, whereas the overall density profiles in Figure S25 were centered on the solid slab, the spatial representation is inverted: the left-hand interface in this figure corresponds to the right-hand interface in Figure S25, and vice versa.

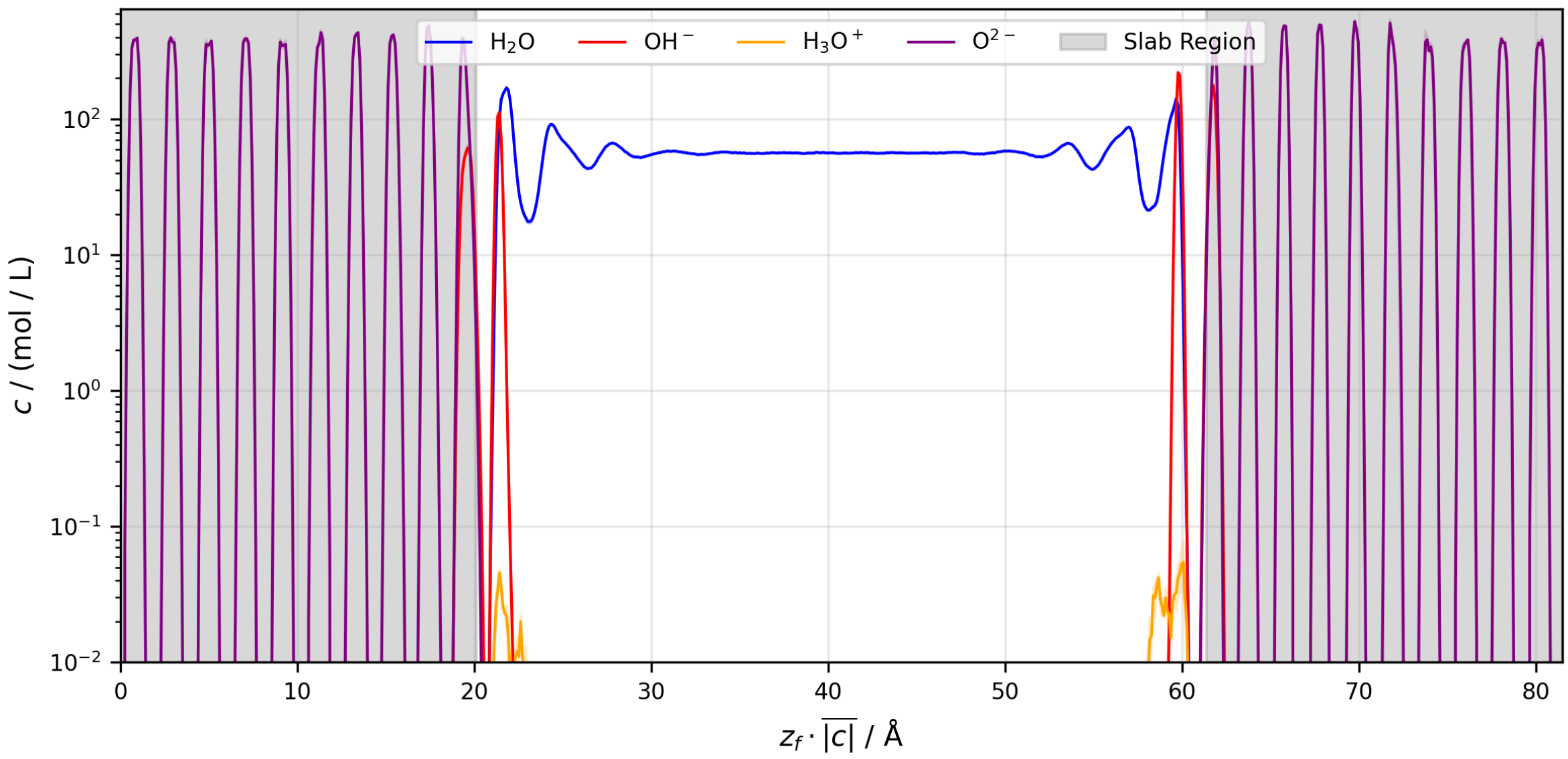

Figure S30: Concentration profiles of distinct oxygen-containing species along the [001] surface normal of the aqueous $Li^B_{0.5}Mn_2O_4$ interface ($x_{Li} = 0.5$), averaged over the 3 ns production simulation. The shaded regions around the density lines represent the standard error of the mean (SEM), calculated with Bessel's correction via block averaging the trajectory over three consecutive 1 ns windows. The individual species (H_2O, OH^-, H_3O^+, and unprotonated O^{2-}) were identified using a geometric distance criterion: for every O atom in the system, the number of adjacent H atoms within a cutoff radius of $r_{OH} \leq 1.25$ Å was counted. The spatial coordinate plotted for each species corresponds to its molecular center-of-mass (COM). The solid slab region is shaded in gray for improved visibility of the solid-liquid boundary. To decouple the spatial distributions from the cell volume fluctuations inherent to the NpT ensemble, the atomic positions at each timestep were transformed into fractional coordinates and subsequently rescaled using the time-averaged lattice parameters ($z_f \cdot |\bar{c}|$). Furthermore, to eliminate artificial smearing from minor COM drift, the entire system was re-centered at each timestep by anchoring the COM of the Mn sub-lattice. *Note:* Because the spatial coordinates in these concentration profiles are centered on the liquid phase, whereas the overall density profiles in Figure S26 were centered on the solid slab, the spatial representation is inverted: the left-hand interface in this figure corresponds to the right-hand interface in Figure S26, and vice versa.

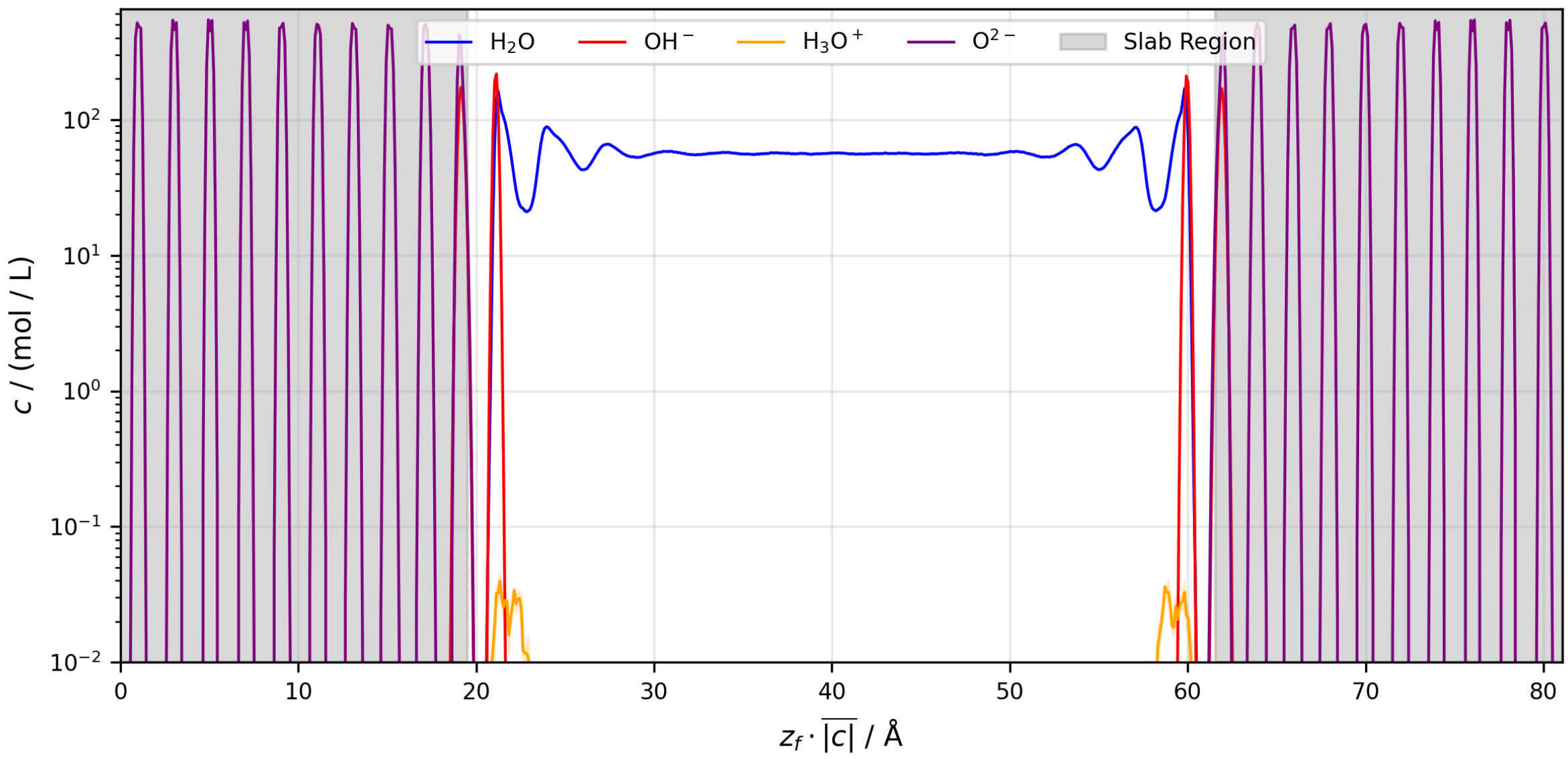

Figure S31: Concentration profiles of distinct oxygen-containing species along the [001] surface normal of the aqueous λ-MnO$_2$ interface ($x_{\mathrm{Li}} = 0.0$), averaged over the 3 ns production simulation. The shaded regions around the density lines represent the standard error of the mean (SEM), calculated with Bessel's correction via block averaging the trajectory over three consecutive 1 ns windows. The individual species (H_2O, OH^-, H_3O^+, and unprotonated O^{2-}) were identified using a geometric distance criterion: for every O atom in the system, the number of adjacent H atoms within a cutoff radius of $r_{\mathrm{OH}} \leq 1.25$ Å was counted. The spatial coordinate plotted for each species corresponds to its molecular center-of-mass (COM). The solid slab region is shaded in gray for improved visibility of the solid-liquid boundary. To decouple the spatial distributions from the cell volume fluctuations inherent to the NpT ensemble, the atomic positions at each timestep were transformed into fractional coordinates and subsequently rescaled using the time-averaged lattice parameters ($z_f \cdot |\bar{c}|$). Furthermore, to eliminate artificial smearing from minor COM drift, the entire system was re-centered at each timestep by anchoring the COM of the Mn sub-lattice. *Note:* Because the spatial coordinates in these concentration profiles are centered on the liquid phase, whereas the overall density profiles in Figure S27 were centered on the solid slab, the spatial representation is inverted: the left-hand interface in this figure corresponds to the right-hand interface in Figure S27, and vice versa.

both facets, which effectively eliminates unphysical cross-slab interactions. While it is physically possible for water molecules to traverse the periodic boundary separating the two bulk liquid regions, the relatively short simulation time of 20 ps renders such crossing events extremely rare, generally restricting them to molecules originating exactly at the boundary. To ensure a clean signal and avoid introducing a statistically negligible background-background exchange term, these crossing molecules were excluded from the VDOS analysis. Because our primary focus is isolating the distinct interfacial contributions, the omission of these transient background species has no impact on our physical conclusions.

S2.8.2 Interface and Bulk Background Contributions

The spectra presented in the main text exclude the bulk liquid background to focus on the contributions of the interfacial species. Here, we present the VDOS decomposed into bulk background and interface contributions (along with the exchange between them) for the various aqueous LMO interfaces as a function of lithiation state (Figures S32 and S33). As expected, the background signal is bulk-like and isotropic, featuring identical spectral weights in the x, y, and z directions, and is in good agreement with the bulk water spectrum presented in Figure S11. Naturally, the background intensity vastly exceeds the interface signal due to the significantly higher concentration of bulk species.

Interestingly, the OH-stretch region of the total VDOS (the sum of the interface, background, and cross terms) still exhibits a slight anisotropy in the z-direction at high surface oxidation states (cf. Figure S32d). This residual anisotropy arises from the strong spectral intensity of the surface hydroxide ($O_{surf}H$) species and the specific orientation of water molecules confined within the [110] channels. These confined water molecules form very weak hydrogen bonds with surface oxygen sites, which exhibit significantly reduced Lewis basicity because they are electron-starved by the highly oxidized Mn centers.

Finally, this decomposition physically illustrates the inherent challenge that standard, bulk-averaging experimental techniques face when attempting to resolve interfacial dynamics. It underscores the vital importance of surface- and interface-sensitive techniques, which are uniquely capable of isolating these interfacial signatures from the overwhelming bulk background noise.

S2.8.3 Remaining Chemical Species Decomposed Spectra

The main text focuses on the species-decomposed VDOS of the $LiMn_2O_4(001)$, $LiMn_2O_4(00\bar{1})$, $Li^A_{0.5}Mn_2O_4(001)$, and λ-$MnO_2(001)$ facets. The remaining spectra ($Li^B_{0.5}Mn_2O_4(001)$, $Li^A_{0.5}Mn_2O_4(00\bar{1})$, $Li^B_{0.5}Mn_2O_4(00\bar{1})$, and λ-$MnO_2(00\bar{1})$) are presented in Figure S34, ordered by increasing average surface oxidation state.

As detailed in Section S2.3, the $Li^B_{0.5}Mn_2O_4(001)$ and $Li^A_{0.5}Mn_2O_4(00\bar{1})$ facets feature mixed-valence Mn populations; specifically, they comprise approximately 40% hs-Mn^{3+} (60% Mn^{4+}) and 30% hs-Mn^{3+} (70% Mn^{4+}), respectively. This similarity results in nearly identical probability distributions for the species within the first hydration layer and, consequently, highly comparable spectral densities. We observe only a slight increase in the intensity of dissociation products for the $Li^A_{0.5}Mn_2O_4(00\bar{1})$ interface, which is consistent with its higher average surface Mn oxidation state. The remaining $Li^B_{0.5}Mn_2O_4(00\bar{1})$ and λ-$MnO_2(00\bar{1})$ facets are characterized by an exclusive Mn^{4+} surface population due to the complete absence of interfacial Li ions. Accordingly, these facets exhibit vibrational signatures that are near identical to each other and consistent with the λ-$MnO_2(001)$ interface discussed in the main text.

S2.8.4 Remaining Orientation Decomposed Spectra

The main text focuses on the orientation-decomposed VDOS of the $LiMn_2O_4(001)$, $LiMn_2O_4(00\bar{1})$, $Li^A_{0.5}Mn_2O_4(001)$, and λ-$MnO_2(001)$ facets. The remaining spectra ($Li^B_{0.5}Mn_2O_4(001)$,

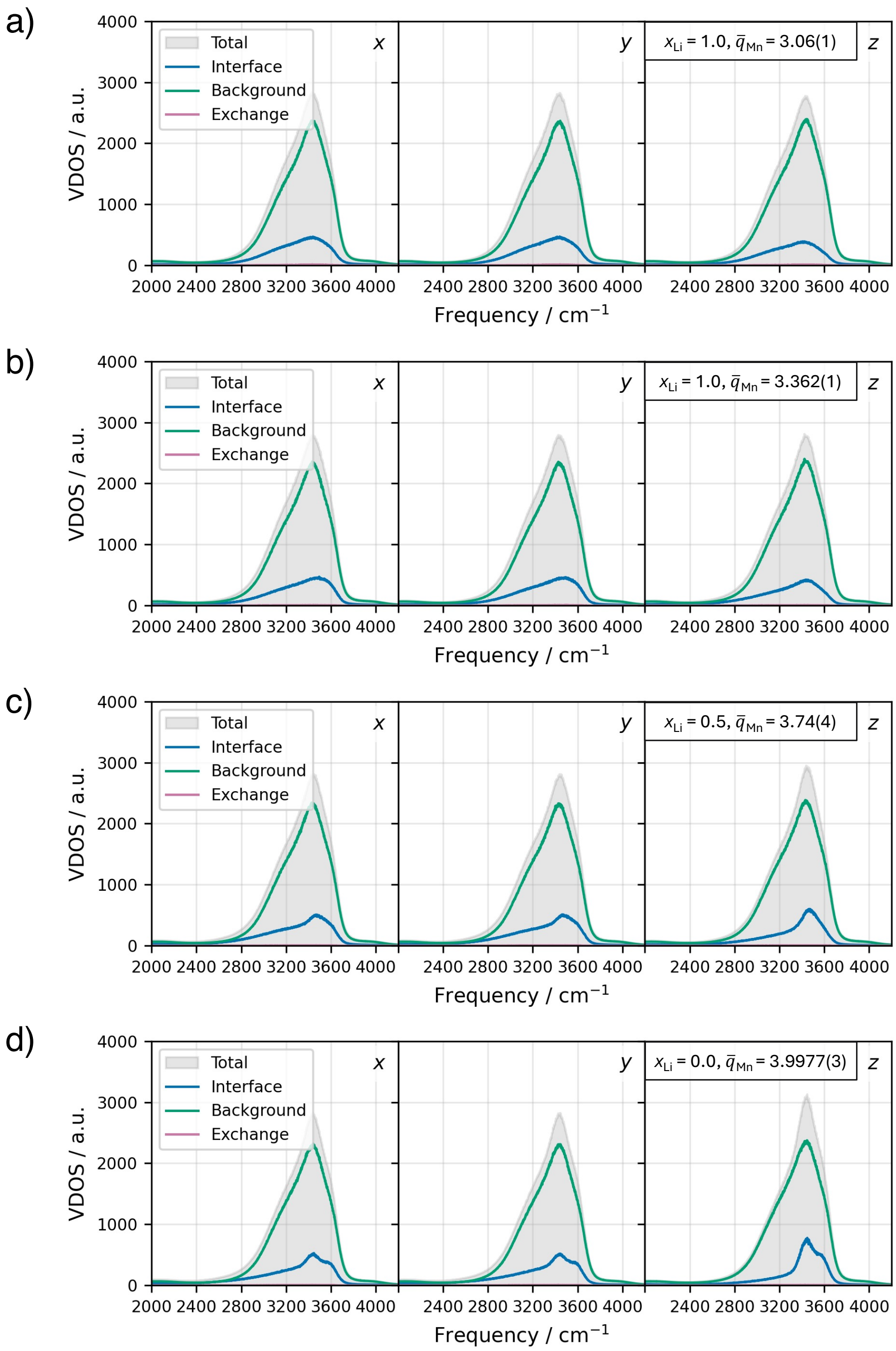

Figure S32: Decomposition of the H-atom vibrational density of states (VDOS) into interface and background (bulk) contributions. The exchange term represents the sum of the cross-correlation components between these two spatial regions. The shaded area corresponds to the total H-atom VDOS of the liquid phase adjacent to the respective surface facet. Each row presents a different surface structure, ordered by decreasing Li content from top to bottom: **a)** $Li^{B}_{0.5}Mn_2O_4(001)$, **b)** $Li^{A}_{0.5}Mn_2O_4(00\bar{1})$, **c)** $Li^{B}_{0.5}Mn_2O_4(00\bar{1})$, and **d)** λ-$MnO_2(00\bar{1})$.

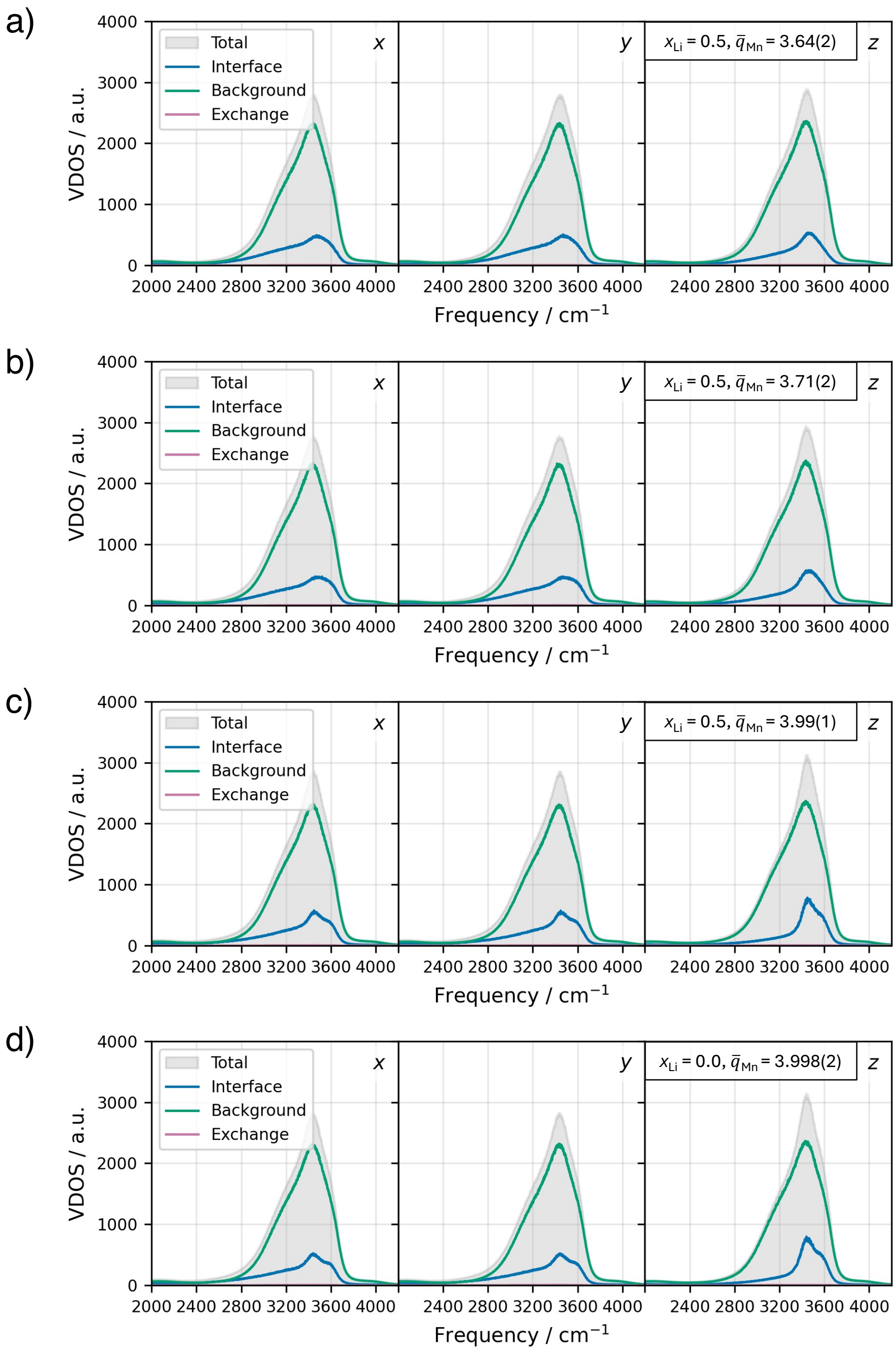

Figure S33: Decomposition of the H-atom vibrational density of states (VDOS) into interface and background (bulk) contributions. The exchange term represents the sum of the cross-correlation components between these two spatial regions. The shaded area corresponds to the total H-atom VDOS of the liquid phase adjacent to the respective surface facet. Each row presents a different surface structure, ordered by decreasing average surface oxidation state from top to bottom: **a)** $Li^{B}_{0.5}Mn_2O_4(001)$, **b)** $Li^{A}_{0.5}Mn_2O_4(00\bar{1})$, **c)** $Li^{B}_{0.5}Mn_2O_4(00\bar{1})$, and **d)** λ-$MnO_2(00\bar{1})$.

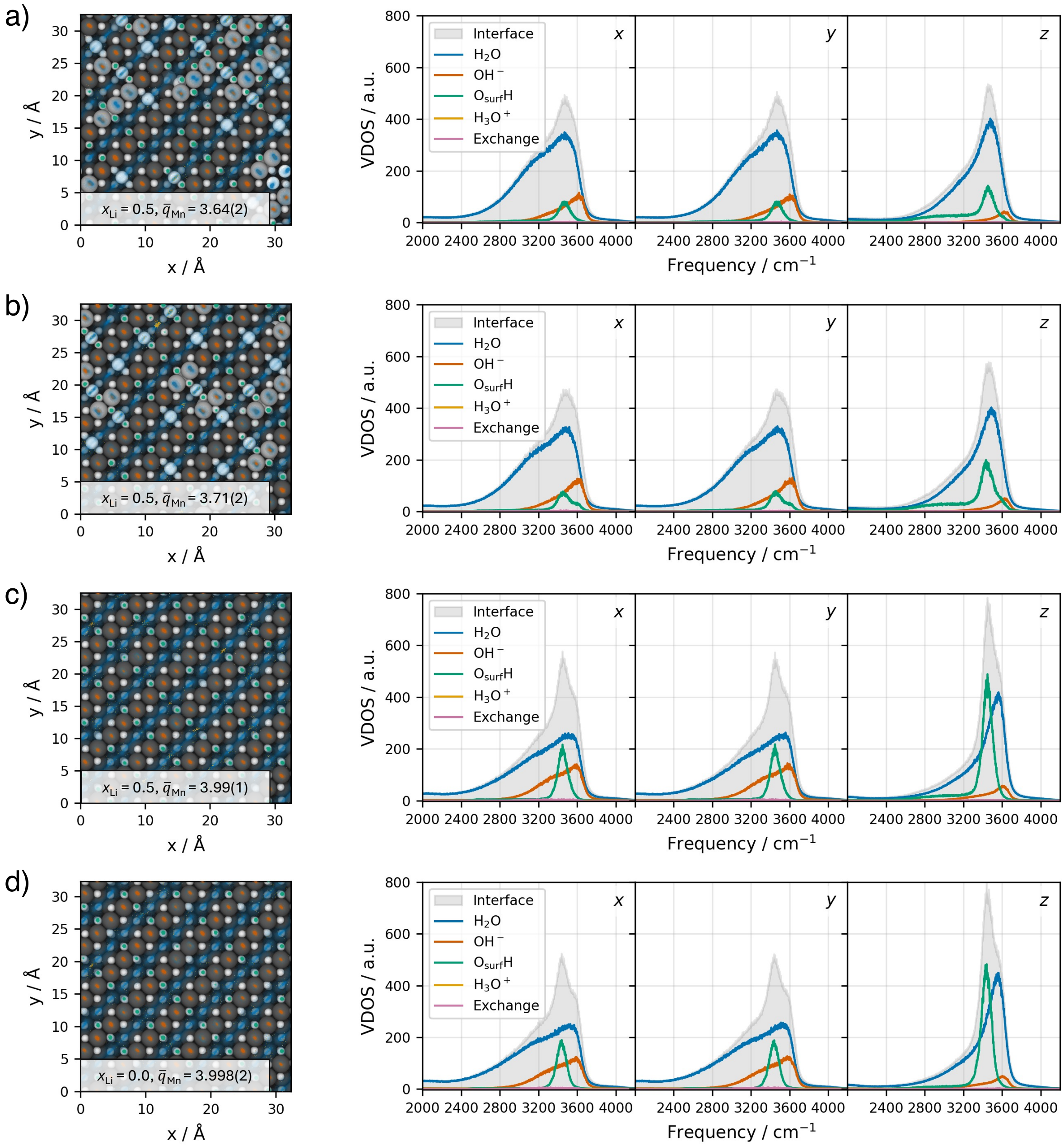

Figure S34: Decomposition of the VDOS into chemical species contributions. Each row represents a different surface structure with decreasing Li content from top to bottom: **a)** $Li^B_{0.5}Mn_2O_4(001)$, **b)** $Li^A_{0.5}Mn_2O_4(00\bar{1})$, **c)** $Li^B_{0.5}Mn_2O_4(00\bar{1})$, and **d)** λ-$MnO_2(00\bar{1})$. **Left:** Real-space probability distribution of chemical species within the first hydration layer. Species are color-coded as follows: water H_2O (blue), interfacial hydroxide OH^- (orange), protonated surface oxygen $O_{surf}H$ (bluish green), and hydronium H_3O^+ (yellow). The time-averaged surface atom positions are shown in grayscale: Mn^{4+} (dark gray, large radius), Mn^{3+} (gray, large radius), O (light gray, small radius), and Li (white, intermediate radius). The surfaces were rendered using OVITO Pro (version 3.14.1).[50] **Right:** Corresponding species decomposed spectral densities (VDOS / a.u.). The exchange term (purple) represents the sum of the cross-correlation components between all species. The gray background represents the overall VDOS of the interface region.

$Li^{A}_{0.5}Mn_2O_4(00\bar{1})$, $Li^{B}_{0.5}Mn_2O_4(00\bar{1})$, and λ-$MnO_2(00\bar{1})$) are presented in Figure S35, organized by increasing surface oxidation state.

As detailed in Section S2.3, the $Li^{B}_{0.5}Mn_2O_4(001)$ and $Li^{A}_{0.5}Mn_2O_4(00\bar{1})$ facets feature mixed-valence Mn populations; specifically, they comprise approximately 40% hs–Mn^{3+} (60% Mn^{4+}) and 30% hs–Mn^{3+} (70% Mn^{4+}), respectively. This chemical similarity results in nearly identical probability distributions for water orientation within the first hydration layer and, consequently, highly comparable spectral densities.

For the $Li^{A}_{0.5}Mn_2O_4(00\bar{1})$ interface, we observe only a slight blueshift for *toward*-oriented (H-bond donating) water molecules and a slight redshift for *away*-facing (Mn-coordinated) water. This is consistent with its higher average surface Mn oxidation state, which simultaneously reduces the Lewis basicity of surface oxygen sites in the [110] channels and increases the Lewis acidity of the surface Mn sites. These effects lead to reduced H-bond strength for the *toward* population and stronger polarization of the O–H bond by the metal center for the *away* population. The remaining $Li^{B}_{0.5}Mn_2O_4(00\bar{1})$ and λ-$MnO_2(00\bar{1})$ facets are characterized by an exclusive Mn^{4+} surface population due to the complete absence of interfacial Li ions. Accordingly, these facets exhibit vibrational signatures that are nearly identical to each other and consistent with the λ-$MnO_2(001)$ interface discussed in the main text.

S2.9 Thermodynamics of Water Dissociation

To relate the identified vibrational frequency shifts to the thermodynamic driving forces of water dissociation, we determined the Potentials of Mean Force (PMFs) from the NpT trajectories of the LMO–water interfaces:

$$G(\xi) = -k_{\mathrm{B}}T \ln P(\xi), \tag{S4}$$

where G is the Gibbs free energy, k_{B} is Boltzmann's constant, $T = 350\,\mathrm{K}$ is the simulation temperature (recovering the dynamics of water under ambient conditions; Section S1.8), and $P(\xi)$ is the probability density along the chosen reaction coordinate ξ.[54, 55]

S2.9.1 Definition of Reaction Coordinates

To map the thermodynamics of water dissociation, we defined three key reaction coordinates. First, water adsorption is described by the distance along the surface normal (r_z^{MnO}) between a surface Mn site and the nearest oxygen center in the first water layer. To prevent artifacts from neighboring active sites, the nearest oxygen center search was restricted to a cylinder with a 1.5 Å radius and 8 Å height laterally centered around each Mn site.

Second, to track the cleavage of the O–H bond, we employed the well-established proton displacement coordinate $\delta = r_{\mathrm{OH}} - r_{\mathrm{O_{surf}H}}$.[56] Here, r_{OH} and $r_{\mathrm{O_{surf}H}}$ are the 3D distances from the transferring proton to the adsorbed oxygen center and to an equatorial surface oxygen (O_{surf}) of the Mn site, respectively. To accurately capture the dissociation event, the active proton and its specific accepting O_{surf} site were dynamically assigned depending on the state of the adsorbed species:

- **For an intact adsorbed water molecule** (defined by the same geometric distance criterion, $r_{\mathrm{OH}} \leq 1.25$ Å, used for the VDOS species decomposition): The algorithm evaluates the eight possible pairs between the two water protons and the four local equatorial oxygen sites. The pair that maximizes δ (i.e., the one furthest along the reaction coordinate) is selected.

- **For an adsorbed hydroxide ion** (only one proton within 1.25 Å): The active proton is identified via a three-step geometric criterion. First, candidate protons are selected within a 4 Å sphere around the adsorbed oxygen center (excluding the proton already bound to the adsorbed oxygen center). Second, for each of the four equatorial O_{surf} sites,

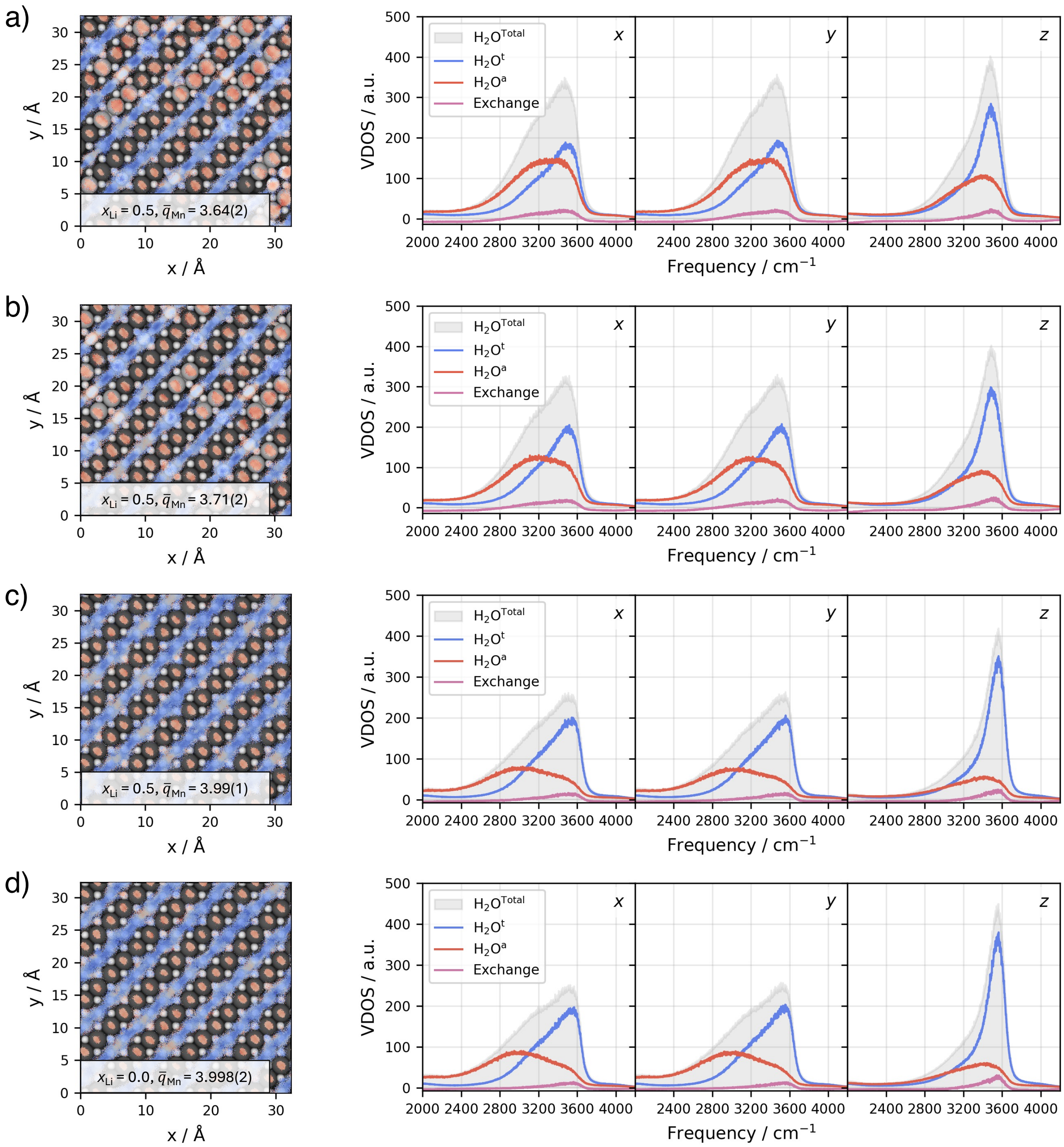

Figure S35: Correlation between interfacial water orientation and vibrational dynamics. Each row represents a different surface structure with decreasing Li content from top to bottom: **a)** $Li^{B}_{0.5}Mn_2O_4(001)$, **b)** $Li^{A}_{0.5}Mn_2O_4(00\bar{1})$, **c)** $Li^{B}_{0.5}Mn_2O_4(00\bar{1})$, and **d)** λ-$MnO_2(00\bar{1})$. **Left:** Real-space probability distribution of first layer water molecules, color-coded by molecular orientation. The orientation is defined by the angle θ between the water bisector and the surface normal, represented by a diverging color gradient from blue ($\cos\theta = -1$, pointing toward the surface) to red ($\cos\theta = 1$, pointing away), with white indicating a surface-parallel (flat) orientation. The time-averaged surface atom positions are shown in grayscale: Mn^{4+} (dark gray, large radius), Mn^{3+} (gray, large radius), O (light gray, small radius), and Li (white, intermediate radius). The surfaces were rendered using OVITO Pro (version 3.14.1).[50] **Right:** Corresponding water species spectral density (VDOS / a.u.) decomposed by the same orientation criteria. The exchange term (purple) represents the sum of the cross-correlation components between the two orientations.

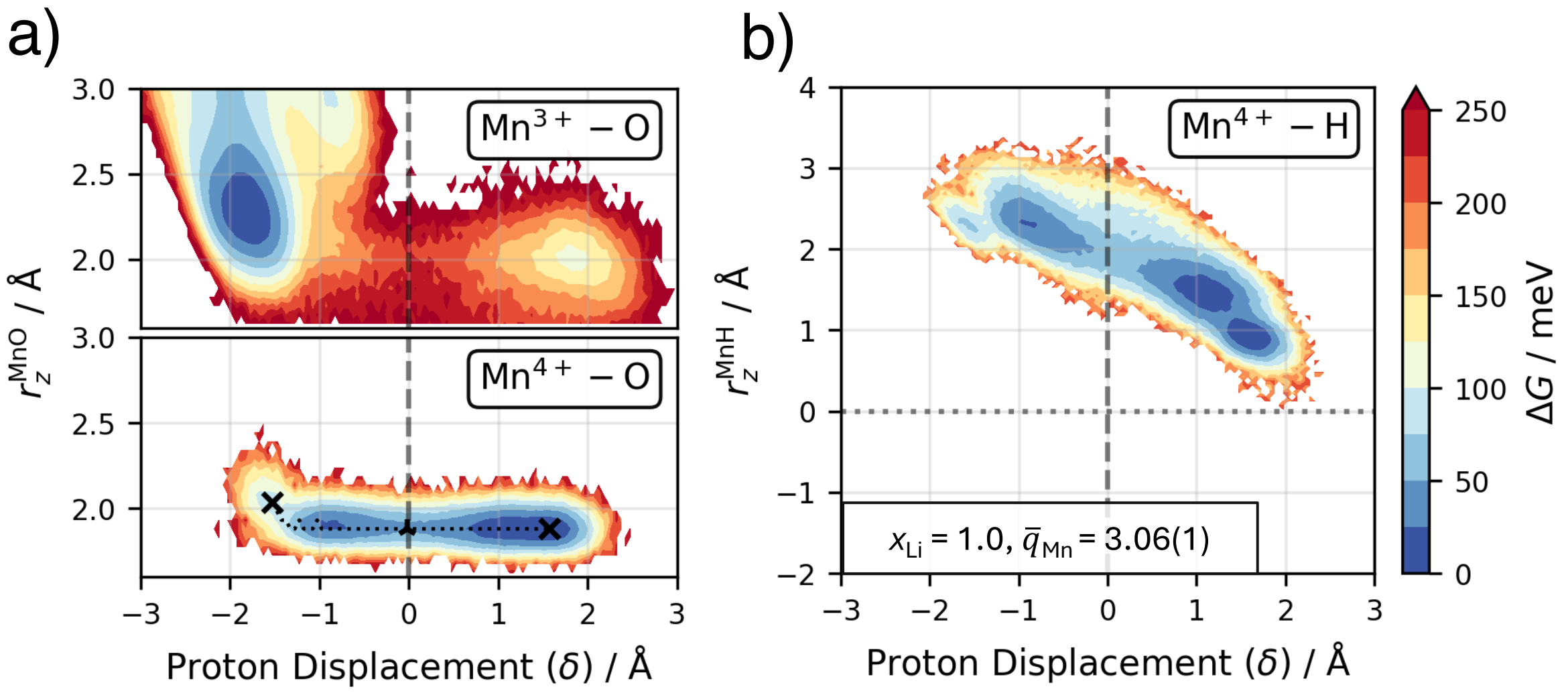

Figure S36: a) Gibbs free energy surfaces (PMFs) of the aqueous $LiMn_2O_4(00\bar{1})$ interface for water dissociation at both Mn^{3+} and Mn^{4+} sites, projected onto the Mn–O distance along the surface normal (r_z^{MnO}) and the proton displacement coordinate ($\delta = r_{OH} - r_{O_{surf}H}$). Within the Mn^{4+} sub-panels, dotted lines trace the minimum energy path, with reactant and product minima and transition states marked by crosses and upward-pointing triangles, respectively. **b)** PMFs detailing the free energy landscape of the active proton at Mn^{4+} sites as a function of the Mn–H surface-normal distance (r_z^{MnH}) and δ.

the proton that minimizes the sum of the distances ($r_{OH} + r_{O_{surf}H}$) across all candidate protons—representing the shortest bridging path between the surface and the adsorbed oxygen site—is selected as the candidate active proton. Finally, among these four resulting shortest paths, the specific proton–O_{surf} pair that maximizes δ is selected.

Third, having identified the active proton, the final reaction coordinate is determined as the distance along the surface normal (r_z^{MnH}) between the surface Mn site and the active proton.

S2.9.2 Remaining Potentials of Mean Force

The main text focuses on the PMFs of the $LiMn_2O_4(00\bar{1})$ and $Li^A_{0.5}Mn_2O_4(001)$ interfaces. The remaining PMFs ($LiMn_2O_4(001)$, $Li^B_{0.5}Mn_2O_4(001)$, $Li^A_{0.5}Mn_2O_4(00\bar{1})$, $Li^B_{0.5}Mn_2O_4(00\bar{1})$, λ-$MnO_2(001)$, and λ-$MnO_2(00\bar{1})$) are presented in Figures S36–S41, organized in order of increasing surface oxidation state.

The PMFs of these progressively more oxidized surfaces clearly illustrate the trends discussed in the main text: ΔG_{diss} increases with the average surface oxidation state, reaching a maximum for the fully delithiated λ-MnO_2 interfaces due to the systematically reduced Lewis basicity of the surface oxygen sites. Crucially, these highly oxidized surfaces exhibit the most pronounced thermodynamic stabilization via surface hydroxyl rotation. This local structural relaxation overcomes the initial endergonic dissociation step, ultimately resulting in a net exergonic reaction.

S2.10 Effect of Local Li Content and Vacancies

We modeled four LMO–water interface systems representing three macroscopic lithiation states ($x_{Li} = 1.0, 0.5$, and 0.0): the stoichiometric $LiMn_2O_4$ interface ($x_{Li} = 1.0$), two half-lithiated interfaces with distinct initial lithium distributions ($Li^A_{0.5}Mn_2O_4$ and $Li^B_{0.5}Mn_2O_4$, $x_{Li} = 0.5$), and the fully delithiated λ-Mn_2O_4 interface ($x_{Li} = 0.0$). Although these systems span three macroscopic states, their local interfacial Li concentrations are more diverse due to varying surface terminations and initial Li ion distributions. Furthermore, while bulk Li diffusion is rare under

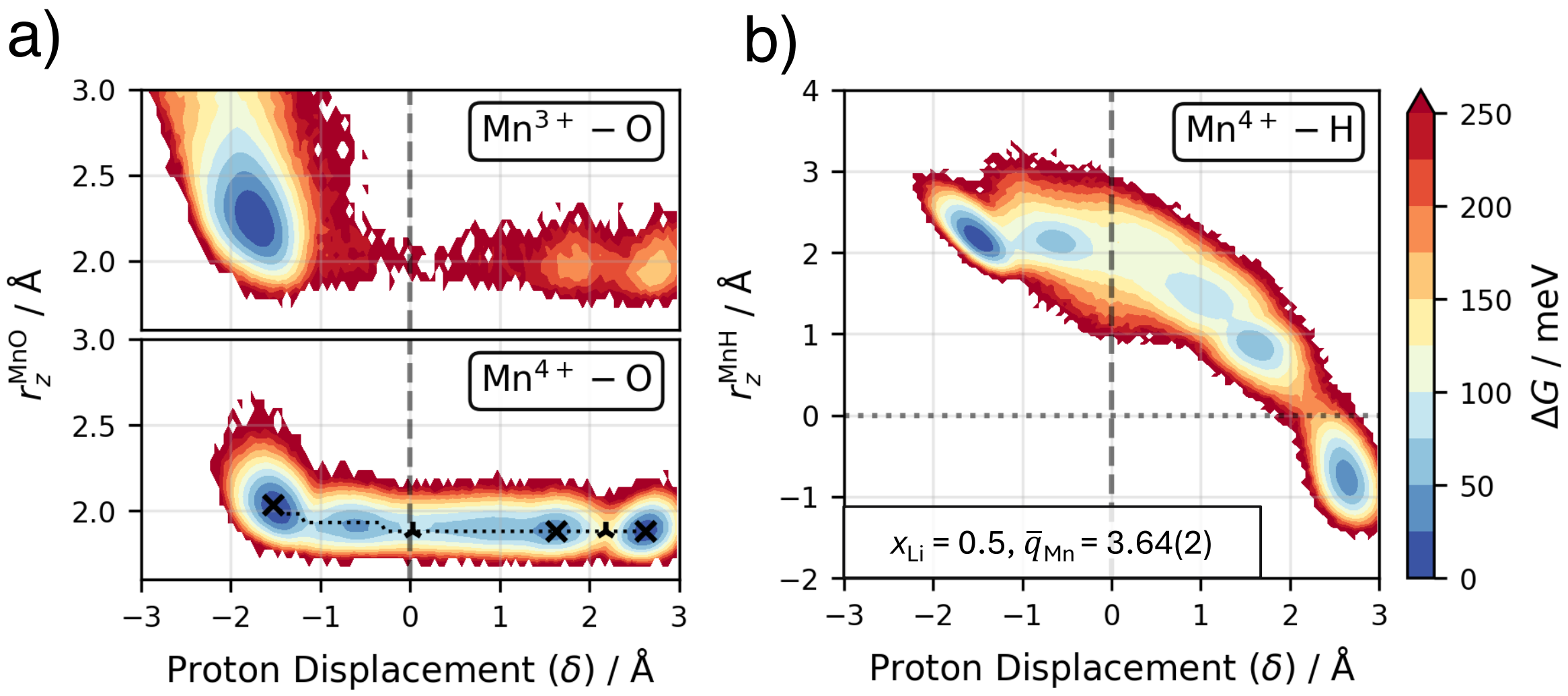

Figure S37: a) Gibbs free energy surfaces (PMFs) of the aqueous $Li^{B}_{0.5}Mn_2O_4(001)$ interface for water dissociation at both Mn^{3+} and Mn^{4+} sites, projected onto the Mn–O distance along the surface normal (r_z^{MnO}) and the proton displacement coordinate ($\delta = r_{\mathrm{OH}} - r_{\mathrm{O_{surf}H}}$). Within the Mn^{4+} sub-panels, dotted lines trace the minimum energy path, with reactant and product minima and transition states marked by crosses and upward-pointing triangles, respectively. **b)** PMFs detailing the free energy landscape of the active proton at Mn^{4+} sites as a function of the Mn–H surface-normal distance (r_z^{MnH}) and δ.

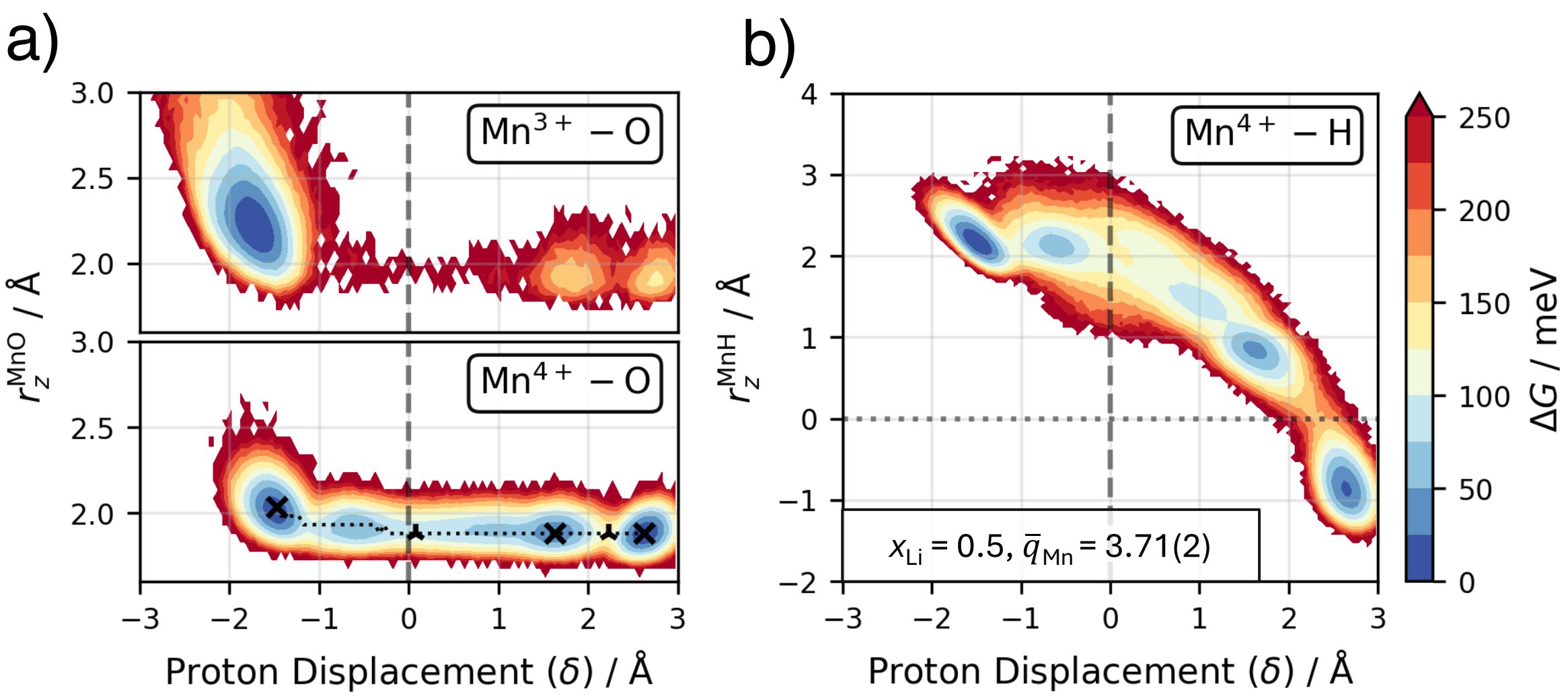

Figure S38: a) Gibbs free energy surfaces (PMFs) of the aqueous $Li^{A}_{0.5}Mn_2O_4(00\bar{1})$ interface for water dissociation at both Mn^{3+} and Mn^{4+} sites, projected onto the Mn–O distance along the surface normal (r_z^{MnO}) and the proton displacement coordinate ($\delta = r_{\mathrm{OH}} - r_{\mathrm{O_{surf}H}}$). Within the Mn^{4+} sub-panels, dotted lines trace the minimum energy path, with reactant and product minima and transition states marked by crosses and upward-pointing triangles, respectively. **b)** PMFs detailing the free energy landscape of the active proton at Mn^{4+} sites as a function of the Mn–H surface-normal distance (r_z^{MnH}) and δ.

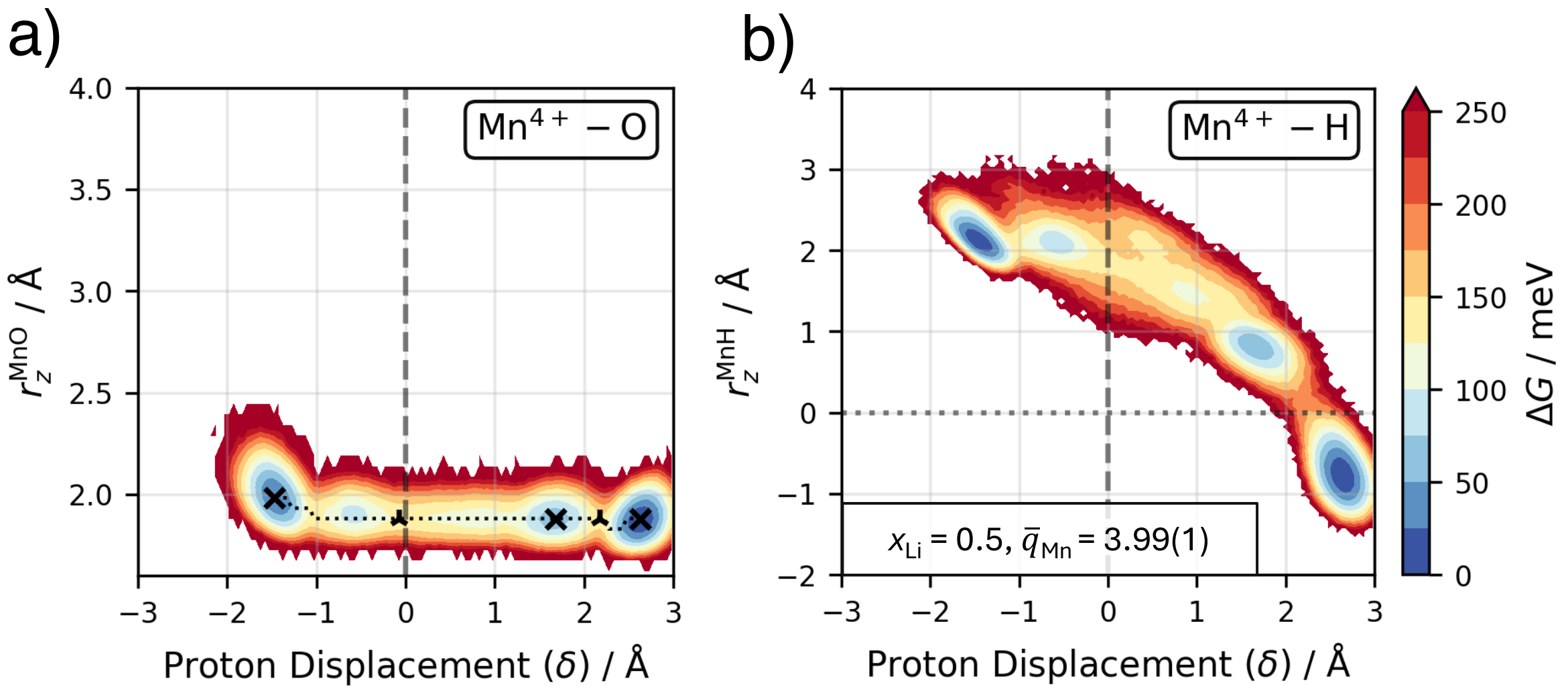

Figure S39: a) Gibbs free energy surfaces (PMFs) of the aqueous $Li^{B}_{0.5}Mn_2O_4(00\bar{1})$ interface for water dissociation at Mn^{4+} sites, projected onto the Mn–O distance along the surface normal (r_z^{MnO}) and the proton displacement coordinate ($\delta = r_{OH} - r_{O_{surf}H}$). The dotted lines trace the minimum energy path, with reactant and product minima and transition states marked by crosses and upward-pointing triangles, respectively. **b)** PMFs detailing the free energy landscape of the active proton at Mn^{4+} sites as a function of the Mn–H surface-normal distance (r_z^{MnH}) and δ.

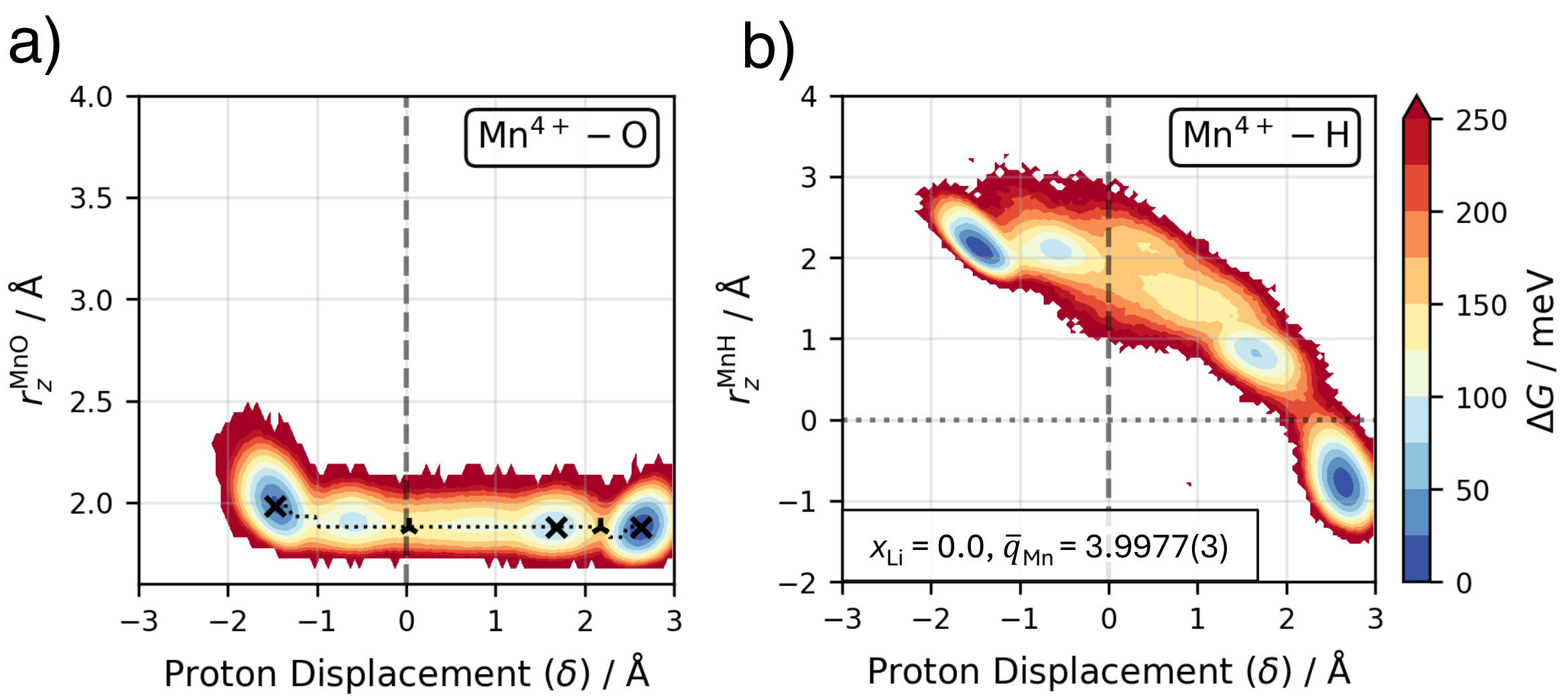

Figure S40: a) Gibbs free energy surfaces (PMFs) of the aqueous λ-$MnO_2(001)$ interface for water dissociation at Mn^{4+} sites, projected onto the Mn–O distance along the surface normal (r_z^{MnO}) and the proton displacement coordinate ($\delta = r_{OH} - r_{O_{surf}H}$). The dotted lines trace the minimum energy path, with reactant and product minima and transition states marked by crosses and upward-pointing triangles, respectively. **b)** PMFs detailing the free energy landscape of the active proton at Mn^{4+} sites as a function of the Mn–H surface-normal distance (r_z^{MnH}) and δ.

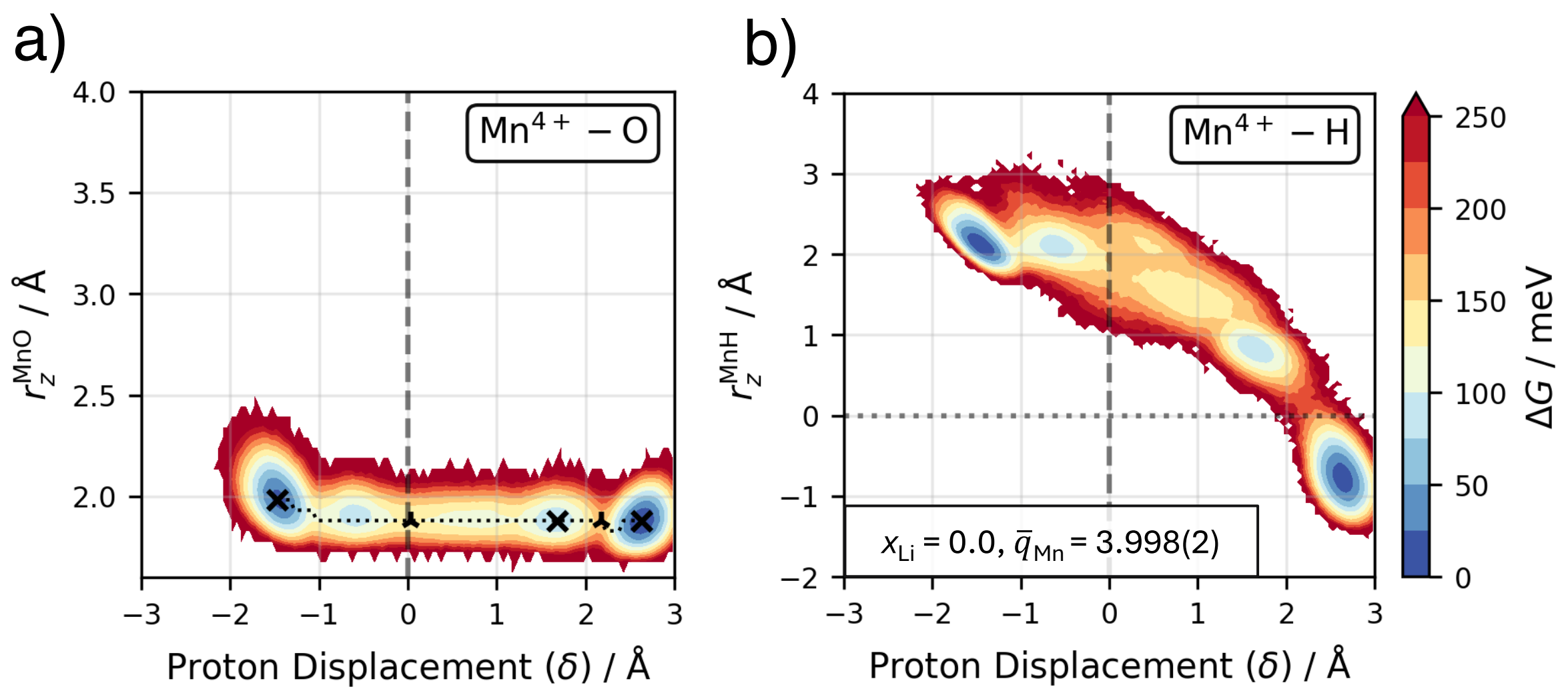

Figure S41: a) Gibbs free energy surfaces (PMFs) of the aqueous λ-$MnO_2(00\bar{1})$) interface for water dissociation at Mn^{4+} sites, projected onto the Mn–O distance along the surface normal (r_z^{MnO}) and the proton displacement coordinate ($\delta = r_{OH} - r_{O_{surf}H}$). The dotted lines trace the minimum energy path, with reactant and product minima and transition states marked by crosses and upward-pointing triangles, respectively. **b)** PMFs detailing the free energy landscape of the active proton at Mn^{4+} sites as a function of the Mn–H surface-normal distance (r_z^{MnH}) and δ.

our simulation conditions, the spatial distribution of Li ions evolved throughout the simulations, as evidenced by the density profiles in Section S2.6. Consequently, the MD trajectories sample a broader variance of local interfacial Li concentrations than suggested by the macroscopic lithiation states alone. In prior work, we established that the local Li arrangement directly dictates the specific distribution of Mn^{3+} and Mn^{4+} sites in the bulk,[7] a coupling that remains evident for these interfacial systems (see Section S2.3). To establish the local Li/vacancy concentrations at the interface, we performed a spatially resolved analysis at the solid–liquid boundary.

To achieve this, we analyzed the time-averaged lithium density profiles (detailed in Section S2.6) extracted from the initial 3 ns of our NpT trajectories. The total local Li content at the interface was quantified by integrating the Li density from the outermost surface layer down to the fourth Mn layer, corresponding to a depth of approximately one unit cell. Furthermore, to specifically isolate the sub-surface Li vacancy concentration, we evaluated the integrated Li density in between the first and second Mn layers. As demonstrated in the main text, these sub-surface vacancies are critical for the thermodynamic stabilization of surface hydroxyl groups; specifically, they enable a rotation of the O–H bond inward, directing the proton into the electron-rich vacancy. For clarity, these integration boundaries are exemplarily illustrated for the $Li^{A}_{0.5}Mn_2O_4$ system in Figure S42. The resulting local Li fractions (x_{Li}^{local}) and sub-surface Li vacancy fraction ($x_{V_{Li}}^{local}$) are summarized in Table S5.

S2.10.1 Correlation Between Local Li Content and Surface Mn Oxidation States

As shown in Table S5, the simulated interfaces exhibit six distinct local Li concentrations and five different sub-surface vacancy concentrations. To correlate these local Li environments with the average surface-layer Mn oxidation states ($\bar{q}_{Mn}$) discussed in the main text, we plot $\bar{q}_{Mn}$ as a function of x_{Li}^{local} in Figure S43. A clear linear relationship emerges between the two properties, confirming that the local Li content directly dictates the oxidation states of the surface Mn ions, which subsequently governs the interfacial acid–base chemistry. Furthermore, this robust linear correlation establishes a predictive framework, allowing one to estimate how intermediate, dynamically fluctuating local Li concentrations would affect the average surface oxidation states.

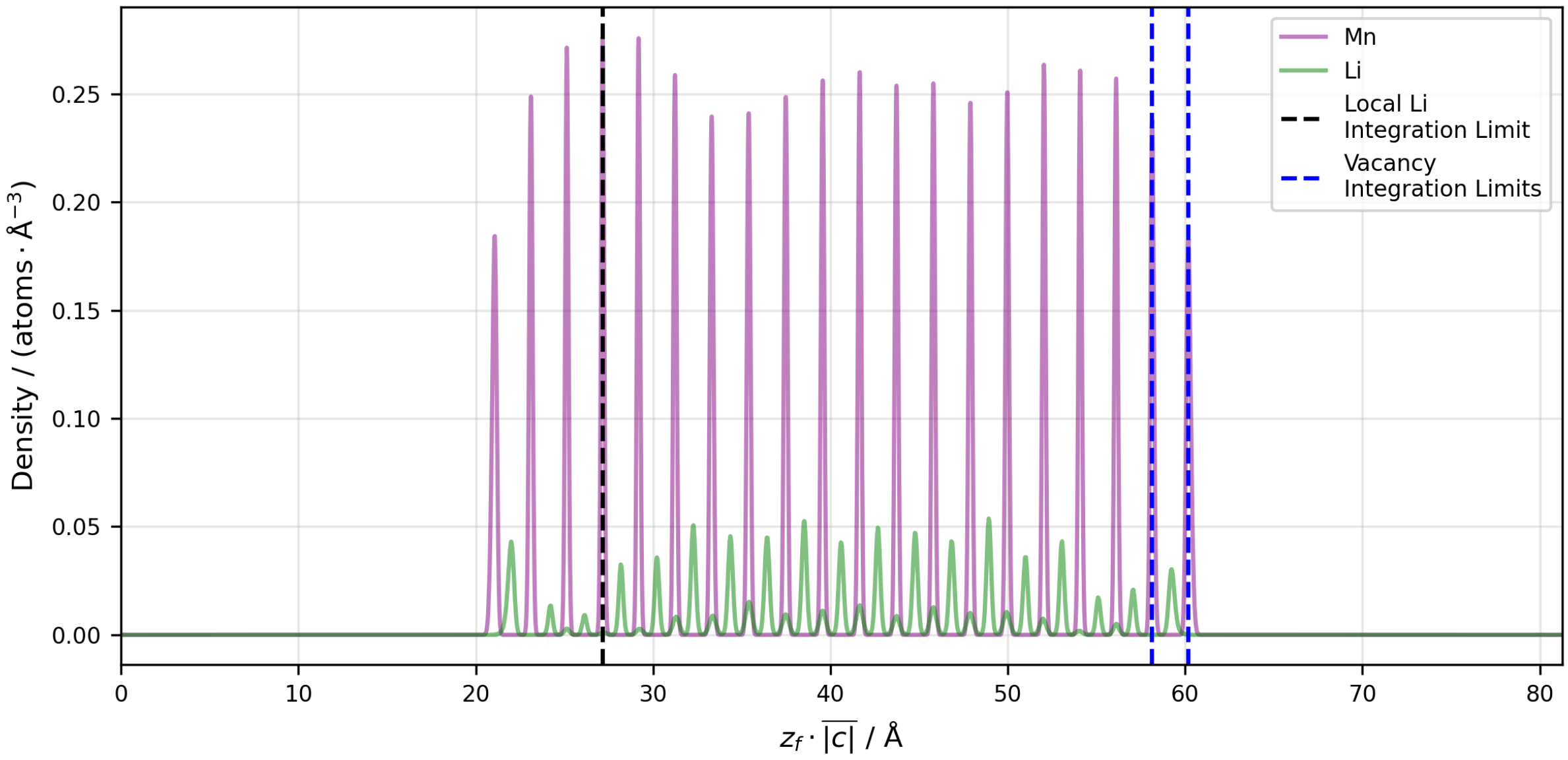

Figure S42: Element-specific density profile along the [001] surface normal of the aqueous $Li^{A}_{0.5}Mn_2O_4$ interface ($x_{Li} = 0.5$), averaged over the 3 ns production simulation. For clarity, only the Mn and Li densities are shown; the full profile is provided in Figure S25. Vertical dashed lines indicate the integration boundaries used to quantify local interfacial Li/vacancy concentrations, which were evaluated symmetrically for both sides of the slab. For visual clarity, only one set of boundaries is highlighted per interface: the black dashed line at the bottom interface denotes the local Li integration limit (extending from the outermost surface down to the fourth Mn peak, corresponding to a depth of approximately one unit cell), while the two blue dashed lines at the top interface denote the boundaries for the sub-surface vacancy concentration (spanning the first and second Mn peaks).

Table S5: Calculated local lithium fractions ($x_{\mathrm{Li}}^{\mathrm{local}}$) and sub-surface lithium vacancy fractions ($x_{V_{\mathrm{Li}}}^{\mathrm{local}}$) for the asymmetric top (001) and bottom (00$\bar{1}$) surfaces of the simulated LMO–water slab models. Values are obtained by integrating the time-averaged density profiles over the initial 3 ns of the production trajectories using the spatial boundaries detailed in the text.

Surface	$x_{\mathrm{Li}}^{\mathrm{local}}$	$x_{V_{\mathrm{Li}}}^{\mathrm{local}}$
$LiMn_2O_4(001)$	0.97	0.0
$LiMn_2O_4(00\bar{1})$	0.75	0.0
$Li^{A}_{0.5}Mn_2O_4(001)$	0.23	0.58
$Li^{A}_{0.5}Mn_2O_4(00\bar{1})$	0.22	0.39
$Li^{B}_{0.5}Mn_2O_4(001)$	0.35	0.44
$Li^{B}_{0.5}Mn_2O_4(00\bar{1})$	0.01	1.0
λ-$MnO_2(001)$	0.0	1.0
λ-$MnO_2(00\bar{1})$	0.0	1.0

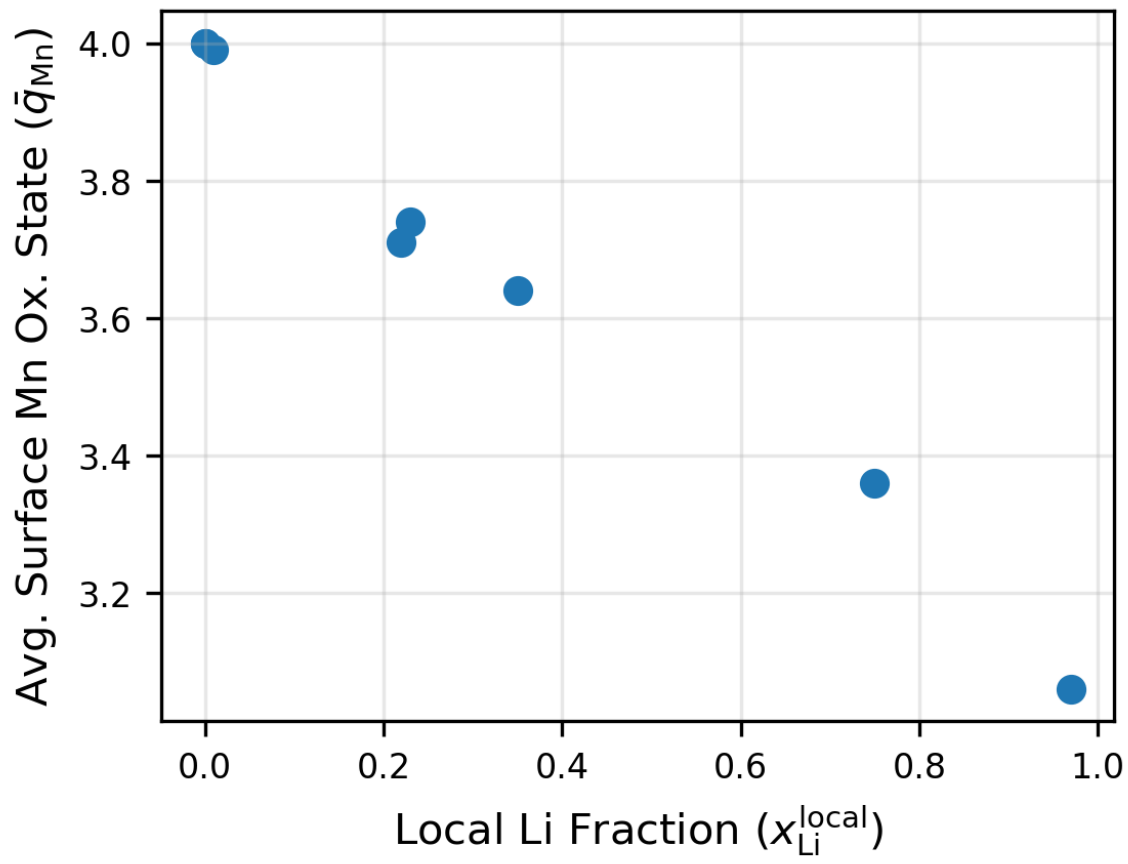

Figure S43: Correlation between local interfacial lithium concentration and surface manganese oxidation states. Average surface-layer Mn oxidation states ($\bar{q}_{\mathrm{Mn}}$) plotted as a function of the local interfacial Li fraction ($x_{\mathrm{Li}}^{\mathrm{local}}$) extracted from the simulated LMO–water interfaces.

S2.10.2 Correlation Between Sub-surface Li Vacancy Concentration and Proton Stabilization

In the thermodynamic analysis of water dissociation discussed in the main text, we identified two dominant stabilization pathways for the generated surface protons: (i) long-range delocalization via proton transfer from active Mn^{4+} sites toward more Lewis-basic Mn^{3+} environments, and (ii) local stabilization via the inward rotation of the surface O–H bond into a sub-surface Li vacancy. Because the proton displacement reaction coordinate (δ) only captures the immediate structural relaxation of the surface proton rather than global delocalization, the free energy differences ($\Delta G_{\mathrm{diss}}^{\mathrm{local}}$) derived from the PMFs represent upper bounds on the dissociation energy.

To quantify the extent of the thermodynamic stabilization, we determined a global free energy change ($\Delta G_{\mathrm{diss}}^{\mathrm{global}} = -k_{\mathrm{B}}T \ln \left(\langle N_{\mathrm{Mn^{4+}-OH^-}}\rangle / \langle N_{\mathrm{Mn^{4+}-H_2O}}\rangle\right)$) directly from the population ratio of adsorbed OH^- and H_2O at the Mn^{4+} sites. The magnitude of this stabilization should scale with the local Li vacancy concentration, as the availability of these sub-surface sites dictates whether and to what extent the local relaxation pathway is accessible. To verify this, we plotted the stabilization energy, defined as $\Delta G_{\mathrm{diss}}^{\mathrm{global}} - \Delta G_{\mathrm{diss}}^{\mathrm{local}}$, as a function of the sub-surface lithium vacancy fraction ($x_{V_{\mathrm{Li}}}^{\mathrm{local}}$) in Figure S44. Here, a clear converging trend emerges. We observe significant thermodynamic stabilization ($\Delta G_{\mathrm{diss}}^{\mathrm{global}} - \Delta G_{\mathrm{diss}}^{\mathrm{local}} \ll 0$) for surfaces lacking accessible sub-surface vacancies, whereas the stabilization approaches zero for surfaces with a vacancy fraction $x_{V_{\mathrm{Li}}}^{\mathrm{local}} = 1.0$. Interfaces with intermediate vacancy fractions exhibit intermediate stabilization energies. Consequently, the interplay between local and global stabilization mechanisms is governed by the accessibility of sub-surface vacancies.

Furthermore, it needs to be emphasized that these vacancies simultaneously modulate the average surface Mn oxidation states; notably, all interfaces with $x_{V_{\mathrm{Li}}}^{\mathrm{local}} = 1.0$ result in fully oxidized Mn^{4+} surfaces in our simulations. Therefore, the thermodynamics of water dissociation at the aqueous LMO interface is heavily dependent on both the local Li concentration and its specific spatial distribution at the solid–liquid boundary. The trends derived across several distinct local LMO–water configurations investigated here provide a broad, systematic overview of the expected changes in interfacial dynamics across the Li composition range ($0 \leq x_{\mathrm{Li}} \leq 1.0$). Nevertheless, capturing the full variability of surface Mn behavior arising from specific, short-range Li/vacancy ordering configurations and its effect on interfacial dynamics represents a highly promising avenue for future investigation.

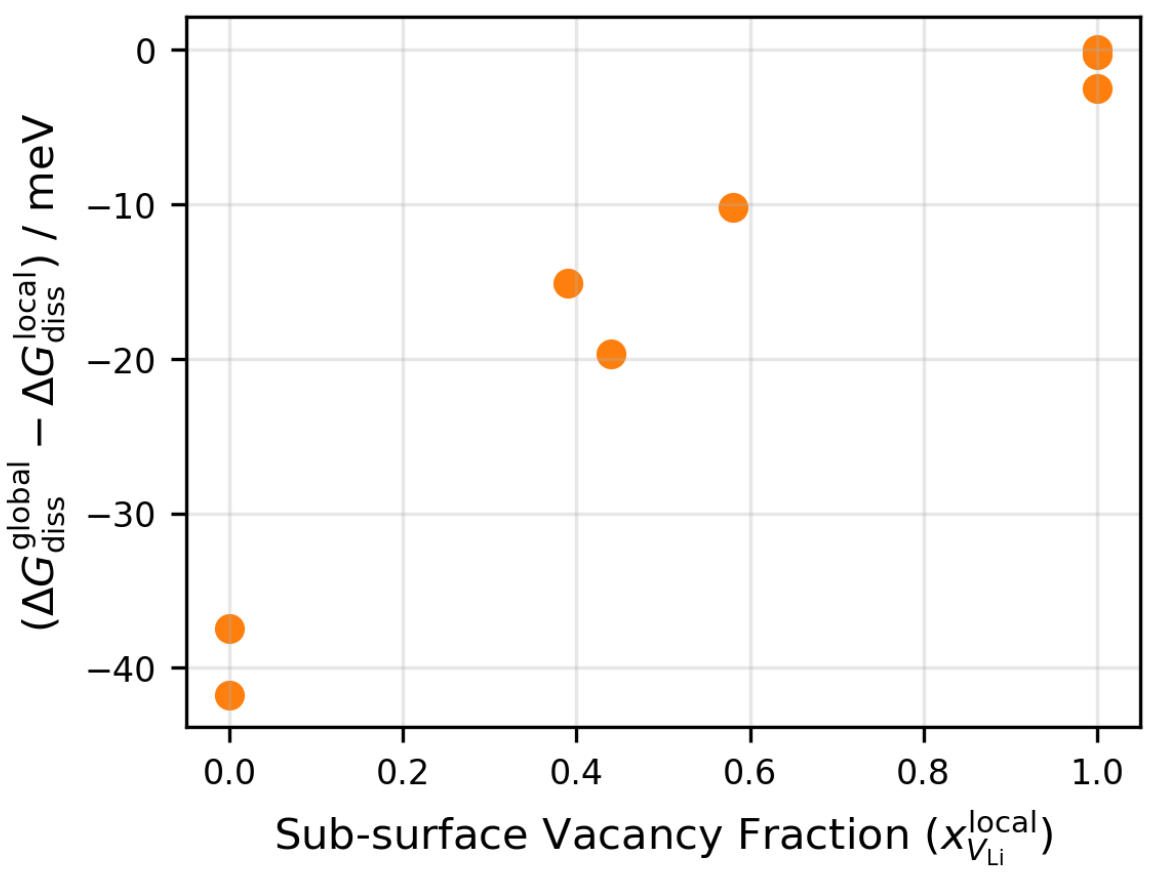

Figure S44: Thermodynamic stabilization of surface protons as a function of sub-surface lithium vacancies. Impact of the sub-surface lithium vacancy fraction ($x_{V_{\mathrm{Li}}}^{\mathrm{local}}$) on the additional thermodynamic stabilization energy, defined as the difference between the globally derived free energy of dissociation and the locally constrained value ($\Delta G_{\mathrm{diss}}^{\mathrm{global}} - \Delta G_{\mathrm{diss}}^{\mathrm{local}}$). The convergent trend highlights that surfaces with low vacancy concentrations rely on global proton delocalization for stabilization, whereas surfaces saturated with vacancies ($x_{V_{\mathrm{Li}}}^{\mathrm{local}} = 1.0$) stabilize protons locally via O–H bond rotation into vacant sites, reducing the additional global stabilization to near zero.

References

[1] M. Eckhoff, P. E. Blöchl, J. Behler, *Physical Review B* **2020**, *101*, 205113.

[2] C. J. Cramer, D. G. Truhlar, *Physical Chemistry Chemical Physics* **2009**, *11*, 10757.

[3] M. Sotoudeh, S. Rajpurohit, P. E. Blöchl, D. Mierwaldt, J. Norpoth, V. Roddatis, S. Mildner, B. Kressdorf, B. Ifland, C. Jooss, *Physical Review B* **2017**, *95*, 235150.

[4] M. Eckhoff, F. Schönewald, M. Risch, C. A. Volkert, P. E. Blöchl, J. Behler, *Physical Review B* **2020**, *102*, 174102.

[5] M. Eckhoff, J. Behler, *The Journal of Chemical Physics* **2021**, *155*, 244703.

[6] P. E. Blöchl, *Physical Review B* **1994**, *50*, 17953.

[7] M. Eckhoff, K. N. Lausch, P. E. Blöchl, J. Behler, *The Journal of Chemical Physics* **2020**, *153*, 164107.

[8] F. L. Hirshfeld, *Theoretica Chimica Acta* **1977**, *44*, 129.

[9] A. Tkatchenko, M. Scheffler, *Physical Review Letters* **2009**, *102*, 073005.

[10] J. P. Perdew, K. Burke, M. Ernzerhof, *Physical Review Letters* **1996**, *77*, 3865.

[11] A. Omranpour, J. Elsner, K. N. Lausch, J. Behler, *ACS Catalysis* **2025**, *15*, 1616.

[12] M. J. Gillan, D. Alfè, A. Michaelides, *The Journal of Chemical Physics* **2016**, *144*, 130901.

[13] K. N. Lausch, R. El Haouari, D. Trzewik, J. Behler, *The Journal of Chemical Physics* **2025**, *163*, 034101.

[14] S. Grimme, A. Hansen, J. G. Brandenburg, C. Bannwarth, *Chemical Reviews* **2016**, *116*, 5105.

[15] P. Jurečka, J. Šponer, J. Černý, P. Hobza, *Physical Chemistry Chemical Physics* **2006**, *8*, 1985.

[16] M. Eckhoff, J. Behler, *Journal of Chemical Theory and Computation* **2019**, *15*, 3793.

[17] M. Eckhoff, J. Behler, *npj Computational Materials* **2021**, *7*, 170.

[18] C. Schran, K. Brezina, O. Marsalek, *The Journal of Chemical Physics* **2020**, *153*, 104105.

[19] C. Schran, F. L. Thiemann, P. Rowe, E. A. Müller, O. Marsalek, A. Michaelides, *Proceedings of the National Academy of Sciences* **2021**, *118*, e2110077118.

[20] J. Behler, M. Parrinello, *Physical Review Letters* **2007**, *98*, 146401.

[21] J. Behler, *International Journal of Quantum Chemistry* **2015**, *115*, 1032.

[22] J. Behler, *Angewandte Chemie International Edition* **2017**, *56*, 12828.

[23] J. Behler, *Chemical Reviews* **2021**, *121*, 10037.

[24] J. Behler, *The Journal of Chemical Physics* **2011**, *134*, 074106.

[25] A. Singraber, T. Morawietz, J. Behler, C. Dellago, *Journal of Chemical Theory and Computation* **2019**, *15*, 3075.

[26] A. Singraber, J. Behler, C. Dellago, *Journal of Chemical Theory and Computation* **2019**, *15*, 1827.

[27] S. Plimpton, *Journal of Computational Physics* **1995**, *117*, 1.

[28] A. P. Thompson, H. M. Aktulga, R. Berger, D. S. Bolintineanu, W. M. Brown, P. S. Crozier, P. J. in 't Veld, A. Kohlmeyer, S. G. Moore, T. D. Nguyen, R. Shan, M. J. Stevens, J. Tranchida, C. Trott, S. J. Plimpton, *Computer Physics Communications* **2022**, *271*, 108171.

[29] S. Nosé, *Molecular Physics* **1984**, *52*, 255.

[30] W. G. Hoover, *Physical Review A* **1985**, *31*, 1695.

[31] P. Schienbein, G. Schwaab, H. Forbert, M. Havenith, D. Marx, *The Journal of Physical Chemistry Letters* **2017**, *8*, 2373.

[32] P. Schienbein, *Journal of Chemical Theory and Computation* **2026**, *22*, 4626.

[33] J. C. Grossman, E. Schwegler, E. W. Draeger, F. Gygi, G. Galli, *The Journal of Chemical Physics* **2004**, *120*, 300.

[34] E. Schwegler, J. C. Grossman, F. Gygi, G. Galli, *The Journal of Chemical Physics* **2004**, *121*, 5400.

[35] P. Schienbein, J. Blumberger, *Physical Chemistry Chemical Physics* **2022**, *24*, 15365.

[36] J. Daru, H. Forbert, J. Behler, D. Marx, *Physical Review Letters* **2022**, *129*, 226001.

[37] N. Stolte, J. Daru, H. Forbert, J. Behler, D. Marx, *The Journal of Physical Chemistry Letters* **2024**, *15*, 12144.

[38] O. Marsalek, T. E. Markland, *The Journal of Physical Chemistry Letters* **2017**, *8*, 1545.

[39] B. Cheng, E. A. Engel, J. Behler, C. Dellago, M. Ceriotti, *Proceedings of the National Academy of Sciences* **2019**, *116*, 1110.

[40] A. Torres, L. S. Pedroza, M. Fernandez-Serra, A. R. Rocha, *The Journal of Physical Chemistry B* **2021**, *125*, 10772.

[41] W. Wagner, A. Pruß, *Journal of Physical and Chemical Reference Data* **2002**, *31*, 387.

[42] J. Akimoto, *Solid State Ionics* **2004**, *172*, 491.

[43] Y. Ni, J. L. Skinner, *The Journal of Chemical Physics* **2015**, *143*, 014502.

[44] P. W. Tasker, *Journal of Physics C: Solid State Physics* **1979**, *12*, 4977.

[45] S. Kim, M. Aykol, C. Wolverton, *Physical Review B* **2015**, *92*, 115411.

[46] J. B. Goodenough, K.-S. Park, *Journal of the American Chemical Society* **2013**, *135*, 1167.

[47] Y. Takahashi, J. Akimoto, Y. Gotoh, K. Dokko, M. Nishizawa, I. Uchida, *Journal of the Physical Society of Japan* **2003**, *72*, 1483.

[48] M. Bianchini, F. Fauth, E. Suard, J.-B. Leriche, C. Masquelier, L. Croguennec, *Acta Crystallographica Section B Structural Science, Crystal Engineering and Materials* **2015**, *71*, 688.

[49] V. W. J. Verhoeven, I. M. De Schepper, G. Nachtegaal, A. P. M. Kentgens, E. M. Kelder, J. Schoonman, F. M. Mulder, *Physical Review Letters* **2001**, *86*, 4314.

[50] A. Stukowski, *Modelling and Simulation in Materials Science and Engineering* **2009**, *18*, 015012.

[51] X. Gao, Y. H. Ikuhara, C. A. J. Fisher, H. Moriwake, A. Kuwabara, H. Oki, K. Kohama, R. Yoshida, R. Huang, Y. Ikuhara, *Advanced Materials Interfaces* **2014**, *1*, 1400143.

[52] H. Liu, T. Shen, X. Zhu, M. Yang, Y. Yao, E. J. Cheng, Y. Zhao, H. Li, S. Li, L. Gu, H. Xia, *Journal of the American Chemical Society* **2026**, *148*, 7388.

[53] B. Ammundsen, D. J. Jones, J. Rozière, H. Berg, R. Tellgren, J. O. Thomas, *Chemistry of Materials* **1998**, *10*, 1680.

[54] B. Roux, *Computer Physics Communications* **1995**, *91*, 275.

[55] A. R. Leach, *Molecular Modelling: Principles and Applications*, Prentice Hall, 2 edition **2001**.

[56] D. Marx, M. E. Tuckerman, J. Hutter, M. Parrinello, *Nature* **1999**, *397*, 601.
